# Project Gel a Randomized Rectal Microbicide Safety and Acceptability Study in Young Men and Transgender Women

**DOI:** 10.1371/journal.pone.0158310

**Published:** 2016-06-30

**Authors:** Ian McGowan, Ross D. Cranston, Kenneth H. Mayer, Irma Febo, Kathryn Duffill, Aaron Siegel, Jarret C. Engstrom, Alexyi Nikiforov, Seo-Young Park, Rhonda M. Brand, Cindy Jacobson, Rebecca Giguere, Curtis Dolezal, Timothy Frasca, Cheng-Shiun Leu, Jill L. Schwartz, Alex Carballo-Diéguez

**Affiliations:** 1 University of Pittsburgh, Pittsburgh, Pennsylvania, United States of America; 2 Magee-Womens Research Institute, Pittsburgh, Pennsylvania, United States of America; 3 Fenway Institute, Fenway Health, Boston, Massachusetts, United States of America; 4 Beth Israel Deaconess Medical Center, Harvard Medical School, Boston, Massachusetts, United States of America; 5 University of Puerto Rico Medical Sciences Campus, Department of Pediatrics, Gama Project, San Juan, Puerto Rico; 6 Columbia University and NY State Psychiatric Institute, HIV Center for Clinical and Behavioral Studies, New York, New York, United States of America; 7 CONRAD, Arlington, Virginia, United States of America; University of Toronto, CANADA

## Abstract

**Objectives:**

The purpose of Project Gel was to determine the safety and acceptability of rectal microbicides in young men who have sex with men (MSM) and transgender women (TGW) at risk of HIV infection.

**Methods:**

MSM and TGW aged 18–30 years were enrolled at three sites; Pittsburgh, PA; Boston, MA; and San Juan, PR. Stage 1A was a cross-sectional assessment of sexual health and behavior in MSM and TGW. A subset of participants from Stage 1A were then enrolled in Stage 1B, a 12-week evaluation of the safety and acceptability of a placebo rectal gel. This was followed by the final phase of the study (Stage 2) in which a subset of participants from Stage 1B were enrolled into a Phase 1 rectal safety and acceptability evaluation of tenofovir (TFV) 1% gel.

**Results:**

248 participants were enrolled into Stage 1A. Participants’ average age was 23.3 years. The most common sexually transmitted infection (STIs) at baseline were Herpes simplex (HSV)-2 (16.1% by serology) and rectal *Chlamydia trachomatis* (CT) (10.1% by NAAT). 134 participants were enrolled into Stage 1B. During the 12 week period of follow-up 2 HIV, 5 rectal CT, and 5 rectal *Neisseria gonorrhea* infections were detected. The majority of adverse events (AEs) were infections (N = 56) or gastrointestinal (N = 46) and were mild (69.6%) or moderate (28.0%). Of the participants who completed Stage 1B, 24 were enrolled into Stage 2 and randomized (1:1) to receive TFV or placebo gel. All participants completed Stage 2. The majority of AEs were gastrointestinal (N = 10) and of mild (87.2%) or moderate (10.3%) severity.

**Conclusions:**

In this study we were able to enroll a sexually active population of young MSM and TGW who were willing to use rectal microbicides. TFV gel was safe and acceptable and should be further developed as an alternative HIV prevention intervention for this population.

**Trial Registration:**

ClinicalTrials.gov NCT01283360

## Introduction

Young men who have sex with men (MSM) are one of the key groups at risk of HIV acquisition in the United States. Recent data have demonstrated an increase of 48% in HIV infections in the 12–24 year age group between 2003 and 2014. The vast majority of these occurred in young Black or Latino MSM [1].TGW also continue to have unacceptably high rates of HIV infection [2] especially those who are involved in sex work [3]. Adolescents and young adults with HIV infection fare poorly in the US health care system; their rates of diagnosis of HIV infection, access to care, initiation of antiretroviral therapy, and viral suppression are all lower than among older populations [4]. These statistics reinforce the need to develop HIV prevention strategies that will be acceptable to, and used by, young MSM and TGW.

Topical microbicides are products that can be applied to the vaginal or rectal mucosa with the goal of preventing or significantly reducing the risk of sexually transmitted infection (STI) transmission including HIV infection [5]. The CAPRISA 004 trial demonstrated the efficacy of pericoital tenofovir (TFV) 1% gel as a vaginal microbicide [6] although two subsequent TFV vaginal gel studies did not demonstrate efficacy, primarily because of suboptimal adherence [7;8]. Multiple early phase trials of antiretroviral rectal microbicides have been completed or are ongoing [9]. Moreover, since both TFV and cyanovirin have demonstrated efficacy in non-human primate rectal challenge models [10;11] there is a strong scientific rational for evaluation of rectal microbicides in human efficacy trials.

Preliminary research findings concerning the potential acceptability of a rectal microbicide have been highly encouraging [12–14]. Carballo-Diéguez et al. found that a sexually active cohort of middle aged MSM rated volumes of up to 35 ml of gel acceptable for use during anal intercourse [15]. Formative research from Peru has suggested that MSM and TGW might find oral and rectal antiretroviral pre-exposure prophylaxis (PrEP) highly acceptable [16]. However, information is lacking concerning microbicide acceptability in younger populations, particularly ethnic minority adolescent MSM and TGW.

Anorectal symptoms are common in the general population which may require medical attention or, more commonly, are self-medicated with over-the-counter preparations [17;18]. Regardless of etiology, anorectal conditions such as hemorrhoids, anal fissures, abscesses, and dermatoses frequently have a shared presentation with symptoms of itch, pain, bleeding, swelling, or development of a mass lesion [17]. Condomless RAI places the anorectum at risk of a number of STIs that may be symptomatic or asymptomatic that includes; *Neisseria gonorrhoea* (GC), *Chlamydia trachomatis* (CT), *Lymphogranuloma venereum* (LGV), syphilis, Herpes simplex type 2, and human papillomavirus (HPV) [19–23]. Anorectal symptoms are more common in MSM who practice RAI and RAI *per se* can cause anal fissures, anorectal ulcerations, hemorrhoids, and anorectal tears [24–26]. Anogenital STIs are associated with increased rates of incident HIV-1 infection; conversely, HIV-incidence is reduced when these infections are treated [27–29]. From a clinical perspective, there are limited published data on the prevalence of STIs, HPV, HPV-associated anal dysplasia, anal fissures, and other anorectal pathologies or trauma that facilitate HIV infection in young MSM, particularly those of ethnic minority extraction [30].

Certain practices are known to damage the rectal mucosa and render it more vulnerable to infection. For example, the use of hyperosmolar rectal lubricants [31] or certain enemas [32] can result in epithelial sloughing; furthermore, lubricants containing N-9 that transiently damages rectal epithelium continue to be used among some MSM [33]. There is a need to identify the extent of these behavioral practices and how they may contribute to the vulnerability of sexually active MSM.

The overall goal of this study was to evaluate the rectal acceptability, adherence, and safety of rectal microbicides in young MSM and TGW and to characterize associated behaviors and acquisition of STIs that might affect the effectiveness of antiretroviral rectal microbicides and to assess adherence to tenofovir gel in a phase I safety trial.

## Materials and Methods

The protocol for this trial and supporting CONSORT checklist are available as supporting information; see Checklist (S1 File) and Protocol (S2 File).

### Ethics Statement

The study was approved by the University of Pittsburgh Institutional Review Board (IRB) on the 4^th^ October, 2010, the University of Puerto Rico IRB on the 15^th^ October, 2010, and Fenway Health IRB on the 30^th^ July, 2010. All subjects provided written informed consent. The trial was registered at ClinicalTrials.gov on the 1^st^ November, 2010, number # NCT01283360 and is in compliance with the CONSORT 2010 recommendations for reporting of trial results (www.consort-statement.org) [34;35].

### Study Design and Participants

**Fig 1:**
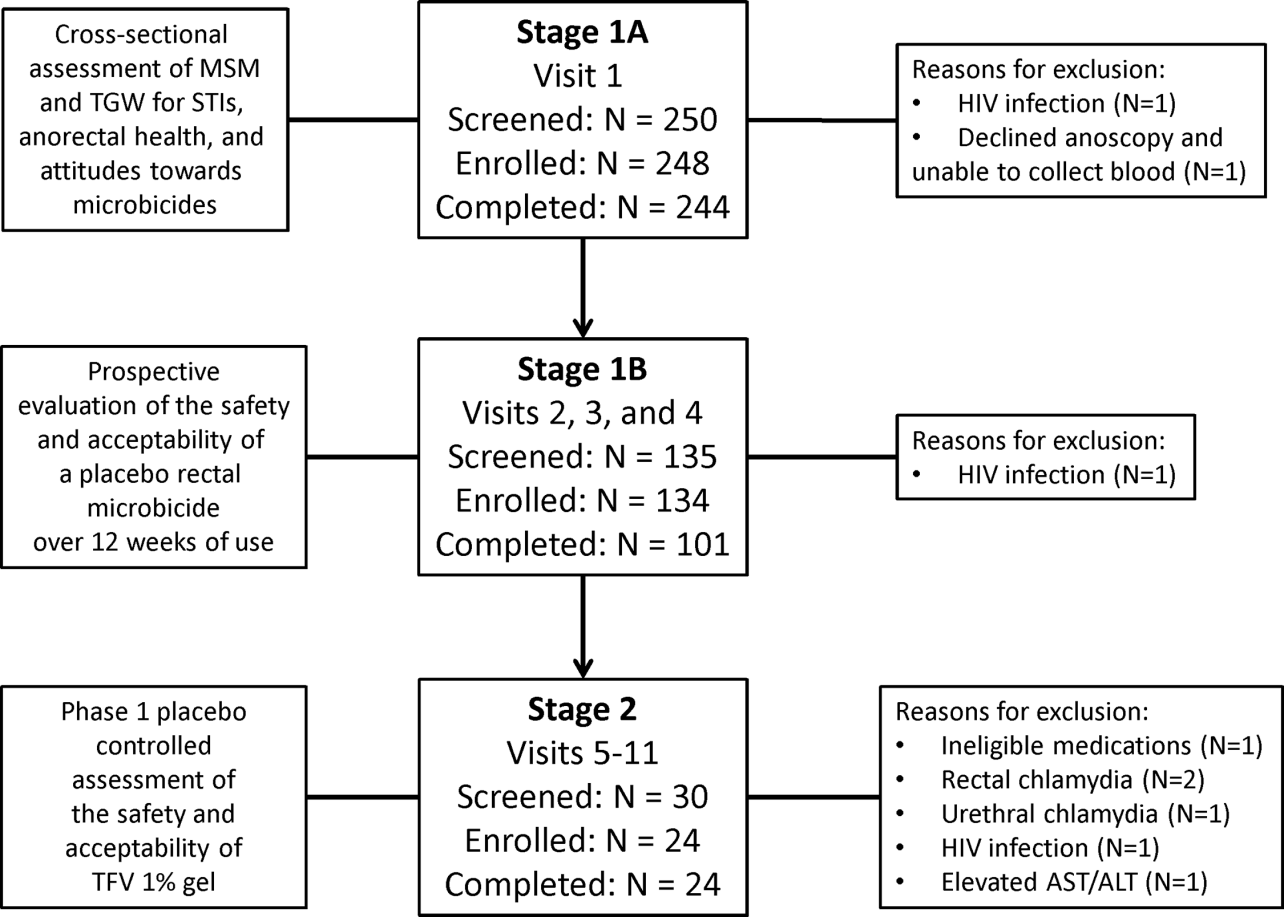
Overview of study design and visit schedule.

Project Gel took place in two time periods. The first recruitment period took place between December 2010 and June 2012, focused on young MSM. All three sites used a combination of established research registries and community outreach events within the MSM and TGW communities to recruit potential study participants. The second one, with recruitment occurring between April and July 2013, focused on sex workers and included MSM and TGW. Recruitment of sex workers used similar strategies but intensified outreach activity within the sex worker community. The study consisted of three stages (Fig 1) with target sample size for each stage determined by the primary objective of each stage. By study design, after completing a brief screening evaluation, 280 eligible participants, including up to 40 sex workers, were to be enrolled into Stage 1A and to undergo a baseline medical evaluation for both history and presence of STIs and anorectal health pathologies or injuries, as well as a detailed, web-based, baseline behavioral assessment. The first 140 eligible participants, including up to 20 sex workers, reporting at least one occasion of unprotected receptive anal intercourse (RAI) in the previous 3 months were to be enrolled into Stage 1B. In Stage 1B participants were asked to apply a hydroxyethyl cellulose (HEC) placebo gel [36] rectally prior to each episode of RAI over a 3-month period, reporting each use via an interactive voice response system (IVRS); they were asked to complete a web-based questionnaire and a video teleconference at the end of the 3 months and to bring in both used and unused applicators to the clinic to be counted. The first 24 eligible participants completing Stage 1B, excluding sex workers, were to be invited to enroll in Stage 2 and randomized to receive either TFV 1% gel or HEC placebo gel. Following a Baseline visit, participants returned to the clinic where a single dose of the study gel was administered. Within approximately 30 minutes, rectal swab and rectal biopsy specimens were obtained via anoscopy. After a one-week recovery period, participants returned to the clinic for assessment. If no significant adverse events (AEs) were reported, they self-administered once-daily outpatient doses of the study gel for 7 days, after which they returned to the clinic for evaluation and specimen collection. The study was conducted at three clinical sites: the University of Pittsburgh in Pittsburgh, Pennsylvania; Fenway Health in Boston, Massachusetts; and the University of Puerto Rico in San Juan.

### Study population

Inclusion and exclusion criteria varied by study stage. Stage 1A required that participants were biologically male, aged between 18 and 30 years at time of screening, HIV-uninfected or of unknown HIV status, had a history of consensual RAI in the previous month, and a history of one episode of condomless RAI in the previous year. Sex workers were also required to have a history of two episodes of transactional sex within the two months prior to study entry. There were no other specific exclusion criteria for Stage 1A other than the clinical investigator determining that the participant was not suitable for the study, unless participants had symptomatic rectal STIs. Stage 1B required that participants had completed Stage 1A, were HIV-uninfected, and had a history of at least one episode of unprotected RAI in the previous three months. Exclusion criteria for Stage 1B included active rectal STI requiring treatment, hepatitis B surface antigen positivity, a history of inflammatory bowel disease, and an intention to have unprotected RAI with HIV-infected partners. Inclusion criteria for Stage 2 included completion of Stages 1A and 1B and being HIV-uninfected. Exclusion criteria included meeting any Stage 1B exclusion criteria, reporting a history of transactional sex, undergoing or completion of gender reassignment surgery, Grade 2 or higher laboratory abnormalities defined by the DAIDS toxicity table, abnormalities of the colorectal mucosa, significant gastrointestinal symptoms (such as a history of rectal bleeding), or a requirement to use drugs that were likely to increase the risk of bleeding following mucosal biopsy were excluded from the study.

### Study products

**Fig 2:**
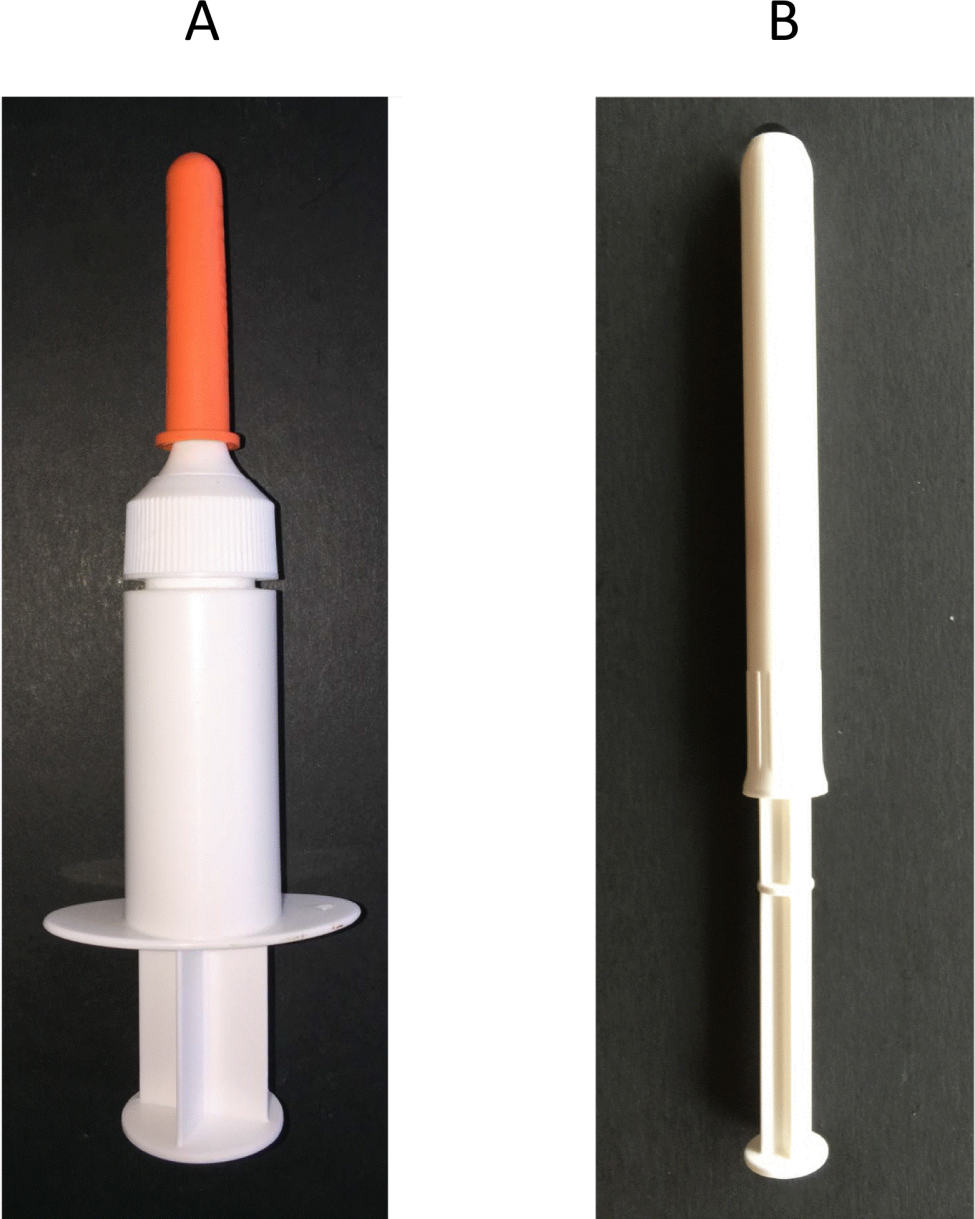
Microbicide applicators used in Project Gel. (A) A rectal specific applicator was used to deliver the placebo gel in Stage 1B of the study [37]. (B) The HTI applicator was used to deliver placebo or tenofovir gel in Stage 2 of Project Gel.

The TFV and HEC placebo gels were manufactured under direction from CONRAD (Arlington, VA) by DPT Laboratories (San Antonio, TX). A rectal specific applicator was used to deliver the HEC gel in stage 1B [37] (Fig 2). The HTI vaginal applicator (HTI Plastics, Lincoln, NE) was used rectally to deliver the both the HEC placebo and TFV gels in Stage 2 (Fig 2). Each opaque pre-filled applicator was packaged with a plunger and labeled with a code to preserve the identity of the formulation. Each pre-filled applicator contained a dose of approximately 4 mL of TFV gel or the HEC placebo. The pre-filled applicators were shipped directly to study site pharmacies and were stored by and dispensed from the site pharmacy. For Stage 2, randomization codes were generated by the study statistician and pharmacist. The study investigators and participants were blinded to study product assignment.

### Outcome measures

Study objectives were stratified by study stage (Stage 1AB and Stage 2) and divided into clinical and behavioral objectives.

#### Stage 1AB Clinical Objectives

Primary objective was to determine the prevalence of STIs and anal and rectal pathologies that may facilitate HIV infection

Secondary objective was to determine whether standard anoscopy with examination by the naked eye is as effective in identifying anorectal pathologies as high-resolution anoscopy (HRA)

#### Stage 1AB Behavioral Objectives

Primary objective was to determine the acceptability of and use-adherence to a placebo gel delivered rectally with a rectal delivery device

Secondary objective was to determine the prevalence of behavioral practices associated with anal intercourse that may detract from microbicide effectiveness

Tertiary objective was to determine the feasibility of recruitment and retention of MSM and TGW at risk of HIV, such as sex workers, for rectal microbicide studies, and to assess their likelihood to use non-condom based HIV prevention strategies

#### Stage 2 Clinical Objectives

Primary objective was to evaluate the safety of TFV 1% gel when applied rectally

Exploratory objective was to determine whether use of TFV 1% gel is associated with rectal mucosal damage

#### Stage 2 Behavioral Objectives

Primary objective was to determine the acceptability of and use-adherence to a placebo gel or TFV 1% gel delivered rectally with a vaginal delivery device

Secondary objective was to determine the prevalence of behavioral practices associated with anal intercourse that may detract from microbicide effectiveness

### Data collection and follow-up procedures

#### Clinical assessments

After obtaining informed consent at Visit 1 a medical history was collected, a targeted physical examination was performed including digital rectal examination and anoscopy. HRA was also performed on participants enrolled in Pittsburgh and Boston. Clinical examination was subsequently conducted at Visits 4 and 5 and at other study visits if clinically indicated. Anoscopy was conducted at screening and at Visits 1, 3–7, 9, and 10 (S1 Table).

Emergent AEs were graded using the Division of AIDS Table for Grading the Severity of Adult and Pediatric Adverse Events, Version 1.0, December 2004 as well as Addendum 1 and 3 (*Female Genital and Rectal Grading Table for Use in Microbicide Studies* (http://rsc.tech-res.com/safetyandpharmacovigilance/). In cases where an AE was covered in both tables, the *Female Genital or Rectal Grading Table for Use in Microbicide Studies* was the grading scale utilized.

#### Laboratory assessments

At screening a buccal swab was collected for HIV testing, and an oral rinse specimen was collected for HPV genotyping. Anorectal swabs were taken for *Chlamydia trachomatis* (CT)/*Neisseria gonorrhea* (GC) nucleic acid amplification testing (NAAT) and HPV genotyping. Urine was also collected for CT/GC NAAT. Blood was collected for safety labs (complete blood count, urea nitrogen, creatinine, alanine aminotransferase, and aspartate aminotransferase) and serology (syphilis, HIV-1, hepatitis B, hepatitis C, and herpes simplex virus (HSV) 1 and 2). STI screening was repeated at Visits 4, 5, and 10 and at other study visits if clinically indicated. Merocel sponges (Beaver Visitec, Waltham, MA, USA) were used to collect rectal fluid for proteomic assessment at Visits 6, 7, and 10. Rectal biopsies were collected via anoscopy for histology, flow cytometry, and transcriptomics at Visits 6, 7, and 10.

A qualitative scoring system developed for inflammatory bowel disease (IBD) research [38] and adapted for use in RM trials [39] was used to characterize potential product-associated injury with a scale of 1 (normal) to 5 (mucosal erosion or ulceration). Mucosal mononuclear cells (MMC) were isolated from rectal biopsies using a combination of mechanical and enzyme digestion as previously described [40]. Flow cytometric analysis was performed on a BD™ LSRFortessa cytometer (BD Biosciences, San Jose, CA All data were stored in list mode and analyzed with BD™ FACSDIVA operating system and Flow Jo (Tree Star, Inc., Ashland, OR). All antibodies were purchased from BD Biosciences, San Jose, CA (PerCP-CD45, Clone 2D1; Pacific Blue-CD3, Clone UCHT1; PE-Cy7-CD4, Clone SK3; APC-H7-CD8, Clone SK1; FITC-CD69, Clone FN50 APC-CD195 (CCR5), Clone 2D7/CCR5 and PE-CD38, Clone HB7) and titrated under assay conditions to determine an optimum saturating dilution. Cells were stained with LIVE/DEAD® Fixable Aqua stain fluorescence (Life Technologies, Eugene, OR) to define viable cells. Proteomic characterization of rectal fluid was performed using label-free mass spectrometry [41] and transcriptomic analysis of rectal tissue was performed using RNA-Seq-based differential gene expression analysis RNA-Seq was performed using the Illumina HiSeq 2500 (Illumina Inc., San Diego, CA, USA) and TruSeq workflow platform (Illumina Inc., San Diego, CA, USA). Both proteomic and transcriptomic data from Project Gel will be reported in a secondary manuscript

#### Behavioral assessments

Web-based Computer-Assisted Self-Interviews (CASIs) were conducted at screening and at Visits 4 and 10. The CASI interviews included baseline behavioral and product acceptability questionnaires. The baseline questionnaire included questions on sexual behavior during the prior three months, lubricant use for RAI, likelihood to use a rectal microbicide that provided some protection against HIV in every occasion of RAI, and awareness and use of post-exposure prophylaxis (PEP) and pre-exposure prophylaxis (PrEP). The product acceptability questionnaires included questions on gel’s characteristics, application process and applicator preferences, product use, product liking and likelihood to use if proven effective against HIV.

Video teleconferences were conducted at Visits 2, 4, 9, and 10 to discuss issues related to product use including acceptability, partner reactions, and the likelihood that the participant would use microbicides in the future. Interviews were conducted in English or Spanish by an interviewer (“Alex”). Product adherence was assessed using an interactive voice response system (IVRS) to which participants were asked to call in after each product use in Stage 1B and Stage 2. Voice prompts were recorded by Alex. Modest monetary incentives were used to encourage regular calls. Participants also had the option to send emails to Alex but were counselled that requests for clinical care should be directed to the clinic staff. The detailed results of the behavioral component of Project Gel MSM cohort have been published [37;42–44] and will only be briefly summarized in this manuscript. The behavioral findings with respect to the sex worker cohort will be published in a separate manuscript given that it was a very different population from a behavioral perspective.

### Statistical analysis

Descriptive statistics were used to summarize participant characteristics. For categorical variables, the numbers and the proportions were tabulated; for continuous variables, the mean, and standard deviation were reported. To assess the change of an endpoint from baseline to post-visit levels, McNemar’s test (for categorical variables) or paired *t*-test (for continuous variables) was used. To assess the difference of certain endpoints between groups, Chi-square test or Fisher’s exact test was used for categorical variables depending on the expected cell count; t-test or linear regression were used for continuous variables; nonparametric methods such as Wilcoxon rank-sum test were used when the sample size was small and data were non-normal. The longitudinal data were analyzed using linear mixed model with intervention group as a fixed effect and subject as a random effect, to allow intra-subject correlation. The rationale for sample size in each stage of the study is outlined in detail in the study protocol (available as supporting information).

## Results

### Stage 1A

#### Enrollment and retention

A total of 248 participants were enrolled into Stage 1A of Project Gel. Almost all (94%) were MSM and 6% were sex workers. Of the 15 sex workers, seven identified as male, four as female, and four as transgender. 12 of the 15 sex workers were recruited from the San Juan site. The average age of study participants was 23.3 ± 3.4 years and study demographics are summarized in Table 1. 244/248 (98%) participants completed Stage 1A. 135 participants were screened, 134 were enrolled, and 101 completed Stage 1B. 30 participants were screened, 24 were enrolled, and 24 completed Stage 2 of the study.

**Table 1:** Project Gel Demographics.

Variables	Overall	Pittsburgh	Boston	San Juan
	(N = 248)	(N = 63)	(N = 88)	(N = 97)
**Age**	23.3 ± 3.4	22.7 ± 3.3	23.8 ± 3.3	23.1 ± 3.4
**Race**				
White or European American	119 (48.0%)	45 (71.4%)	54 (61.4%)	20 (20.6%)
Black or African American	26 (10.5%)	15 (23.8%)	8 (9.1%)	3 (3.1%)
Asian	3 (1.2%)	1 (1.6%)	2 (2.3%)	0 (0.0%)
Native Hawaiian or other Pacific Islander	22 (8.9%)	0 (0.0%)	4 (4.6%)	18 (18.6%)
Others	78 (31.5%)	2 (3.2%)	20 (22.7%)	56 (57.7%)
**Ethnicity**				
Hispanic	111 (44.8%)	1 (1.6%)	15 (17.1%)	95 (97.9%)
Non-Hispanic	136 (54.8%)	61 (96.8%)	73 (83.0%)	2 (2.1%)
Refused to answer	1 (0.4%)	1 (1.6%)	0 (0.0%)	0 (0.0%)

#### Sexually transmitted infections

Positive syphilis serology was found in 14 participants (5.7%) of which seven infections (2.8%) were deemed to be new infections. Positive HSV serology was found in a total of 170 participants (HSV-1; 130 participants (52.4%) and HSV-2; 40 participants (16.1%)). Five participants had HIV infection and 1 had evidence of HCV infection. Oral HPV infection was identified in four participants (1.7%) and anal HPV in 127 participants (55.5%). Urethral GC was diagnosed in one participant (0.4%) and rectal GC in three participants (1.2%). Urethral CT infection was diagnosed in four participants (1.6%) and rectal CT in 26 participants (10.5%). STIs were most common in the participants enrolled at the San Juan site (Table 2).

**Table 2:** Sexually Transmitted Infections Diagnosed in Stage 1A.

	Overall	Pittsburgh	Boston	San Juan
	N = 248	N = 63)	N = 88)	(N = 97)
**HIV-1**	5/248 (2.0%)	2/63 (3.2%)	0/88 (0.0%)	3/97 (3.1%)
**Herpes simplex virus-1**	130/248 (52.4%)	22/63 (34.9%)	46/88 (52.3%)	62/97 (63.9)
**Herpes simplex virus-2**	40/248 (16.1%)	10/63 (15.9)	9/88 (10.2%)	21/97 (21.7%)
**Syphilis**	7/248 (2.8%)	1/63 (1.6%)	1/88 (1.1%)	5/97 (5.2%)
**Hepatitis C virus**	1/248 (0.4%)	0/63 (0.0%)	1/88 (1.1%)	0/97 (0.0%)
**Oral human papillomavirus**	4/235 (1.7%)	0/60 (0.0%)	4/82 (4.9%)	0/93 (0.0%)
**Anal human papillomavirus**	127/229 (55.5%)	34/63 (54.0%)	56/81 (69.1%)	37/85 (43.5%)
**Urethral *Neisseria gonorrhea***	1/248 (0.4%)	0/63 (0.0%)	0/88 (0.0%)	1/97 (1.0%)
**Rectal *Neisseria gonorrhea***	3/248 (1.2%)	0/63 (0.0%)	3/88 (3.4%)	0/97 (0.0%)
**Urethral *Chlamydia trachomatis***	4/248 (1.6%)	0/63 (0.0%)	1/88 (1.1%)	3/97 (3.1%)
**Rectal *Chlamydia trachomatis***	26/248 (10.5%)	2/63 (3.2%)	7/88 (8.0%)	17/97 (17.5%)

#### Anorectal examination

Visual anal examination was normal in 88.5% of participants (Table 3). Visualized abnormalities included warts (6.2%), hemorrhoids (1.2%), and fissures (0.8%). Rectal examination was normal in 95.1% of participants. Reported abnormalities included mucosal erythema (3.3%) and hemorrhoids (0.8%). Use of high resolution anoscopy (HRA), which was limited to the Pittsburgh and Boston sites, was normal in 70.8% of participants who underwent the procedure. Abnormalities included warts (14.6%), hemorrhoids (2.8%), bleeding (2.1%), and fissure (1.4%). HRA was significantly more sensitive in detecting anal abnormalities (29.2%) than direct visual inspection (14.6%), p = 0.0028.

**Table 3:** Anorectal Examination in Stage 1A Participants.

	Overall	Pittsburgh	Boston	San Juan
	(N = 248)	(N = 63)	(N = 88)	(N = 97)
**Normal standard visual anal examination**	216 (88.5%)	52 (82.5%)	73 (86.9%)	91 (93.8%)
**Not Done**	4 (1.6%)	0 (0.0%)	4 (4.6%)	0 (0.0%)
**Abnormal standard visual anal examination**	28 (11.5%)	11 (17.5%)	11 (13.1%)	6 (6.2%)
Warts	15 (6.2%)	9 (14.3%)	3 (3.6%)	3 (3.1%)
Fissure	2 (0.8%)	0 (0.0%)	1 (1.2%)	1 (1.0%)
Ulceration	0 (0.0%)	0 (0.0%)	0 (0.0%)	0 (0.0%)
Bleeding	2 (0.8%)	0 (0.0%)	2 (2.4%)	0 (0.0%)
Hemorrhoids	3 (1.2%)	0 (0.0%)	2 (2.4%)	1 (1.0%)
Other	6 (2.5%)	2 (3.2%)	3 (3.6%)	1 (1.0%)
**Normal rectal examination**	232 (95.1%)	62 (98.4%)	83 (98.8%)	87 (89.7%)
**Not Done**	4 (1.6%)	0 (0.0%)	4 (4.6%)	0 (0.0%)
**Abnormal rectal examination**	12 (4.9%)	1 (1.6%)	1 (1.2%)	10 (10.3%)
Erythema	8 (3.3%)	0 (0.0%)	0 (0.0%)	8 (8.3%)
Friability	1 (0.4%)	0 (0.0%)	0 (0.0%)	1 (1.0%)
Discharge	0 (0.0%)	0 (0.0%)	0 (0.0%)	0 (0.0%)
Ulceration	0 (0.0%)	0 (0.0%)	0 (0.0%)	0 (0.0%)
Bleeding	1 (0.4%)	1 (1.6%)	0 (0.0%)	0 (0.0%)
Abnormal Vessels	0 (0.0%)	0 (0.0%)	0 (0.0%)	0 (0.0%)
Hemorrhoids	2 (0.8%)	0 (0.0%)	1 (1.2%)	1 (1.0%)
Other	0 (0.0%)	0 (0.0%)	0 (0.0%)	0 (0.0%)

#### Baseline behavioral questionnaire

Participants in the MSM cohort were asked their likelihood to use a microbicide gel if it provided some protection against HIV. Responses were provided on scales ranging from 1 = very unlikely to 10 = very likely. At baseline, most participants expressed high likelihood to use it every time they had RAI (M = 9.18, SD = 1.51), to use it with a lover (M = 8.24, SD = 2.53), with one-night stands (M = 9.35, SD = 1.70), with other partners (M = 9.15, SD = 1.84). Participants were also asked about their likelihood to use a rectal microbicide if it came in the form of a suppository or a rectal enema. In these cases, likelihood to use was still high (M = 7.67, SD = 2.71, and M = 6.56, SD = 3.10, respectively), although relatively less likely than if the microbicide were a gel.

At baseline, we also measured awareness of, and experiences with, post-exposure prophylaxis (PEP) and pre-exposure prophylaxis (PrEP) via web-based CASI. Among the initially recruited MSM participants, 41% had heard of PEP, ranging from 16% in San Juan to 36% in Boston. Three had used PEP, none had used PrEP. Interest in both was high, with intentions to use PEP and PrEP respectively at 9.1 and 7.7 (10-point scale) [43].

### Stage 1B

#### Enrollment and retention

A total of 134 participants were enrolled into Stage 1B of Project Gel and 101 completed this phase of the study. 92.5% were MSM from the initial cohort, 7.5% were from the sex worker cohort, and 4.5% identified as female or transgender. The average age of study participants was 23.1 ± 3.3 years. 75.4% of the participants completed follow-up in Stage 1B.

#### Adverse events

A total of 214 AEs were reported by the 134 participants in Stage 1B of the study. The majority of AEs were graded as mild (Grade 1; 69.2%) or moderate (Grade 2; 28.0%). The most common AEs were gastrointestinal (N = 46) with hemorrhoids (17%), constipation (13%), and rectal/anal itching (8%) being most commonly reported and infections (N = 53), with STIs accounting for 43% of the infections. Two Grade 3 and four Grade 4 AEs were not related to study product.

#### Sexually transmitted infections

At Visit 3 five participants were found to have rectal CT infection and three had rectal GC. At Visit 4 one case of syphilis, two new HIV infections, two cases of rectal CT, and two cases of rectal GC were identified. All Visit 4 infections occurred in participants enrolled at the San Juan site.

#### Anorectal examination

Visual anal examination was normal in 76.2% and 89.5% of participants at Visits 3 and 4 respectively. Rectal examination was normal in 79.5% and 89.5% of participants at Visits 3 and 4 respectively. As in Stage 1A, the most commonly reported abnormalities included warts, hemorrhoids, and fissure.

#### Behavioral data

For the MSM group recruited in the first period, adherence to product use was measured via CASI, IVRS, and by applicator count. These three sources were compared among participants who completed Stage 1B (N = 95). Based on CASI, 83 participants had RAI using gel on 82.4% of occasions (SD 26.7; 0–100). Based on IVRS, 88 participants had RAI (median 10 occasions) using gel on 87.9% of occasions (SD 20.0; 20–100). By applicator counts the median gel use was 12. Those who did not use the product consistently (n = 40) adduced not having it with them (85%), forgetting to use it (48%), not wanting to use it (13%), partner refusal (10%) and gel messiness (10%) [37;42].

MSM participants recruited in the first period of the study also reported via CASI on product acceptability (i.e., satisfaction with applicator and gel, perceived gel side effects; and sexual satisfaction when gel was used) and likelihood of future microbicide use. Using linear regression, we found a positive association between future use intentions and applicator satisfaction (b = .33; p< .001), and based on an interaction effects model, greater gel satisfaction was associated with increased future use intention. However, the strength of this relationship was magnified when men reported greatest satisfaction with the rectal applicator [45].

We also assessed acceptability of the gel and likelihood of future use across partner types among MSM recruited in the first period. Sixty-two men (71.3%) reported gel use with a lover (i.e., spouse-equivalent or boyfriend), 32 (36.8%) with a one-night stand (i.e., a man with whom you had sex once), and 29 (33.3%) with an "other" male partner. While gel acceptability was high across partner types, use with lovers was facilitated by trust and familiarity; yet, trust made participants feel that protection was less necessary. Conversely, participants expressed high likelihood of future gel use with one-night stands, whom they perceived as riskier; yet, they felt less comfortable discussing gel with them during the trial, often resorting to covert use or foregoing gel [44]. We also assessed men's history of lubricant use and acceptability of the gel's qualities as a lubricant during sex. Their preferences varied as to product viscosity, durability, residue, and mode of application [46].

### Stage 2

#### Enrollment and retention

A total of 24 participants were enrolled into and completed Stage 2 of Project Gel. All 24 participants were MSM with an average age of 23.6 ± 3.4 years.

#### Adverse events

AEs were generally mild (Grade 1, N = 34, 87.2% of all AEs) or moderate (Grade 2, N = 4, 10.3% of all AEs). One Grade 3 event of abdominal pain occurred in a patient receiving TFV gel and was considered unrelated to study product (Table 4). Gastrointestinal adverse events were the most common AEs (N = 10, 25.6% of all AEs). The most common gastrointestinal AE was diarrhea (2 cases). The majority of AEs (9/10; 90.0%) were mild. There were no significant differences in the proportion of participants with ≥ Grade 2 or higher adverse events across the arms of the study. There were no product discontinuations related to AEs. More detailed safety data are provided in S2 Table.

**Table 4:** Overall Summary of Adverse Events in Stage 2 Participants.

Severity	Overall		RGVF TFV		Placebo		P Value*
	N	%	N	%	N	%	
**Grade 1**	34	87.2	21	87.5	13	86.7	
**Grade 2**	4	10.3	2	8.3	2	13.3	
**Grade 3**	1	2.6	1	4.2	0	0.0	
**Grade 4**	0	0.0	0	0.0	0	0.0	
**Total**	39	100	24	100	15	100	
**Participants with ≥ Grade 2 Events**	3	12.5	2	16.7	1	8.3	> 0.999

* P-value from two-sided Fisher’s exact test comparing the proportion of participants with at least one adverse event with a severity grade of 2 or more

#### Sexually transmitted infections

There were three new infections documented during Stage 2. One participant acquired HIV infection which was documented at Visit 9 and there was one case of rectal GC and one case of urethral CT infection documented at Visit 10.

#### Anorectal examination

Visual anal examination detected two cases of anal warts and one case of rectal bleeding during Stage 2. Rectal examination was otherwise normal throughout Stage 2.

#### Laboratory data

There were no significant differences in the majority of gut associated lymphoid tissue (GALT) T cell phenotype characterized by flow cytometry. One exception was CD4+/CD69+ T lymphocytes which were significantly reduced between Visits 6 and 7 in the HEC placebo arm (88.9 ± 5.3% versus 84.6 ± 7.4%, p = 0.02). There were no significant changes in the histology inflammation scores. Proteomic and transcriptomic analyses of rectal fluid and tissue respectively will be reported elsewhere (manuscript in preparation).

#### Behavioral data

Concerning adherence to product use in Stage 2, 22 of 24 participants used the applicator as prescribed on all seven occasions. Two participants reported only using six applicators; one in each arm of the study. Reasons for not using gel for the two participants who skipped one application were “I forgot” for both participants, plus “I didn’t have the gel with me” for the placebo participant.

Table 5 presents product acceptability for the Stage 2 participants. Acceptability ratings for TFV 1% gel did not differ from ratings for the HEC placebo gel. Most ratings on gel characteristics, applicator and applications process, product use, and liking the product were on the higher end of 10-point scales, indicating high acceptability. In some cases participants did not comment on certain characteristics of the gels, probably because delivering it inside the rectum with an applicator prevented them from assessing its color or scent. Participants were not asked to taste the gel; yet one participant scored this item. The few participants who reported in CASI experiencing some leakage (n = 7), bloating (N = 3), gassiness (N = 5), or urge to have a bowel movement (N = 5) reported not being bothered much by these side effects. As reported at baseline by the larger cohort before having had any experience with rectal use of the product, participants anticipated less likelihood of using a microbicide for RAI with a lover than with one-night-stands or other partners.

**Table 5:** Product acceptability in Stage 2.

	Total	Tenofovir gel	Placebo gel
	N = 24	N = 12	N = 12
	Mean (SD)	Mean (SD)	Mean (SD)
**Gel’s characteristics**			
Color (N = 17)	9.18 (1.38)	8.78 (1.79)	9.63 (0.52)
Taste (N = 1)	5.00 (0.00)	5.00 (0.00)	5.00 (0.00)
Scent (N = 10)	8.80 (1.62)	9.00 (2.00)	8.67 (1.51)
Consistency	8.21 (1.74)	8.50 (1.57)	7.92 (1.93)
**Application/Applicator**			
Overall application process	8.29 (1.55)	8.25 (1.60)	8.33 (1.56)
Like/dislike of applicator	7.75 (2.97)	8.00 (2.63)	7.50 (3.37)
Ease of portability	7.63 (2.39)	7.92 (2.19)	7.33 (2.64)
**Use**			
Feeling immediately after insertion	8.13 (1.62)	8.17 (1.59)	8.08 (1.73)
Feeling 30 mins after insertion	8.25 (1.98)	8.00 (2.22)	8.50 (1.78)
Bothered by leakage	1.67 (1.71)	1.08 (0.29)	2.25 (2.30)
Bothered by bloating	1.21 (0.66)	1.08 (0.29)	1.33 (0.89)
Bothered by gassiness	1.54 (1.38)	1.33 (0.89)	1.75 (1.77)
Bothered by urge to have BM	1.29 (1.04)	1.58 (1.44)	1.00 (0.00)
**Liking product/likelihood to use**			
Overall liking	7.96 (1.27)	7.58 (1.44)	8.33 (0.99)
Likelihood of using gel for RAI	9.21 (1.10)	9.33 (0.99)	9.08 (1.24)
Likelihood of using gel for RAI with lover	7.83 (2.44)	7.83 (2.52)	7.83 (2.48)
Likelihood of using gel for RAI with ONS	9.33 (1.90)	9.58 (0.90)	9.08 (2.58)
Likelihood of using gel for RAI with other partners	9.58 (0.83)	9.50 (1.00)	9.67 (0.65)
Likelihood of using gel for RAI when no condoms	9.37 (1.93)	9.67 (0.89)	9.08 (2.61)

Comparisons between Tenofovir and HEC placebo gels were all non-significant (P > 0.05). BM; bowel movement, RAI; receptive anal intercourse, ONS; one night stand

Given that in Stage 2 a vaginal applicator was used to deliver TFV and placebo gels rectally, this provided a basis for comparing its acceptability to that of the rectal applicator used in Stage 1B (limited by the fact that the order in which the applicators were used, e.g., rectal first, vaginal second, could not be randomized). Applicator acceptability was rated on a scale of 1–10, with 10 the highest rating. Although participants scoring of applicators indicated high likelihood of using either applicator in the future, many comments were recorded concerning problems with the applicators and suggestions for improvement. Those who tested both applicators liked the vaginal one significantly more than the rectal applicator (7.8 vs 5.2, p = 0.003). Improvements in portability, conspicuousness, aesthetics, tip comfort, product assembly, and packaging were suggested for both [37].

## Discussion

Project Gel demonstrated that it is possible to enroll young high-risk MSM/TGW who are at increased risk for HIV/STI acquisition into a rectal microbicide study. The average age of participants in Stage 1A (23.3 years) contrasts with previous rectal microbicide Phase 1 studies where the average age of study participants was greater than 35 years [40;47]. Enrollment of younger MSM into HIV prevention studies is important as this high-risk group is often underrepresented in HIV prevention trials. However, Newman et al. recently presented data from a discrete choice experiment in which more than 400 young Thai MSM and TGW (Mean age 24.3 years) were asked to provide feedback on rectal microbicide attributes. Anticipated high product use was associated with the following factors: high product efficacy, intermittent use, a gel rather than a suppository, and availability via prescription rather than over-the-counter [48]. In addition other important studies, conducted in Peruvian MSM and TGW, have used conjoint analysis to explore the impact of product and user characteristics on the acceptability of rectal microbicides [49;50].

Traditionally, Phase 1 HIV prevention studies are conducted in low-risk populations. This strategy, although understandable from a product safety perspective, limits the generalizability of the study data. Low-risk participants are likely to view HIV prevention research in a different context to individuals at significant risk of HIV acquisition and may therefore have a different perspective towards product acceptability and desirability. It was noteworthy that Project Gel required that study participants should have a history of condomless RAI. This resulted in recruitment of a population who were at increased risk of HIV infection but who may also have been harder to retain in the study. Study retention varied from 75.4% in Stage 1B to 100% in Stage 2. It is likely that the lower retention in Stage 1B related to the longer duration of this phase of Project Gel but also to the type of participant enrolled in the study. The most common reason for premature discontinuation from Stage 1B was a change in social circumstances such as a new job or the need to move to a new city for employment. Innovative strategies to improve retention of young MSM will be required for the Phase 2/3 evaluation of rectal microbicides where study duration can exceed 18 months.

The prevalence of STIs in Stage 1A (Table 2) was similar or lower than previously published data but this observation is confounded by the fact that many MSM studies have often enrolled older populations attending STI clinics. Project Gel appeared to enroll less risky young MSM since the HIV prevalence in our population was 2.0% which is lower than previous estimates of 4.2% for MSM under 25 years of age [51]. However, the inclusion criteria for Stage 1A stated that participants should be HIV-uninfected or unaware of their status, and this may have selected for a lower prevalence population. Project Gel HIV prevalence data was also much lower than recent estimates for all North American MSM (15.4%) [52]. This is probably an age-related phenomenon, and recent incidence may be a more relevant metric for characterizing the risk profile of this population than prevalence (which includes cumulative incidence). In this regard, two new HIV infections were identified at the end of Stage 1B during which 134 participants were followed for 12 weeks. This approximates to an annual HIV incidence rate of 6.4% and illustrates that the Project Gel participants were indeed high-risk. The prevalence of anal and oral HPV infection was similar to that seen in an Australian cohort of young MSM [53]. The prevalence of HSV-1 and HSV-2 infection was similar to data collected from MSM in the UK [54] and San Francisco [55]. Urethral infections were uncommon compared to other studies in German and South African MSM [56;57]. Rectal GC (1.2%) was also uncommon compared to a large multi-center study in the US where rates were 10.2%. In contrast, rectal CT infection (10.1%) was similar to other published studies [56–58]. Despite counselling and condom provision, Project Gel participants continued to acquire STIs during Stage 1B and Stage 2. These data are concerning as viral and bacterial STIs, particularly anorectal STIs, are a risk factor for the acquisition of HIV infection [59;60].

HRA allows the perianus, anal canal, and distal rectal to be visualized anoscopically by using a light source and magnification. Unlike the rectum, the anal canal is surrounded by the internal and external anal sphincter that makes it liable to mucosal trauma and infection during RAI. The presence of anal findings was less likely to be noted by naked eye exam. This is an important finding for future studies as inflammatory conditions and infections that may facilitate incident HIV infection can be detected by HRA.

Our findings regarding gel and applicator acceptability point to several critical factors for rectal microbicide development. First, gel acceptability and likelihood of use is influenced by several factors, including partner type [44] and product viscosity (Frasca et al, in press). Therefore, a successful microbicide gel would have higher acceptability if positioned as a sexual pleasure enhancer, and it should be available in a range of viscosities. That way, it could be presented to partners as a gel with the qualities they seek to improve sexual satisfaction, and secondarily to prevent HIV. Furthermore, neither the rectal nor the vaginal applicator was ideal as a gel delivery system [37]. Our findings show that applicator satisfaction may be a salient factor in decision-making to use a rectal microbicide in the future (Bauermeister et al, in press). Therefore, further attention is needed to develop user-friendly, quick-acting rectal microbicide delivery systems [37].

Overall adherence to gel use with RAI in Stage 1B was high. Adherence data are based primarily on self-report and may be biased due to recall, missed reporting, or social desirability bias. However, Project Gel integrated multiple modalities of self-report including computer assisted self-interview and interactive voice response systems that may have reduced this potential problem [42]. The main reason for not using the gel was not having it available when needed. Improvements in applicator portability may contribute to increased microbicide use. Adherence measures in this trial, including self-report via CASI and IVRS and product count, were compared to assess convergence. Although self-reports may be subject to social desirability and produce inflated results, we observed convergence among results obtained with different methods. In the absence of a gold standard to measure adherence, these findings continue to support the use of data convergence as a method for adherence measurement in future rectal microbicide trials.

Finally, few participants were aware of PEP and PrEP as prevention options at the start of the study, but their interest was high after learning of these options. Increased public education is needed to raise awareness of PEP and PrEP as HIV prevention methods among MSM and TGW with potential risk behavior; given that they would be likely to use these methods once they become aware of them [43].

In this study we were able to enroll a sexually active population of young MSM/TGW who were willing to use placebo and antiretroviral rectal microbicides. TFV gel was safe and acceptable and should be further developed as an alternative HIV prevention intervention for this population.

## Supporting Information

S1 File Project Gel CONSORT checklist. (DOC)

S2 File Project Gel Trial protocol. (PDF)

S1 Table Summary of visits. (DOCX)

S2 Table Detailed summary of adverse events. (DOCX)
