# Supplementary material for: Project Gel a Randomized Rectal Microbicide Safety and Acceptability Study in Young Men and Transgender Women
Source: PLoS One. 2016 Jun 30;11(6):e0158310. doi: 10.1371/journal.pone.0158310 (PMC4928823; doi:10.1371/journal.pone.0158310)
Supplement: S2 File — (PDF) [file pone.0158310.s002.pdf]

Microbicide Safety and Acceptability in Young Men

Funded by:
US National Institutes of Health
NICHD and NIMH

Grant #: 1R01HD059533-01A1

IND Holder:
CONRAD

This protocol will be performed under CONRAD IND#: 73,382

Principal Investigators:
Ian McGowan, MD, PhD, FRCP
Alex Carballo-Diéguez, PhD

Co-Investigators
Ross Cranston, MD, FRCP
Irma Febo, MD
Kenneth Mayer, MD

Version 3.0

13 November 2012

Microbicide Safety and Acceptability in Young Men

Summary of Significant Protocol Changes			
**V**	**Effective Date**	**Supersedes**	**Change**
1.3	11 May 2010	NA	Initial submission to Puerto Rico site IRB
1.4	06 Jul 2010	11 May 2010	• Initial submission to Boston and Pittsburgh sites<br>• Insertion of oral HPV testing at Visits 1 and 4<br>• Elimination of plasma archive sample at all study visits<br>• Correction of data analysis section, eliminating references to treatment arms and changing the reporting timeframe for AI from two to three months<br>• Update of sample ICFs to include the changes listed above, incorporate standard language from study sites, include reference to Part 3 (upcoming Stage 2 protocol), simplify language for better comprehension at a lower reading level, and eliminate ICF for sample storage
2.0	08 Nov 2010	06 Jul 2010	**MAJOR AMENDMENT**<br>• Added Stage 2, a Phase 1 Rectal Safety Study of 1% Vaginal Tenofovir Gel<br>• Changed Protocol Title to "Microbicide Safety and Acceptability in Young Men" to reflect inclusion of Phase 1 Trial<br>• <u>Updated ALL sections</u> to include Stage 2 and FDA requirements for IND trials (e.g., AE Reporting and Recordkeeping)<br>• Added Hillier Lab, Quest Diagnostics, and Genova Diagnostics to list of Research Laboratories<br>• Increased requirements for recording concomitant medications<br>• Added prohibited medications for Stage 2<br>• Insertion of PT/INR at Visit 4<br>• Inserted language regarding specimen storage<br>• Increased safety reporting requirements and inserted language regarding management of specific adverse events (e.g., hemorrhage, infection, perforation)<br>• Updated Publication Policy per NICHD and CONRAD requests<br>• Included additional appendices for Stage 2 (e.g., Histopathology scoring system and consents) and updated Appendix I Schedule of Study Visits to include Stage 2<br>• Updated Stage 1A and Stage 1B consents to include IRB requested language

3.0	13 Nov 2012	08 Nov 2010	**MAJOR AMENDMENT**<br>• Incorporated changes outlined in Letter of Amendments 1-6<br>• Added a cohort of sex workers to Stages 1A and 1B

Microbicide Safety and Acceptability in Young Men

TABLE OF CONTENTS

LIST OF ABBREVIATIONS AND ACRONYMS....................i

PROTOCOL TEAM ROSTER....................iv

PROTOCOL TEAM ROSTER....................v

PROTOCOL SUMMARY....................viii

1 KEY ROLES....................1
1.1. Protocol Identification....................1
1.2. Sponsor Identification....................1
1.3. Medical Officer....................1
1.4. Co-Investigators....................1
1.5. Research Laboratories....................2
1.6. Data Center UCLA Computing Technologies Research Lab....................2

2 INTRODUCTION....................3
2.1. Background of HIV Prevention and the Epidemic in US Ethnic Minority....................3
2.2. Background of Microbicide Research....................3
2.3. Background of Assessments....................4
2.3.1 Assessment of Anorectal Pathologies....................4
2.3.2 Acceptability of Rectally-Administered Microbicides....................4
2.3.3 Adherence of Rectally-Administered Microbicides....................6
2.3.4 Assessing Acceptability and Adherence of Rectally-Administered Microbicides 6
2.3.5 Assessment of Feasibility of Recruiting and Retaining Sex Workers....................9
2.3.6 Assessment of Rectal Mucosal Damage....................9
2.4. Description of Study Products....................11
2.4.1 Rectal Applicator....................11
2.4.2 Vaginal Applicator....................12
2.4.3 HEC Placebo Gel (Stage 1B and Stage 2)....................12
2.4.3.1 *In vitro* and *Ex vivo* Studies....................12
2.4.3.2 Animal Studies....................13
2.4.3.3 Human Studies....................15
2.4.4 Tenofovir 1% Gel (Stage 2)....................15
2.4.4.1 *In vitro* and *Ex vivo* Studies....................15
2.4.4.2 Animal Studies....................18
2.4.4.3 Human Studies....................27
2.4.5 Justification of Dosing....................30

3 STUDY HYPOTHESES....................31
3.1. Stage 1A....................31
3.2. Stage 1B....................31
3.3. Stage 2....................31

4 OBJECTIVES....................31
4.1. Clinical Objectives:....................31
4.2. Behavioral Objectives:....................32

5 STUDY DESIGN....................32

5.1. Identification of Study Design 32
5.2. Summary of Major Endpoints 33
5.2.1 Clinical Endpoints: 33
5.2.2 Behavioral Outcomes: 33
5.3. Description of Study Population 34
5.4. Time to Complete Accrual 34
5.5. Study Groups 34
5.6. Expected Duration of Participation 34

6 STUDY POPULATION 35
6.1. Selection of Study Population 35
6.1.1 Recruitment 35
6.1.2 Retention 35
6.2. Inclusion Criteria 35
6.3. Exclusion Criteria 36

7 STUDY PRODUCT 38
7.1. Regimen 38
7.2. Administration 39
7.3. Study Product Formulation 39
7.3.1 HEC Universal Placebo Gel for Stage 1B and Stage 2 39
7.3.2 Tenofovir 1% Gel for Stage 2 39
7.3.3 Comparison of HEC Placebo Gel and Tenofovir 1% Gel 40
7.4. Study Product Supply and Accountability 40
7.4.1 Study Product Supply 40
7.4.2 Study Product Receipt 41
7.4.3 Storage 41
7.4.4 Dispensing 41
7.4.5 Accountability 42
7.4.6 Retrieval of Used and Unused Study Products 42
7.4.7 Assessment of Retrieved Study Products and Participant Adherence 42
7.5. Concomitant Medications and Procedures 43
7.6. Prohibited Medications and Procedures 43
7.7. Required Medications and Procedures 44

8 STUDY PROCEDURES 44
8.1. Stage 1A 45
8.1.1 Screening/Enrollment Visit (Visit 1) 45
8.2. Stage 1B 46
8.2.1 Screening/Enrollment Visit (Visit 2) 46
8.2.2 Six-week outpatient use of HEC placebo gel 48
8.2.3 Mid-Trial Follow-up (Visit 3) 49
8.2.4 Six-week outpatient use of HEC placebo gel 49
8.2.5 Final Follow-Up (Visit 4) 50
8.3. Stage 2 50
8.3.1 Screening (Visit 5) 50
8.3.2 Enrollment (Visit 6) 51
8.3.3 Treatment 1 (Visit 7) 53
8.3.4 Treatment 1 Follow-Up Phone Call (Visit 8) 54
8.3.5 Treatment 2 Safety Clearance (Visit 9) 55
8.3.6 One-Week Outpatient Use of Study Product 55
8.3.7 Treatment 2 Follow-up/Final Clinic Visit (Visit 10) 56

8.3.8 Final Follow-Up Phone Call (Visit 11)....57
8.4. Follow-up Procedures for Participants Who Discontinue Study Product 57
8.5. Participants Who Become Infected with HIV....57
8.6. Participants Who Voluntarily Discontinue Study Product 57
8.7. Participants Who Are Discontinued from Study Product by the Site Investigator .58
8.8. Participants Who Withdraw or Are Withdrawn from the Study....58
8.9. Interim Contacts and Visits....60
8.10. Final Contact....60
8.11. Clinical Evaluations and Procedures....60
8.12. Behavioral Measures....62
8.12.1 Stage 1AB....62
8.13. Laboratory Evaluations....66
8.13.1 Local Laboratory Testing....66
8.13.2 Research Laboratory Testing....66
8.13.3 Specimen Collection and Processing....67
8.13.4 Specimen Handling....67
8.13.5 Storage of Specimens for Future Use....67
8.13.6 Biohazard Containment....67

9 ASSESSMENT OF SAFETY....67
9.1. Safety Monitoring....67
9.2. Clinical Data Safety Review....68
9.3. Adverse Events Definitions and Reporting Requirements....68
9.3.1 Adverse Events....68
9.3.2 Adverse Event Relationship to Study Product....69
9.3.3 Serious Adverse Events....69
9.3.4 Serious Adverse Event Reporting Requirements....70
9.3.5 Social Harms Reporting....71

10 CLINICAL MANAGEMENT....71
10.1. Grading System....71
10.2. Dose Modification....71
10.3. Discontinuation of Study Product in the Presence of Toxicity....72
10.4. General Criteria for Discontinuation of Study Product....72
10.5. Management of Specific Adverse Events....72
10.5.1 Hemorrhage Following Rectal Mucosal Biopsy....72
10.5.2 Infection Following Rectal Mucosal Biopsy....72
10.5.3 Perforation of Rectum Following Rectal Mucosal Biopsy....72
10.6. Criteria for Early Termination of Study Participation....73

11 STATISTICAL CONSIDERATIONS....73
11.1. Overview and Summary of Design....73
11.2. Study Endpoints....74
11.2.1 Clinical Endpoints....74
11.2.2 Behavioral Outcomes....74
11.3. Accrual, Sample Size, Randomization, and Blinding....75
11.4. Data Analysis....78
11.4.1 Primary Analysis of prevalence of anal and rectal pathologies and STIs that may facilitate HIV infection....78
11.4.2 Primary and Secondary Analysis prevalence of behavioral practices associated with anal intercourse that may detract from microbicide effectiveness....78

11.4.3 Primary and Secondary Analysis on acceptability of and use-adherence to study gel and applicators 79
11.4.4 Primary and Secondary Analysis on Safety of Tenofovir 1% gel and HEC Placebo gel 81
11.4.5 Secondary Analysis on the Mucosal Damage by Tenofovir 1% gel 81
11.4.6 Analysis of Specific Mucosal Parameters 82
11.4.7 Exploratory Analyses of Mucosal Endpoints and Correlation Between Histological Abnormality and Changes in Mucosal Biomarkers 82
11.4.8 Tertiary Analysis of Feasibility of Recruitment and Retention of Sex Workers and their Likelihood of Use of Non-Condom Based HIV Prevention Strategies 83

12 DATA HANDLING AND RECORDKEEPING 83
12.1. Data Management Responsibilities 83
12.2. Source Documents and Access to Source Data/Documents 84
12.3. Quality Control and Quality Assurance 84
12.4. Study Activation 84
12.5. Study Coordination 84

13 COMPLIANCE WITH APPLICABLE LAWS 85

14 CLINICAL SITE MONITORING 85

15 HUMAN SUBJECTS PROTECTIONS 86
15.1. Institutional Review Boards 86
15.2. Risk Benefit Statement 86
15.2.1 Risks 86
15.2.2 Benefits 89
15.3. Informed Consent Process 89
15.4. Participant Confidentiality 90
15.5. Special Populations 90
15.5.1 Children 91
15.6. Compensation 91
15.7. Communicable Disease Reporting 91
15.8. Access to HIV-related Care 91
15.8.1 HIV Counseling and Testing 91
15.8.2 Care for Participants Identified as HIV-Infected 91
15.9. Study Discontinuation 91

16 PUBLICATION POLICY 92

17 LIST OF APPENDICES 92
APPENDIX I: SCHEDULE OF STUDY VISITS AND EVALUATIONS 92
APPENDIX II: TOXICITY TABLES 92
APPENDIX III: HISTOPATHOLOGY SCORING SYSTEM 92
APPENDIX IV: SAMPLE INFORMED CONSENT FORM (STAGE 1A) 92
APPENDIX V: SAMPLE INFORMED CONSENT FORM (STAGE 1B) 92
APPENDIX VI: SAMPLE INFORMED CONSENT FORM (STAGE 2) 92
APPENDIX VII: SAMPLE INFORMED CONSENT FORM (SAMPLE STORAGE) 92

18 REFERENCES 93

Microbicide Safety and Acceptability in Young Men

LIST OF ABBREVIATIONS AND ACRONYMS

AE	adverse event
AIDS	Acquired Immunodeficiency Syndrome
ALT	alanine transaminase
ARV	antiretroviral
AST	aspartate aminotransferase
ATN	AIDS Trial Network
BBQ	Baseline Behavioral Questionnaire
BUN	blood urea nitrogen
CASI	computer assisted self-interview
CBC	complete blood count
CDC	Centers for Disease Control and Prevention
CFR	US Code of Federal Regulations
cGMP	current good manufacturing practices
CI	confidence interval
CRF	case report form
CRS	Clinical Research Site
CT	*Chlamydia trachomatis,* Chlamydia
CTRL	Computing Technologies Research Laboratory
DAIDS	Division of AIDS
DNA PCR	deoxyribonucleic acid polymerase chain reaction
EIA	enzyme immunoassay
FDA	US Food and Drug Administration
g	gram
GC	*Neisseria gonorrhoeae,* gonorrhea
GCE	growth curve estimation
GCP	good clinical practice
GEE	generalized estimation equations
GLM	generalized linear models
GLP	good laboratory practices
HBsAg	Hepatitis B surface antigen
HEC	hydroxyethyl cellulose
HIV	Human Immunodeficiency Virus
HPV	Human Papilloma Virus
hr	hour
HRA	high resolution anoscopy
HSV-1	Herpes simplex virus type 1
HSV-2	Herpes simplex virus type 2
IATA	International Air Transport Association
ICH	International Conference on Harmonization
IFA	immunofluorescent antibody
IL	interleukin
IoR	Investigator of Record

IRB	Institutional Review Board
IV	intravenous
kg	kilogram
LDMS	Laboratory Data Management System
MDD	microbicide delivery device
MDP	Microbicide Development Program
MIP	macrophage inflammatory protein
mL	milliliter
MMC	mucosal mononuclear cells
mRNA	messenger ribonucleic acid
MSM	men who have sex with men
MTN	Microbicide Trials Network
N-9	Nonoxynol-9
NAAT	nucleic acid amplification testing
NE	neutrophil elastase
ng	nanogram
NICHD	National Institute of Child Health and Human Development
NIH	National Institutes of Health
NNRTI	non-nucleoside reverse transcriptase inhibitor
NIMH	National Institute of Mental Health
NRTI	nucleoside reverse transcriptase inhibitor
NSAID	non-steroidal anti-inflammatory drugs
NVP	nevirapine
OHRP	Office for Human Research Protections
PAQ	Product Acceptability Questionnaire
PBMC	Peripheral Blood Mononuclear Cell
PBS	phosphate buffered saline
PCR	polymerase chain reaction
PEP	post-exposure prophylaxis
PK	pharmacokinetics
PMPA	9-[(R)-2-(phosphonomethoxy) propyl] adenine monohydrate
PMPAp	PMPA monophosphate
PMPApp	PMPA diphosphate
PoR	Pharmacist of Record
PrEP	pre-exposure prophylaxis
PRS	phone reporting system
PTID	Participant Identification Number
qRT-PCR	quantitative real time reverse transcriptase polymerase chain reaction
RAI	receptive anal intercourse
RANTES	Regulated upon Activation-Normal T cell Expressed and Secreted
RNA	ribonucleic acid
RPR	rapid plasma reagin
RT	reverse transcriptase
RT-PCR	reverse transcriptase polymerase chain reaction

SAE	serious adverse event
SBQ	Study Burden Questionnaire
SDF	stromal-derived factor
SDMC	Statistical and Data Management Center
SHIV	Simian/Human Immunodeficiency Virus
SIV	Simian Immunodeficiency Virus
SLPI	secretory leukocyte protease inhibitor
SOP	standard operating procedure
STI	sexually transmitted infection
TERIS	Teratogen Information System
Th	T helper
UAI	unprotected anal intercourse
UCLA	University of California at Los Angeles
ULN	upper limit of normal
VTC	video teleconference
µCi	microcurie
µL	microliter
µM	micromole
WB	western blot

Microbicide Safety and Acceptability in Young Men

PROTOCOL TEAM ROSTER

Jose A. Bauermeister, MPH, PhD
Statistician (Behavioral)
University of Michigan
School of Public Health
1415 Washington Heights, 3822 SPH I
Ann Arbor, Michigan 48109 USA
Phone: 734-615-8414
Fax: 734-763-7379
Email: jbauerme@umich.edu

Alex Carballo-Diéguez, PhD
Co-Principal Investigator
HIV Center for Clinical and Behavioral Studies
New York State Psychiatric Institute and
Columbia University
1051 Riverside Drive, Unit 15
New York, NY 10032 USA
Phone: 212-543-5261
Fax: 212-543-6003
Email: ac72@columbia.edu

Ross D. Cranston MD, FRCP
Co-Investigator
Division of Infectious Disease
University of Pittsburgh Medical Center
3520 Fifth Avenue
Keystone Building, Suite 510
Pittsburgh, PA 15213 USA
Phone: 412-383-2054
Fax: 412-383-2900
Email: rdc27@pitt.edu

Robert Dennis, PhD
Data Center, Director
UCLA Computing Technologies Research Lab
62-073 CHS, 10833 Le Conte Ave
Los Angeles, CA 90095 USA
Phone: 310-206-6556
Email: buddy@ucla.edu

Irma Febo, MD
Co-Investigator
University of Puerto Rico Medical
Sciences Campus
Department of Pediatrics
8th Floor, Room 814, PO Box 365067
San Juan, Puerto Rico 00936 USA
Phone: 787-759-9595
Email: Irma.febo2@upr.edu

Gustavo Doncel, MD, PhD
Scientific and Executive Director
CONRAD
1911 North Fort Myer Drive, Suite 900
Arlington, VA 22209 USA
Phone: 703-524-4744
Fax: 703-524-4770
Email: doncelgf@evms.edu

Rebecca Giguere, MPH
Research Project Manager
HIV Center for Clinical and Behavioral
Studies
New York State Psychiatric Institute and
Columbia University
1051 Riverside Drive, Unit 15
New York, NY 10032 USA
Phone: 212-543-5829
Fax: 212-543-5385
Email: giguere@nyspi.columbia.edu

Laura Janocko
MWRI McGowan Lab Manager
Magee Womens Research Institute
204 Craft Ave Room A540
Pittsburgh, PA 15213
Phone: 412-641-7547
E-mail: janockole@upmc.edu

Microbicide Safety and Acceptability in Young Men

PROTOCOL TEAM ROSTER

Cindy Jacobson, PharmD
Director, Pharmacy Affairs
204 Craft Avenue
Pittsburgh, PA 15213
Phone: (412) 641-8913
Email: rosecj@mwri.magee.edu

Khaleel Khaja
Project Manager
Magee Women's Research Institute
204 Craft Avenue Room A540
Pittsburgh PA 15213 USA
Phone: 412-641-7548
Email: khajak@upmc.edu

Cheng-Shiun Leu, PhD
Statistician (Behavioral)
HIV Center for Clinical and Behavioral Studies
New York State Psychiatric Institute and Columbia University
1051 Riverside Drive, Unit 15
New York, NY 10032 USA
Phone: 212-543-6622
Fax: 212-923-7669
Email: CL94@columbia.edu

Christine Mauck, MD, MPH
Consultant
CKM Consulting, LLC
5 Hidden Ponds Ct.
Gaithersburg, MD 20878 USA
Phone: 301-325-5439
Email: cmauck1@gmail.com

Kenneth Mayer, MD
Co-Investigator
The Fenway Institute
Fenway Community Health
7 Haviland Street
Boston, MA 02115 USA
Phone: 617-927-6400
Fax: 617-267-0764
Email: Kenneth_Mayer@brown.edu

Ian McGowan, MD, PhD, FRCP
Co-Principal Investigator
204 Craft Avenue
Pittsburgh, PA 15213 USA
Phone: 412-641-8999
Fax: 412-641-6170
Email: imcgowan@pitt.edu

Janet Schafer
Manager, Clinical Trials/ Regulatory Affairs
CONRAD
1911 Fort Myer Drive
Suite 900
Arlington, VA 22209 USA
Phone: 703-276-4034
Fax: 703-524-4770
Email: jschafer@conrad.org

Jill Schwartz, MD
Medical Director
CONRAD
1911 Fort Myer Drive
Suite 900
Arlington, VA 22209 USA
Phone: 703-276-3913
Fax: 703-524-4770
Email: jschwartz@conrad.org

Microbicide Safety and Acceptability in Young Men

PROTOCOL TEAM ROSTER

Jonathan Yabes, PhD
Statistician
Center for Research on Health Care Data
University of Pittsburgh School of Medicine
200 Meyran Ave., Suite 300
Pittsburgh, PA 15213
Phone: (412) 605-0158
Fax: 412-586-9672
Email: yabesjg@upmc.edu

Microbicide Safety and Acceptability in Young Men

INVESTIGATOR SIGNATURE FORM

Version 3.0
13 November 2012

Funded by:
US National Institutes of Health
NICHD and NIMH

IND Holder:
CONRAD

I, the Investigator of Record, agree to conduct this study in full accordance with the provisions of this protocol and U.S., state, and local regulations. Whenever the regulations differ between authorities, the more restrictive regulation will apply. I will comply with all requirements regarding the obligations of investigators as outlined in the Statement of Investigator (Form FDA 1572), which I have also signed. I agree to maintain all study documentation for at least two years following the date of marketing approval for the study product for the indication in which it was studied. If no marketing application is filed, or if the application is not approved, the records will be retained for two years after the investigation is discontinued and the US Food and Drug Administration is notified.

I have read and understand the information in the Investigator's Brochure(s), including the potential risks and side effects of the products under investigation, and will ensure that all associates, colleagues, and employees assisting in the conduct of the study are informed about the obligations incurred by their contribution to the study.

____
Name of Investigator of Record

_______________________________ _______________________________
Signature of Investigator of Record Date

Microbicide Safety and Acceptability in Young Men

PROTOCOL SUMMARY

**Short Title:**	Project GEL
Clinical Phase:	1
IND Sponsor:	CONRAD
**Principal Investigators:**	Ian McGowan, MD, PhD, FRCP<br>Alex Carballo-Diéguez, PhD
**Co-Investigators:**	Ross Cranston, MD, FRCP; Irma Febo, MD; Kenneth Mayer, MD
**Sample Size:**	Approximately 280
**Study Population:**	Ethnically diverse (African American, Latino, White, and other), 18-30 year-old HIV-uninfected male adults reporting unprotected receptive anal intercourse (RAI) recruited at three sites, including a subset of sex workers who will be enrolled in Stages 1A and 1B
**Study Design:**	A two-stage longitudinal study including: A clinical and behavioral evaluation (Stage 1A) with an acceptability and adherence trial (Stage 1B), followed by a Phase 1 randomized, double-blind, multi-site, placebo-controlled trial (Stage 2). Participants who complete Stage 1A are eligible to be selected for enrollment into Stage 1B; a similar transition occurs between Stage 1B and Stage 2**.**

Figure 1. Study Schema

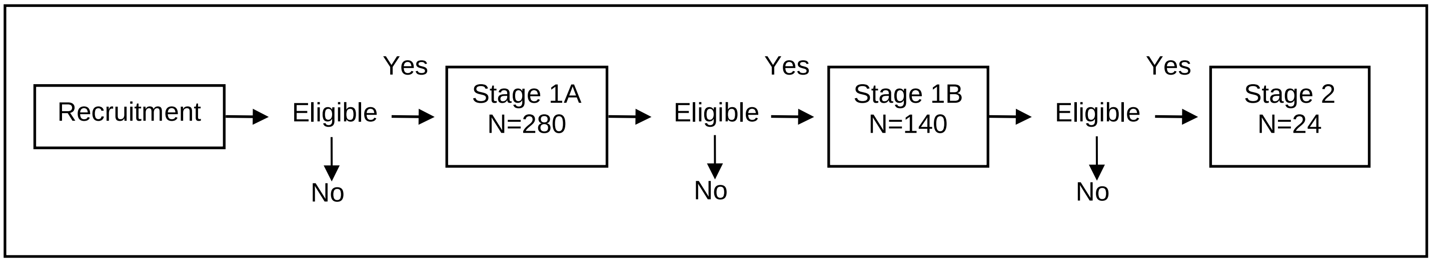

**Study Duration:**	Participant accrual will take approximately 1 year and each participant will be on study for approximately 6 months. The total duration of the study will be approximately 1.5 years.

Study Product: Rectal

- Tenofovir 1% gel
- Hydroxyethyl cellulose (HEC) universal placebo gel

Study Overview:

After completing a screening evaluation, 280 eligible participants, including 40 sex workers, will be enrolled into Stage 1A of the study during which they will undergo a baseline medical evaluation for both history and presence of STIs and anorectal health pathologies or injuries, as well as a detailed Web-based baseline behavioral assessment. The first 140 eligible participants, including 20 sex workers, reporting at least one occasion of unprotected RAI in the previous 3 months will be invited to enroll into Stage 1B. In Stage 1B participants will apply the universal placebo gel (HEC) rectally prior to each episode of RAI over a 3-month period, reporting each use via a phone reporting system; they will complete a Web-based questionnaire and take part in a video teleconference at the end of the 3 months. The first 24 eligible participants completing Stage 1B will be invited to enroll in Stage 2. The subset of sex workers who took part in Stages 1A and 1B will terminate participation at the end of 1B. Eligible participants will be randomized to receive either tenofovir 1% gel or HEC placebo gel as part of Stage 2, the Phase 1 safety study. Following a baseline visit, participants will return to the clinic, where a single dose of the study gel will be administered. Within approximately 30 minutes, rectal swab and rectal biopsy specimens will be obtained via anoscopy. After a one-week recovery period participants will return to the clinic for assessment. If no significant adverse events (AEs) are reported they will begin to self-administer once-daily outpatient doses of the study gel for 7 days, after which they will return to the clinic for evaluation and specimen collection.

Clinical Objectives and Endpoints:

Stage 1AB

- Primary objective: To determine the prevalence of STIs and anal and rectal pathologies that may facilitate HIV infection
- Primary endpoint: Presence of STIs and anal and rectal pathologies as detected by standard anoscopy
- Secondary objective: To determine whether standard anoscopy with examination by the naked eye is as effective in identifying anorectal pathologies as high-resolution anosocopy (HRA)
- Secondary endpoint: Percent of agreement between reports of anal and rectal pathologies by 2 assessment methods (standard anoscopy versus high-resolution anoscopy)

Stage 2

- Primary objective: To evaluate the safety of tenofovir 1% gel when applied rectally
- Primary endpoint: Grade 2 or higher AEs, as defined by the Division of AIDS Table for Grading the Severity of Adult and Pediatric Adverse Events, Version 1.0, Dec 2004 and/or Addenda 3 (Rectal Grading Tables for Use in Microbicide Studies)
- Exploratory objective: To determine whether use of tenofovir 1% gel is associated with rectal mucosal damage
- Exploratory endpoints: Changes in the following parameters:
    - Intestinal histopathology
    - Intestinal mucosal mononuclear cell phenotype
    - Intestinal mucosal cytokine messenger RNA (mRNA)
    - Cytokine profile in rectal secretions

Behavioral Objectives and Outcomes:

Stage 1AB

- Primary objective: To determine the acceptability of and use-adherence to a placebo gel delivered rectally with a rectal delivery device
- Primary outcomes:
    - Identification of factors related to acceptability and adherence
    - Proportion of participants who report via the acceptability questionnaire that they would be very likely to use a similar candidate microbicide gel during receptive anal intercourse (RAI)
    - Proportion of RAI episodes in which the gel was used by participants
    - Comparison between self-reports of gel use and applicator counts
- Secondary objective: To determine the prevalence of behavioral practices associated with anal intercourse that may detract from microbicide effectiveness
- Secondary outcomes:
    - Identification of factors related to sexual behaviors (e.g., douching, lubricant use, recreational drug use, condom use, partner selection)
    - Prevalence of risky sexual practices, douching, lubricant use, recreational drug use, and condom use
- Tertiary objective: To determine the feasibility of recruitment and retention of MSM with high risk sexual behavior, such as sex workers,

for microbicide studies, and their likelihood to use non-condom based HIV prevention strategies

- Tertiary outcomes:
  - Proportion of individuals identifying as sex workers screened to individuals enrolled in the sex worker cohort
  - Proportion of visits completed by participants enrolled in the sex worker cohort
  - Likelihood of using microbicide gels, PrEP, or a rapid HIV Home Test with clients and non-commercial partners for individuals identifying as sex workers

Stage 2

- Primary objective: To determine the acceptability of and use-adherence to a placebo gel or tenofovir 1% gel delivered rectally with a vaginal delivery device
- Primary outcomes:
  - Identification of factors related to acceptability and adherence
  - Proportion of participants who report via the acceptability questionnaire that they would be very likely to use a similar candidate microbicide gel during receptive anal intercourse (RAI)
  - Proportion of RAI episodes in which the gel was used by participants
  - Comparison between self-reports of gel use and applicator counts
- Secondary objective: To determine the prevalence of behavioral practices associated with anal intercourse that may detract from microbicide effectiveness
- Secondary outcomes:
  - Identification of factors related to sexual behaviors (e.g., douching, lubricant use, recreational drug use, condom use, partner selection)
  - Prevalence of risky sexual practices, douching, lubricant use, recreational drug use, and condom use

1 KEY ROLES

1.1. Protocol Identification

Protocol Title: Microbicide Safety and Acceptability in Young Men

Short Title: Project GEL

Date: 13 November 2012

1.2. Sponsor Identification

Funding Agency: National Institutes of Health (NIH), NICHD and NIMH
6100 Executive Blvd.
Bethesda, MD 20892 USA

IND Holder: CONRAD
1911 North Fort Myer Drive, Suite 900
Arlington, VA 22209 USA

1.3. Medical Officer

Bill Kapogiannis, MD
NICHD/Pediatric, Adolescent, and Maternal AIDS (PAMA)
6100 Executive Blvd.
Room 4B11J, MSC 7510
Bethesda, MD 20892-7510
Phone: 301 402-0698
Fax: 301 496-8678
Email: kapogiannisb@mail.nih.gov

1.4. Co-Investigators

Ross Cranston, MD, FRCP
Irma Febo, MD
Kenneth Mayer, MD

1.5. Research Laboratories

Magee-Womens Research Institute
McGowan Laboratory (Room A540)
Hillier Laboratory (Rooms A520, A530)
204 Craft Avenue
Pittsburgh, PA 15213 USA

Quest Diagnostics.
415 Massachusetts Avenue
Cambridge, MA 02139 USA

Genova Diagnostics
63 Zillicoa Street
Asheville, NC 28801 USA

1.6. Data Center

UCLA Computing Technologies Research Lab
62-073 CHS, 10833 Le Conte Ave
Los Angeles, CA 90095 USA

2 INTRODUCTION

2.1. Background of HIV Prevention and the Epidemic in US Ethnic Minority

In the US, it has been documented that young African-American men who have sex with men (MSM) have HIV seroincidence rates similar to, or greater than those found in sub-Saharan Africa[1]. Latino MSM also have disproportionately high prevalence rates of HIV. Furthermore, new infections continue to occur in young white MSM. A large cross-sectional, multi-site, venue-based survey that involved close to 3500 MSM aged 15-22 recruited from 194 public venues showed that HIV prevalence continues to be unacceptably high, with an overall rate of 7.2%, ranging from 2.2% - 12.1% according to location. HIV infection prevalence is higher among blacks (odds ratio [OR], 6.3; 95% confidence interval [CI], 4.1-9.8), young men of mixed or other race (OR, 4.8; 95% CI, 3.0-7.6), and Hispanics (OR, 2.3; 95% CI, 1.5-3.4), compared with whites (comparison group) or Asian Americans and Pacific Islanders (OR, 1.1; 95% CI, 0.5-2.8)[2]. These data demonstrate that behavioral interventions alone have had limited success[3] and consequently attention is currently focused on strategies that utilize both behavior change and biomedical approaches, such as circumcision, antiretroviral pre-exposure prophylaxis (PREP), HIV vaccination, and microbicides[4].

2.2. Background of Microbicide Research

Microbicides are products that can be applied to the vaginal or rectal mucosa with the goal of preventing or significantly reducing the risk of STI transmission including HIV infection[5]. Several human studies have begun to establish the rectal safety of vaginal microbicide candidates (e.g., UC781 and tenofovir) and rectal specific microbicide formulations are currently being studied. The CAPRISA 004 study looked at vaginal use of tenofovir 1% gel among 980 female participants in a pericoital regimen, demonstrating a 39% reduction in HIV acquisition, incidence rate ratio (IRR) = 0.61, 95% CI: 0.4-0.94[6]. However, further research on the safety and effectiveness of tenofovir gel will be required before drug regulatory authorities will consider licensure of tenofovir gel as an approved product for prevention of HIV.

In addition to safety, microbicide safety trials will have to take into account acceptability and adherence. For example, if a product or delivery device has little acceptability among potential users (e.g., if they find it too messy, difficult to administer, or uncomfortable), product adherence (“used as prescribed”) will be low, which may affect interpretation of data from safety trials. Yet, a Phase 1 safety trial has a number of requirements (e.g., daily application of the gel regardless of sexual intercourse) that may affect the acceptability of and adherence to product use. Therefore, it is methodologically challenging to study safety, acceptability, and adherence concurrently. Furthermore, there is a need to identify clinical and behavioral factors that may both contribute to the vulnerability to HIV of young, sexually active MSM and also confound safety trial observations.

This study tackles these problems and public health needs in an innovative fashion. Project GEL will build upon the CAPRISA 004 study to establish the safety of, acceptability of, and use-adherence to tenofovir 1% gel when applied rectally. The study aims are to determine/establish: (1) the prevalence of anal and rectal pathologies and STIs that may facilitate HIV infection; (2) the prevalence of behavioral practices associated with anal intercourse that may detract from microbicide effectiveness; (3) the feasibility of recruiting and retaining high risk MSM such as sex workers; (4) the acceptability of and use-adherence to a placebo gel delivered with a rectal delivery device; (5) the acceptability of and use-adherence to a placebo or tenofovir 1% gel; (6) the safety of tenofovir 1% gel when applied rectally.

2.3. Background of Assessments

2.3.1 Assessment of Anorectal Pathologies

High-resolution anoscopy (HRA) has been used as a tool in the assessment of anorectal health in many studies, including a large anorectal health survey of approximately 800 adults as part of a U-19 program grant on the development of rectal microbicides (PI Peter Anton MD, AI060614). However, HRA requires costly equipment and training to perform adequately. In future rectal microbicide trials HRA equipment may not be available at all sites. This study will evaluate the utility of standard anoscopy as compared to HRA in the assessment of anorectal pathologies. In Stage 1A the Boston and Pittsburgh sites will use HRA as well as standard anoscopy to perform the clinical evaluation, while the Puerto Rico site will use standard anoscopy alone, in the assessment of the initial 240 participants. The 40 participants enrolled in the sex-worker cohort will not have HRA.

2.3.2 Acceptability of Rectally-Administered Microbicides

Microbicides can play an important role in preventing the transmission of HIV only if the target populations find them acceptable and use them correctly and consistently[7-9]. Although there has been some discussion concerning whether acceptability studies should be postponed until efficacy of a product is demonstrated, others[9-11], have convincingly defended the wisdom of integrating acceptability research in early clinical phases of microbicide development. Morrow and Ruiz state that Phase 1 trial participants “are an invaluable source of information regarding acceptability [for] they constitute the handful of individuals with actual product use experience and, thus, are in the best position to provide feedback on actual product characteristics and how these factors may influence individuals’ willingness to initiate and maintain product use over time”[10]. Research on acceptability concurrent with early stages of microbicide development can help scientists choose products that are better suited to the preferences and needs of a target population.[12,13]

A literature search shows that most of the measures available were developed for studies of vaginal microbicide use between men and women with a focus on attitudes about the characteristics of a product (specific colors, smells, tastes, volumes, and consistencies), attitudes about the method of application (the applicator, portability, and

timing), experience with a product before, during, and after sex (leakage, stickiness, wetness or dryness, burning or itching) and its effects on perception of sexual pleasure, and interpersonal issues (ability to use a product without the partner noticing)[1-5, 9, 14-17]. Morrow and Ruiz[10] suggest that Phase 1 and Phase 2 trials include the following factors and variables in the assessments:

- **Vehicle-associated**: Formulation, texture and viscosity, product scent, color, taste, and desirable/appealing elements of product vehicle
- **Application-associated**: Clarity of Instructions, ability to adhere to instructions, ease of product preparation (e.g., filling applicator with product), and ease of application
- **Use-associated**: Frequency and timing of product use, partner-specific, odor post-application and during or after sex, leakage post-application and during or after sex, lubrication and drying effects including post-application and during or after sex, product consistency post-use, desirable/appealing elements of use, use with/without condom, changes in hygiene practices secondary to use, changes in sexual pleasure secondary to use
- **Related Covariates**: History of vaginal and anal product use, frequency of vaginal and anal sex, relationship ‘‘harmony’’; relationship communication

Rosen et al.[18] and Morrow and Ruiz[10] proposed the use of mixed methods (quantitative and qualitative) for the assessment of these factors. This advice is particularly sound in the case of small trials for which the utility of quantitative findings alone often has been limited[19-21]. Also, seminal qualitative work undertaken concerning vaginal microbicides (e.g., Koo et al.[22], Tanner[23], Tolley et al.[11], and Veldhuijdzen et al.[24]) is almost non-existent but very much needed in the area of rectal microbicide acceptability among MSM. This study will make a significant contribution in this respect. Preliminary research findings concerning the potential acceptability of a rectal microbicide have been highly encouraging[25-27]. An NICHD funded trial found that a sexually active cohort of middle aged MSM rated volumes up to 35 mL of gel acceptable for use during anal intercourse[28]. However, information is lacking concerning microbicide acceptability in younger populations, particularly young adult males from ethnic minority groups.

Prevention tools are effective only if used. The limited use of condoms by many at-risk individuals illustrates the importance of a product’s acceptability and perceived need, i.e., the willingness of the users of the product to use it correctly and consistently. This study will explore the acceptability of HEC universal placebo gel for rectal use over a 3-month period by means of a behavioral assessment that includes both structured and semi-structured methods. This assessment will evaluate not only product acceptability, but also the acceptability of the rectal applicator, using “*Tell Alex*”, a personalized research program that combines quantitative and qualitative behavioral data collection methods.

Participant data are collected by a variety of means: 1) Personalized Web-based Computer Assisted Self-Interviews (CASI) (participants respond to *Alex’s* questions), which research has demonstrated result in higher reports of socially censored behaviors than other data collection methods[29]; 2) video teleconferences with *Alex*, which

establish rapport and explore in-depth nuances of product use and negotiations with partners; 3) an innovative Interactive Voice Response phone reporting system (PRS) with *Alex*'s voice, especially designed to appeal to young MSM, which is tied to a weekly or daily monetary incentive so as to encourage consistent reporting; and 4) confidential or anonymous email messages through which participants will be able to keep *Alex* informed of their sexual activities and microbicide use. These tools are tailor-designed to the target population, young ethnically diverse MSM living in urban centers of the US.

The Baseline Behavioral Questionnaire (BBQ) and Product Acceptability Questionnaire (PAQ) were originally developed based on in-depth qualitative interviews of 20 participants in the first phase of R01 HD046060 "Topical Microbicide Acceptability," (Carballo-Diéguez, PI), a study that focused on acceptability of rectal microbicides among men and women.[30, 31] The questionnaires were subsequently administered to more than 100 MSM in Boston and New York, and to 36 men and women participating in a rectal microbicide study in Los Angeles (Peter Anton, PI). This study showed that there were no comprehension problems or other difficulties. There are additional advantages to using the same product-acceptability instrument across studies, since this allows more valid post-hoc comparisons across studies. To complement the BBQ and PAQ, a Study Burden Questionnaire (SBQ) was developed and has been used in a recently completed vaginal study of VivaGel®. The in-depth interviews and emails will provide qualitative data to provide context to the quantitative results obtained from the questionnaires.

2.3.3 Adherence of Rectally-Administered Microbicides

As stated previously, prevention tools are effective only if used. In clinical settings, use of the applicator for product delivery usually occurs in controlled circumstances (e.g., under the supervision of clinic staff). In off-clinic settings, when participants are expected to use a product on their own or prior to intercourse, there is frequently the problem of assessing whether the applicator was used correctly and the product delivered inside the rectal cavity. In general, researchers rely on participants' self reports. However, many factors may make those self-reports unreliable. For example, participants may have poor recall of the number of times they used the applicator and administered the product, particularly if they are required do it repeatedly; participants may also be influenced by social desirability, that is, the wish to be good research participants and fulfill the researchers' expectations; last but not least, in some cases participants may be motivated to enter a trial due to economic or other kind of benefit expected from trial participation, yet be worried about the effect that the product may have in their bodies, and therefore use the product less than the times indicated, if at all. Yet, information is lacking concerning adherence to microbicide use. This study will explore the adherence to product use by means of aforementioned interactive voice response PRS and counts of returned used and unused applicators.

2.3.4 Assessing Acceptability and Adherence of Rectally-Administered Microbicides

To address the need for information concerning microbicide acceptability and adherence

in younger populations, particularly ethnic minority MSM, this study will examine microbicide acceptability and adherence using "*Tell Alex*", a personalized research program that combines quantitative and qualitative behavioral data collection methods. A persona ("*Alex*") is created and incarnated in a young, bilingual (Spanish/English), male Research Assistant located in a different geographical area to that where the study is taking place. This seeks to encourage in the participant the comfort of communicating with a peer plus the reassurance that they are unlikely to run into each other since they live in different communities. The following components make up the behavioral data collection methods:

a. Web-based Behavioral Questionnaires

The Web-based questionnaires consist of the Baseline Behavioral Questionnaire, the Product Acceptability Questionnaire, and the Study Burden Questionnaire. The contents of these questionnaires are briefly described below.

Baseline Behavioral Questionnaire. This questionnaire explores areas likely to facilitate or detract from rectal microbicide use, such as rectal douching, prior experience using rectal lubricants, active pursuit of rectal lubricants with assumed protective qualities, and substance use. Also included in the baseline assessment are questions on sexual behavior in the prior three months for sample description purposes; history of HIV testing behavior; and likelihood of rectal microbicide use in the future.

Product Acceptability Questionnaire. This questionnaire will explore participants' experiences with the gel, including his likes and dislikes concerning the gel, the applicator, the application process, any changes he may have introduced or may wish to introduce in the volume used, any problems (e.g. leakage, soiling) he may have had, sexual enjoyment, partners' reactions, condom use (or lack of use) during receptive anal intercourse using the gel, changes in his habitual behavior, and likelihood of using a microbicide in the future. This last section has items worded similarly to those of the same section administered at baseline in order to compare the anticipated likelihood of product use before and after participants become familiar with the product.

Study Burden Questionnaire. This questionnaire will explore through structured questions the participant's overall experiences during the trial, his likes and dislikes, burden due to study procedures, and suggestions for future trials.

b. In-depth Interviews via Video Teleconference

Video teleconferences will be conducted over the Internet using a webcam. The interview will follow an IRB-approved guide, but will be handled by the interviewers in a flexible manner, following the natural flow of the information presented by the interviewee. New topics are likely to appear in each interview, which may be incorporated into subsequent interviews for further exploration. Two teleconferences are planned:

Pre-trial teleconference. The live meeting via the Internet will serve as an introduction between the interviewer and participant to establish initial rapport. The main purpose of this initial contact is to personalize the experience for the participant and walk participants through all of the steps and procedures for this trial. Participants will receive information about the phone reporting system (PRS) and possibility of sending email messages (both described below), as well as a calendar of events outlining all of the procedures.

End-trial teleconference. Through open-ended questions, the interviewer will explore which aspects of the gel, application method, and applicator the participant liked most and least and which he would change, particularly concerning gel use with RAI. Questions will also be asked to review and explore phone reports. The interview guide will include the following topics:

- Gel use: What it was like for participants to use the gel during the past (three months/week)?
- Problems: Whether participants experienced any problems?
- Rectal Practices: Whether participants used any douches or products rectally?
- RAI: What it was like to have RAI with the gel?
- Partner's reaction: What characterizes the relationship of participant with his sexual partner(s), what level of emotional closeness (or lack of it) exists, and how did these factors influence the use of the gel?
- Likelihood of microbicide use in the future: How likely would participant be to use the gel in the future based on gel use in the study in comparison with other products?
- Besides product use related issues, this study will explore how the participant felt about the research technology used, including computers and Internet, phone reporting system, and teleconferencing.

With the sex worker cohort, the following will also be explored:

- Sex work environment: how using non-condom based HIV-prevention strategies may fit in with sex work
- RAI with clients: What was it like using the placebo gel with clients?
- Likelihood of use of non-condom based HIV prevention strategies: How likely would participant be to use microbicide gel, rapid HIV home test with clients, or PrEP?

c. Interactive Voice Response Phone Reporting System (PRS)

The phone reporting system programmed with *Alex*'s voice is designed to appeal to young MSM. The phone reports will constitute a measurement of adherence to product use. It is tied to a weekly or daily monetary incentive so as to encourage consistent reporting and will focus on the participant's use of the gel, use of other products rectally, whether he had RAI, whether a condom was used, and partners' reactions to the gel.

d. Email Messages

Participants will have the option to send email messages to *Alex* as a complement to the phone reports. Use of an anonymous email account will be possible, if

participants so choose. The addition of email messages to the data collected through the phone reporting system will result in enriched records of participants' experiences with the gel.

2.3.5 Assessment of Feasibility of Recruiting and Retaining Sex Workers

In order to decrease the rate of HIV infection in North America, treatment options and research must target the populations more heavily affected by the HIV epidemic in North America. Hillary Clinton, in her recent address to the delegates of AIDS 2012, pointed out that to combat AIDS the US must focus on the needs of MSM and sex workers given that their rates of HIV are much higher than those of the general population. She emphasized that we cannot afford to avoid sensitive conversations nor fail to reach out to the highest risk, most marginalized groups.

To advance this scientific agenda the feasibility of recruitment and retention of MSM with higher risk sexual behavior for rectal microbicide studies must be established. This study aims to determine whether marginalized individuals who need to resort to transactional sex for survival can be recruited and retained as study participants or not, and whether they are likely to use non-condom based HIV prevention strategies.

In order to assess the feasibility of recruiting this high risk population, this study will enroll a cohort of 40 participants who engage in transactional sex (i.e., exchanging sex for goods, money, or shelter). Recruitment and retention of this additional cohort will provide invaluable lessons on how to develop studies that are culturally sensitive and specifically adapted to the needs of the North American population.

2.3.6 Assessment of Rectal Mucosal Damage

The rectal compartment is highly vulnerable to HIV transmission. A single layer of columnar epithelium separates the intestinal lumen from the lamina propria. The lamina propria is populated with a broad range of HIV target cells including macrophages, dendritic cells, and activated CD4+ T lymphocytes expressing the CCR5 and CXCR4 HIV-1 coreceptors.[32] It is likely that the immune composition of the rectal mucosa is at least partially responsible for the 10- to 20-fold increased risk of HIV transmission associated with anal[33, 34] compared to vaginal intercourse.[35, 36] Any product that induces local inflammation is likely to further increase this risk by recruiting and/or activating the immune target cells.

Methods to assess microbicide induced toxicity in the rectal compartment are in a state of evolution. Mucosal changes may be subtle and require new modes of detection (polymerase chain reaction (PCR), flow cytometry, immunohistochemistry, etc). For this reason, the HIV Prevention Trial Network sponsored the HIV Prevention Trials Network (HPTN) 056 study "Characterization of Baseline Mucosal Indices of Injury and Inflammation in Men For Use in Rectal Microbicide Trials" conducted at UCLA.[14] The

lessons learned from the HPTN 056 trial have guided the selection of parameters to be included in this protocol. As these are assays in development and clinical relevance remains to be defined, these will not be safety indices but exploratory endpoints. The rationale for selecting each of these endpoints is further described below:

Intestinal histopathology

Histopathological assessment of intestinal tissue is a routine method of demonstrating mucosal abnormality associated with gastrointestinal diseases such as ulcerative colitis, Crohn's disease, and gluten enteropathy (celiac disease). In general, mucosal change in these diseases can be quite dramatic whereas microbicide-induced changes may be quite subtle. As a consequence this study will use a qualitative scoring system (See Appendix III) developed by the inflammatory bowel disease community[37] and adapted for use in HPTN 056.[14] Prior to the HPTN 056 study, one rectal microbicide study using histological data,[38] employed a simple scoring system of normal, slightly abnormal, or abnormal. Using this histological system, 69% of the placebo recipients and 89% of the N-9 recipients had slightly abnormal or abnormal rectal biopsies. The scoring system developed for the HPTN 056 study might provide better discrimination between abnormal and normal histology. The HPTN 056 scoring system was further refined and used in an as of yet unpublished rectal safety study of the microbicide UC781. The refined scoring system used in the UC781 study will be used in this study.

Intestinal mucosal mononuclear cell phenotype

Enzymatic digestion of intestinal biopsies and flow cytometric analysis of T Cell populations[39] will be used to determine if product administration is associated with changes in mucosal T cell populations, co-receptor expression, or T cell activation. Co-receptor expression (e.g., CCR5, CXCR4, etc.) on mucosal T cells is important for HIV-1 entry. In healthy HIV-1 seronegative individuals, the expression level of CCR5 is increased seven-fold in mucosal mononuclear cells (MMC) compared to peripheral blood mononuclear cells (PBMC).[32] CXCR4, however, is expressed in CD45RO+ T cells in similar levels as in MMC and PBMC. It has been shown that MMC are more easily infected with HIV-1 than PBMC.[40, 41] Explanations for the high susceptibility of MMC to HIV-1 may include the increased expression of HIV 1 co-receptors, especially CCR5, as well as the heightened activation status of the MMC. The expression of CCR5 has been shown to be up-regulated by pro-inflammatory and T helper (Th)-1 cytokines, while Th-2 cytokines up-regulate CXCR4.[42, 43] This suggests that expression of CCR5 and CXCR4 is partly controlled by Th1/Th2 type of cytokines, which have been shown to be up-regulated in rectal mucosa from HIV-infected patients[14]. It will be important to ascertain whether microbicidal agents trigger similar responses and associated increased vulnerability to HIV infection.

All flow cytometry will be performed at the McGowan Laboratory in Pittsburgh, PA. Samples from the Boston, MA and San Juan, PR sites will be sent by overnight courier to Pittsburgh. This approach has been used before by other investigators[44, 45] and it is anticipated that it will be possible to conduct adequate assessment of mucosal T cell populations on these samples.

Intestinal mucosal cytokine mRNA

Documentation of an increase in mucosal production of pro-inflammatory cytokines such as interleukin (IL)-6 or IL-8 following microbicide exposure may act as a surrogate marker of product induced toxicity.[46] Recent work has helped define the optimal methodology to measure cytokines in biological samples.[47] In this study proinflammatory cytokines that have been associated with increased recruitment of potential HIV target cells and/or replication of HIV infection will be measured. Previous HIV mucosal pathogenesis studies have demonstrated significant increases in mucosal cytokine mRNA in individuals with untreated HIV infection compared to controls or patients with undetectable plasma HIV viremia.[48, 49]

CCL5, also known as Regulated upon Activation-Normal T Cell Expressed and Secreted (RANTES), macrophage inflammatory protein (MIP)-1α and MIP-1β13 are the natural ligands for CCR5 while stromal-derived factor (SDF)-1 is the ligand for CXCR4. The physiological function of β chemokines and their receptors is to direct migration of recruited lymphocyte subsets to sites of inflammation and immune activation furthering the inflammatory cascade. Blocking chemokine activity has proved to be effective for inhibiting the migration of certain leukocytes while up-regulation of chemokine receptors and their ligands are characteristic correlates of mucosal inflammation.[50,51] Immune activation of resting CD4+ T cells has been shown to trigger viral replication and spread.[52,53]

In this study quantitative, real-time reverse transcriptase polymerase chain reaction (qRT-PCR) will be used to quantify mucosal mRNA expression of proinflammatory cytokines, chemokines, and chemokine receptors (e.g., IL-1β, IFN-γ, TNF-α, IL-6, IL-8, IL-12, IL-17, IL-23, MIP-1α, MIP-1β, RANTES, CCR5). The final mucosal mRNA panel will be determined at the time all of the samples are processed to reflect the most current scientific literature.

Cytokine profile in rectal secretions

As discussed above, measurement of cytokines or chemokines in mucosal tissue or local secretions may provide important information regarding the potential for a candidate microbicide to induce mucosal toxicity. In addition to the mRNA analysis of intestinal tissue biopsies, cytokine levels in rectal secretions will be quantified using the Luminex technique, which can measure multiple cytokines or chemokines in small volumes (< 100 μL) of rectal secretions. Luminex will be used to measure cytokines, chemokines, and chemokine receptors (e.g., IL-1β, IFN-γ, TNF-α, IL-6, IL-8, IL-12, IL-17, IL-23, MIP-1α, MIP-1β, RANTES, CCR5). The final Luminex panel will be determined at the time all of the samples are processed to reflect the most current scientific literature.

2.4. Description of Study Products

2.4.1 Rectal Applicator

Prior studies have demonstrated that the applicator used to deliver a rectal gel was a crucial factor influencing rectal microbicide acceptability.[26] Dr. Carballo-Diéguez conducted a study to develop a prototype of an inexpensive standard rectal microbicide

applicator, optimized for ease of use, comfort, and effective delivery of microbicide gel across a wide range of dose volumes to both men and women who have anal intercourse. This applicator, manufactured by HTI Plastics, the company that produces the vaginal applicators used in most vaginal microbicide trials, will be used in Stage 1B of this study. Applicators will be filled with about 6.65 mL of gel which results in the delivery of approximately 4 mL of gel. For the sex worker cohort, the vaginal applicator (see Section 2.4.2) filled with placebo gel will be used in Stage 1B.

2.4.2 Vaginal Applicator

Given that the stability and compatibility of tenofovir gel in the rectal applicator have not been established, the vaginal applicator designed for use in the vaginal microbicide clinical trials of tenofovir, for which the stability and compatibility has been previously established, will be used in Stage 2. This applicator has been extensively used in vaginal trials; this is not a rectal-specific applicator design. The single-use vaginal applicator consists of a barrel and plunger with a screw-on cap. The barrel measures 114 mm long by 12.7 mm wide with a tapered and rounded tip and a flanged base. This applicator, manufactured by HTI Plastics, will be used in Stage 2 of this study. The dose volume of 4 mL used in previous trials of tenofovir 1% gel has been selected.

2.4.3 HEC Placebo Gel (Stage 1B and Stage 2)

2.4.3.1 *In vitro* and *Ex vivo* Studies

Formulation Testing

Analyses of pH (HEC gel mixed with human seminal plasma, 8.03± 0.26) found that a HEC formulation did not show significant buffering capacity and could not acidify the alkaline pH of seminal plasma, a favorable property for a placebo formulation[62]. *In vitro* assessments of spermicidal activity utilizing human semen from healthy donors showed that HEC gel had no significant deleterious effects on sperm motility, even after 60-minute incubation.

Condom Integrity

The effects of HEC-based placebo gel on three brands of condoms including Trojan Enz®, Durex® and Trojan Supra® have been evaluated[63]. The physical properties of each were not significantly affected. Although there were slight increases in airburst volume for all types, and an increase in pressure for synthetic condoms following gel exposure, this was considered normal and not statistically significant.

Safety Testing in Cell Lines

Dilutions of the HEC gel in culture medium exhibited negligible toxicity to human vaginal epithelial cells (standard MTT assay), even at the lowest dilution tested (1:2).[63] Exposure of human vaginal epithelial cells to the HEC gel resulted in minimal IL-1α induction, even at the lowest dilutions tested (lowest dilution, 1:2). Additional studies have shown that HEC gel is safe to peripheral blood mononuclear

cells, and colorectal epithelial cell lines.[64, 65] Indeed, no changes in the transepithelial resistance was noted after HEC gel was applied.[65]

Safety Testing in Colorectal Explant Cultures

The HEC gel was applied to colorectal explant tissues using a polarized system.[64] For safety analysis the MTT assay and histology were performed. No observed reduction in the MTT levels or changes in the tissue architecture were noted.

Anti-HIV-1 Activity

Further analysis showed that this gel has no anti-HIV activity as it did not protect peripheral blood mononuclear cells, macrophage, or colorectal explant cultures from infection.[64, 65]

2.4.3.2 Animal Studies

Toxicology

HEC is the thickener in the placebo gel. The results of multiple animal studies have been consistent with the safety of this ingredient.

Up to 55 intravenous injections of HEC were given to dogs (dose and number not specified) without causing injury other than that typical of the other water-soluble cellulose ethers.[66] Only transitory changes in the hematological indices and the deposition of the material on the intima of the blood vessels were noted. Groups of rats maintained for two years on diets containing HEC (n not specified, up to 5%) did not exhibit any adverse effects. HEC has also been administered to rats in single oral doses as high as 23,000 mg/kg without observed toxic effects (n not specified).

Intraperitoneal administration of unformulated HEC to pregnant mice in a 1% and 4% concentration caused an increase in fetal resorptions, but no detectable increase in birth defects.[67] While no epidemiological studies of congenital anomalies in infants born to women exposed to HEC during pregnancy have been reported, the Teratogen Information System (TERIS) considers the magnitude of teratogenic risk to a child born after exposure during gestation to be none.[68]

CF-1 mice (n not specified) pretreated with medroxyprogesterone acetate were administered 0.02 mL of HEC gel vaginally, followed by a 0.01 mL inoculum of 10 intravaginal $dose_{50}$ units of HSV-2 0.3 minutes later.[62] On day 3, vaginal lavage was cultured on human foreskin fibroblasts, and mice were considered infected if a cytopathic effect was observed after 3 days of incubation. Control animals were treated similarly but were not administered the test article. Infection rate following pretreatment with HEC gel (90%) was not significantly different from pretreatment with PBS (80%) or from mice given no treatment (% not specified). HEC gel did not enhance susceptibility of mice to HSV-2 when administered 12 hours before vaginal challenge.[63]

A 10-day rabbit vaginal irritation study (10/arm, 2 arms, HEC gel vs. 0.9% saline control) found that the HEC gel was not irritating to the vaginal mucosa of rabbits when dosed daily for 10 days. One animal in the HEC gel group had an instance of vaginal redness (compared to four animals in the saline group), which did not persist and was not evident at the end of the study. Diarrhea, few feces, and soiling of the anogenital area were noted in that animal. Body weight changes were noted to be normal. In 9 of 10 animals, necropsy results were normal. Anogenital soiling was observed in the animal that exhibited erythema during the in-life phase of the study. Histopathological changes observed were similar to those seen in the control group and likely attributable to those that occur as a result of the repeated insertion of a catheter, rather than due to any effect of the test samples.

A recently completed rectal study in a macaque model also appears to be consistent with the safety of this ingredient. HEC gel was used as the placebo comparator in a rectal safety study of a combination microbicide in a macaque model.[69,70] A third study arm received no product and served as a negative control. Rectal safety of the active product and HEC gel was evaluated following four daily applications of study products. Rectal flora, pH, and rectal lavage samples were assessed pre-and post-dosing and showed no evidence of toxicity in the macaques that received HEC gel. The infrequent evidence of epithelial sloughing and rare incidence of associated blood cells in rectal lavage samples was similar in the HEC placebo and no product arms of this study.

Effectiveness

The effect of the placebo gel on vaginal transmission of $SHIV_{162p3}$ (10^3 $TCID_{50}$) to rhesus monkeys was determined in two separate studies (n = 5, n = 3, respectively).[69] Macaques pretreated with medroxyprogesterone acetate were vaginally administered 1 mL of the HEC gel formulation 15 minutes prior to challenge with 0.5 mL $SHIV_{162p3}$. Investigators monitored total RNA load in the animal plasma for a total of 8 weeks by means of a standard quantitative RT-PCR. The first study utilized the HEC gel formulation at pH 6.5; the second study utilized a formulation at pH 4.4. In both studies, all monkeys were infected, as determined by the presence of viral RNA in circulating blood, regardless of the pH of the formulation.

2.4.3.3 Human Studies

Unformulated hydroxyethylcellulose is known to be a non-irritating substance in humans (skin sensitization is unusual), with doses less than 2 g/kg by ingestion not expected to be toxic[71]. No inhalation studies have been conducted, but exposure of humans to the dust in manufacturing operations over many years has not led to any known adverse effects. The hydroxyethylcellulose placebo formulation was developed and adopted for use in the HPTN 035 microbicide study, the Phase II/IIb Safety and Effectiveness Study of the Vaginal Microbicides Buffer Gel and 0.5% PRO2000/5 Gel (P) for the Prevention of HIV Infection in Women.

A randomized, closed label, Phase I study of daily vaginal HEC placebo exposure was conducted in 2003[72]. In this trial, 30 women were randomized to twice-daily vaginal applications of 3.5 mL of the universal (HEC) placebo or polystyrene sulfonate (PSS) vehicle. The primary objective of this study was to assess and compare the effects of the test articles on symptoms and signs of irritation of the external genitalia, cervix, and vagina as seen on naked eye exam after 7 and 14 days of use including disruption of the epithelium and blood vessels as seen on colposcopy after 14 days of use. Secondary objectives included: an assessment and comparison of differences in vaginal health by evaluating the results of wet mounts, pH, and Gram-stained vaginal smears (Nugent score and neutrophil counts) after 7 and 14 days of use and vaginal cultures after 14 days of use; and an assessment of acceptability of the study products after 14 days of use among participants.

Results of this trial indicated that both gels appear safe for vaginal use twice a day for 14 days in sexually abstinent women. Two out of 14 women (14.3%) randomized to the HEC group reported at least one symptom of mild severity of genital irritation, which included genital burning, soreness and pelvic pain. A lower proportion of women in the HEC group experienced any evidence (signs and/or symptoms) of genital irritation. Three out of 14 women in the HEC group (21.4%) had colposcopic findings that included erythema, petechiae and peeling.[72] No deep genital disruption was observed in either product group. Minimal changes in wet mounts, pH, Nugent scores, neutrophils, and vaginal flora were observed in both product groups.

HEC placebo gel is also being used rectally in several ongoing trials of rectal microbicide candidates

2.4.4 Tenofovir 1% Gel (Stage 2)

2.4.4.1 *In vitro* and *Ex vivo* Studies

Formulation Testing

The original formulation (used in all vaginal microbicide tenofovir gel studies) may have been associated with mild gastrointestinal intolerance in a previous rectal

microbicide study (MTN-006). These adverse events were thought to be potentially linked to the high osmolarity of the vaginal formulation of the tenofovir 1% gel. A reduced-glycerin vaginal formulation (1st RGVF) was developed with the intent of decreasing these adverse events by reducing the glycerin content to decrease the osmolality. A second, newer RGVF (2nd RGVF) has since been developed with a higher propylparaben content to improve stability. In order to maintain a viscosity similar to the original formulation the HEC content of the 2nd RGVF was also increased. The changes to the excipients are minimal. Project GEL will be one of the first clinical trials using the 2nd RGVF gel formulation.

Chemical Name	HEC Placebo % w/w	Original VF % w/w	1st RGVF % w/w	2nd RGVF % w/w
Glycerin	0	20.00	5.00	5.00
Hydroxyethyl cellulose	2.70	2.50	2.75	3.00
Carbopol 974	0	0	0	0
Sodium carboxymethylcellulose	0	0	0	0
Sodium chloride	0.85	0	0	0
Methylparaben	0	0.18	0.22	0.22
Propylparaben	0	0.02	0.024	0.05
Purified water	96.3	75.23	89.936	89.66
Disodium edetate (EDTA)	0	0.05	0.05	0.05
Citric acid	0	1.00	1.00	1.00
Sorbic acid	0.10	0	0	0
PMPA	0	1.00	1.00	1.00
Sodium hydroxide	0.10	As needed	As needed	As needed
Diluted hydrochloric Acid	0	As needed	As needed	As needed
pH	4.4	4.5	4.6	4.5
Osmolality (mmol/kg)	~~400~~ isotonic	3111	836	846

Condom Integrity

The compatibility of original tenofovir 1% vaginal gel was tested with three types of lubricated male latex condoms.[73] A matched placebo gel and placebo gel (HEC gel as planned for this trial) were used as comparator gels. The condoms tested were representatives of leading brands on the US market (Trojan® and Durex®) with either silicone or aqueous lubricant. The airburst test was used to evaluate changes in film integrity (strength) and test specimens were measured before and after treatment with the gels to assess changes in strength properties following the application of the three gel preparations. All three gels were shown to be compatible with the above condoms. The compatibility of tenofovir 1% gel with Alatech™ Healthcare (Eufala, AL) male latex silicone lubricated condoms was also evaluated. Tenofovir placebo gel was used as a comparator. The two application treatments of tenofovir 1% gel and matched placebo gel increased airburst volumes by 5 to 6 L compared with the baseline. With an increase in volumes there was a decrease in airburst pressures by 0.2 kPa. This implies a physical change to a more elastic condom. This slight

change in physical properties suggests an interaction of the tenofovir 1% gel with the silicone lubricant, but does not indicate that the condoms are unsuitable for use in clinical studies. The formulation changes do not invalidate these studies, because there are no new polymers to interact with the condoms. However, additional condom compatibility studies may be performed as the development of the reformulated tenofovir 1% gel moves forward.

Safety Testing in Cell Lines

Safety testing in epithelial cell lines has demonstrated retention of transepithelial resistance (TER) by Caco-2 and HEC-1-A cell lines, unlike the original formulation, which induced a transient drop in the epithelial resistance.[74] These data suggest that the reformulated tenofovir gel is just as effective as the original formulation but is less toxic to the epithelium.

Safety Testing in Colorectal Explant Cultures

Safety testing of colorectal explants shows similar MTT (Formazan [1-(4, 5-dimethylthiazol-2-yl)-3, 5-diphenylformazan]) results with both formulations[74, 75]. However, histological testing showed retention of the epithelium after application of the reformulated tenofovir gel as compared to epithelial stripping with the original formulation. Additional testing in colorectal explant cultures also showed that the new formulation did not compromise product efficacy. Collectively, these data suggest that the reformulated tenofovir gel is just as effective as the original formulation but is less toxic to the epithelium.

Anti-HIV-1 Activity, Resistance, and Cross-resistance

The *in vitro* antiviral activity of unformulated tenofovir against laboratory and clinical isolates of HIV-1 was assessed in lymphoblastoid cell lines, primary monocyte/macrophage cells and peripheral blood lymphocytes.[76, 77] The 50% effective concentration (EC_{50}) values for tenofovir were in the range of 0.04 µM - 8.5 µM. In drug combination studies of tenofovir with NRTIs (abacavir [ABC], didanosine [ddI], lamivudine [3TC], stavudine [d4T], zalcitabine [ddC], zidovudine [ZDV]); non-nucleoside reverse transcriptase inhibitors (NNRTI) (delavirdine [DLV], efavirenz [EFV], nevirapine [NVP]); and protease inhibitors (amprenavir [APV], indinavir [IDV], nelfinavir [NFV], ritonavir [RTV], saquinavir [SQV]), additive/synergistic effects were observed. Tenofovir displayed antiviral activity in vitro against HIV-1 clades A, B, C, D, E, F, G, and O (EC_{50} values 0.5 µM - 2.2 µM) and showed strain specific activity against HIV-2 (EC_{50} values ranged from 1.6 µM to 5.5 µM).

HIV-1 isolates with reduced susceptibility to unformulated tenofovir have been selected in vitro.[77,78] These viruses expressed a K65R mutation in RT and showed a 2-4 fold reduction in susceptibility to tenofovir. Of note, this mutation also confers increased susceptibility to some other nucleoside reverse transcriptase inhibitors (NRTI), and is associated with approximately 50% reduction in the replicative capacity of HIV-1 (potentially resulting in a "less fit" virus).[79] Tenofovir-resistant isolates of HIV-1 have been recovered from some patients treated with Viread® in combination with certain antiretroviral (ARV) agents.[77] In treatment-naïve patients,

8/47 (17%) isolates from patients failing Viread® + 3TC + EFV through week 144 showed >1.4 fold (median 3.7) reduced susceptibility in vitro to tenofovir.

Cross-resistance among certain NRTIs has been recognized.[76,77] The M184V/I and/or K65R substitutions selected in vitro by the combination of emtricitabine (FTC) and unformulated tenofovir are also observed in some HIV-1 isolates from subjects failing treatment with tenofovir in combination with either 3TC or FTC, and either abacavir, didanosine, or zalcitabine. Therefore, cross-resistance among these drugs may occur in patients whose virus harbors either or both of these amino acid substitutions. In treatment-experienced patients, 14/304 (5%) isolates from patients failing Viread® through week 96 showed >1.4 fold (median 2.7) reduced susceptibility to tenofovir. Genotypic analysis of resistant isolates showed a mutation in the HIV-1 RT gene resulting in the K65R amino acid substitution. HIV-1 isolates from patients (n = 20) whose HIV-1 expressed a mean of 3 ZDV-associated RT amino acid substitutions (M41L, D67N, K70R, L210W, T215Y/F, or K219Q/E/N) showed a 3.1-fold decrease in the susceptibility to tenofovir. Multinucleoside resistant HIV-1 with a T69S double insertion mutation in the RT showed reduced susceptibility to tenofovir.[78]

2.4.4.2 Animal Studies

The formulation changes to the tenofovir 1% gel were minor (e.g., lowering the glycerin content). Therefore, the existing data from animal studies with the old formulation are valid for the new formulation. Repeating the animal studies with the new formulation was deemed unnecessary.

Toxicology

Toxicology studies have been performed for both vaginal and rectal administration of tenofovir gel.[73, 80]

14-Day Vaginal Irritation and Toxicity Study of Tenofovir Gel in Rats

Ten female Sprague Dawley rats/group received either 0% (vehicle control), 1%, 3%, or 10% tenofovir gel (2.5% HEC formulation) by intravaginal administration (0.5 mL/dose) once daily for 14 days.[73, 80] There were no mortalities, and no tenofovir-related clinical signs of toxicity or changes in body weight, food consumption, or absolute/relative kidney weights. Individual and mean vaginal (gross) irritation scores for all tenofovir-dosed animals sacrificed at Day 15 were graded as 0 (no erythema or edema); microscopic irritation scores for the vagina, cervix, ovaries, uterine horns, and vulva were graded as 0 (normal histology). No tenofovir-related histopathological effects on the vagina, cervix, ovaries, uterine horns, vulva, or kidneys were observed.

10-Day Vaginal Irritation Study of Tenofovir Gel in Rabbits

The potential irritant effects of tenofovir were evaluated in vaginal tissues of female New Zealand White rabbits using three different gel formulations (2.5% HEC or 1.0

to 2.0% Carbopol® 1342).[73] This study consisted of eleven treatment groups (five rabbits/group) that received one of the following: a sham treatment or Conceptrol® (positive control); 0%, 0.3%, 1.0%, 3.0%, or 10.0% tenofovir formulated in the HEC gel preparation; or 0% or 3.0% tenofovir formulated in a 1.0% or 2.0% Carbopol® 1342 gel preparation. With the exception of the sham dose group, all rabbits received dose formulation (1.0 mL/dose) daily applied topically to the mucosal surface of the vaginal vault for 10 consecutive days. No mortalities and no tenofovir-related clinical signs of toxicity or body weight changes were observed in this study. Group composite vaginal irritation scores for the 10% tenofovir topical gel (HEC formulation), 0% tenofovir (1.0% Carbopol® 1342 formulation), and Conceptrol® (positive control) dose groups were each rated as "mild." Composite vaginal irritation scores rated "minimal" were observed for all other tenofovir, vehicle or sham treatment groups, regardless of the formulation. No unacceptable level of mucosal irritation was observed in any treatment group based on the protocol-derived criteria for this animal model. Generalized erosion and/or ulceration were observed only in animals receiving Conceptrol® positive control (two of five) or the 10% tenofovir topical gel (two of five).

<u>14-Day Rectal Irritation Study of Tenofovir Vaginal Gel in Rabbits</u>

Forty New Zealand White rabbits (approximately 10-12 weeks of age and weighing in the range of 2.0 to 2.5 kg at initiation of treatment) were assigned to five dose groups (one sham control, one placebo control and three active test article) consisting of four animals per sex per group under Good Laboratory Practices [(GLP) Pacific BioLabs, Hercules, CA].[73] The placebo control and active test articles consisted of tenofovir matched placebo gel and three different concentrations (1%, 3%, and 10%) of tenofovir gel respectively. The lubricant for the sham control group was K-Y Jelly from a commercial source.

All female animals were dosed for 14 days and all male animals were dosed for 15 days. Animals in Groups 2 to 5 received 1 mL doses of the respective placebo or test articles via rectal administration for 14/15 consecutive days. A short, soft catheter was attached to a syringe and filled with 1 mL of the appropriate test article. Animals in Group 1, (sham control) underwent the same treatment procedure for 14/15 days with the exception that no dose was administered and the catheter was lubricated with a non-irritating lubricant (K-Y Jelly) prior to insertion. The rectal route of administration was selected as it is the intended clinical route of administration.

Table 1: Rectal Irritation Study in New Zealand White Rabbits

Group	Intervention
1	sham
2	tenofovir placebo gel
3	tenofovir 1% gel
4	tenofovir 3% gel
5	tenofovir 10% gel

The test article, vaginally formulated tenofovir 1% gel, was well tolerated at dose concentrations (1 mL dose volume) of 1% (10 mg/dose), 3% (30 mg per dose) and 10% (100 mg per dose) when administered as a daily rectal dose for 14 days to female rabbits or 15 days to male rabbits. There was no mortality in this study, and there was only one clinical finding that was potentially study-related: redness at the site of administration in one animal on one day of dosing. There was no evidence of a test article effect on body weight, body weight gain or food consumption over the dose period.

The test article, at the concentrations tested, was without significant effect at the rectal site of administration. Gross pathology at necropsy provided no evidence for tissue damage or inflammation of the rectum or surrounding tissues at the concentrations tested; histopathological evaluation of the rectum and parts of the colon immediately adjacent to the rectum also showed no effect at the concentrations tested. Each rectum sample was subsectioned into proximal, mid and distal sections (in relation to the site of test article application) for histopathological analysis. Within each section, at least 5 subsections were evaluated for inflammation and other types of lesions. As mentioned, no differences were seen.

Rectal administration of the test articles produced little evidence of test article related systemic effects, despite measurable systemic exposures to tenofovir. At necropsy, gross pathology provided no *in situ* evidence for tissue damage or target organ effects. Changes in several hematology, coagulation and clinical chemistry parameters that reached statistical significance were not considered test article related because they were typically sporadic, not dose-related, and were present in only one gender of rabbit on each occasion. Organ weight changes also reached statistical significance on occasion, but these were also considered not to be test article related for the same reasons cited above, i.e., sporadic and not dose-related. No tissues or organs other than the rectum and colon were examined for histopathological changes.

Rectal application of test articles resulted in measurable systemic concentrations of tenofovir at all dose levels, and after the first dose on Day 1 and the Day 14 dose. Tenofovir exposures were variable on Day 1; however, by Day 14 plasma concentrations were more consistent amongst individual animals and there was a clear dose-related increase in tenofovir exposures in both male and female rabbits. Systemic exposures to tenofovir were comparable in female and male rabbits. Absorption of tenofovir was relatively rapid, with the plasma T_{max} occurring at 1 hr on Day 1 (for most dose groups) and at 2 hr (female rabbits) and 4 hr (male rabbits) on Day 14. Mean C_{max} values on Day 1 ranged from 11.7 ng/mL (Group 4 females) to 59.0 ng/mL (Group 3 females), except Group 3 males where the C_{max} was 1182 ng/mL. Mean C_{max} values on Day 14 ranged from a low of 32.3 ng/mL (Group 3 males) to 265 ng/mL (Group 5 males). The mean T_{max} and C_{max} values for Group 3 males on Day 1 were skewed by one male rabbit with a very high tenofovir plasma

concentration at 24 hr post dose of 4210 ng/mL. The elimination half-life for tenofovir could not be determined with accuracy due to the variable exposures on Day 1, and a poorly defined terminal elimination phase on Day 14. For those groups where a half-life could be measured on Day 14, the $t_{1/2}$ for tenofovir ranged from 11.3 to 16.2 hours. It is possible that continued absorption of tenofovir from the rectal site of administration contributed to the inability to accurately measure half-life on Day 14. Tenofovir plasma concentrations increased in both female and male rabbits with increasing dose. However, the increase in exposure was somewhat less than dose proportional. On Day 14 when tenofovir plasma concentrations were most consistent across individual animals, the decrease in dose-proportional exposure for C_{max}/Dose between Group 3 (10 mg) and Group 5 (100 mg) was 66% and 18% for female and male rabbits, respectively. The decrease for AUC_{last}/Dose between Group 3 and Group 5 was 52% and 32% for female and male rabbits, respectively. There was a marked increase in tenofovir exposure over the 14 days of rectal administration. Accumulation ratios (AUC_{last} Day 14/AUC_{last} Day 1) varied from 7.2 to 23.7 across dose groups.

The No Observed Adverse Effect Level (NOAEL) for rectal administration of test article in this study was greater than the highest concentration tested, i.e., >10% tenofovir in vaginal gel (a 100 mg dose).

Pharmacokinetics

Pharmacokinetic studies have been performed for both vaginal and rectal administration of tenofovir gel.[73, 81-87]

Rat pK , Excretion, and Distribution Studies- Vaginal Administration

The pharmacokinetics, excretion and tissue distribution of ^{14}C-PMPA were evaluated in rats following intravaginal administration of an earlier formulation of tenofovir gel containing propylene glycol.[81] Four female rats received a single intravaginal dose administered as an aqueous gel containing 20 mg tenofovir/g. Plasma concentrations of total radioactivity were highly variable; this was attributed to inconsistent retention of the formulation within the vagina, or possibly oral absorption related to grooming. The apparent maximum plasma concentration (C_{max}) for tenofovir occurred at the earliest time point (15 minute), suggesting that absorption from the vagina was relatively rapid. Thereafter plasma concentrations declined with an approximate half-life of 1.6 hrs. The bioavailability of intravaginal tenofovir was estimated by comparison of the observed area under the curve $_{(0-24)}$ (AUC) with historical AUC data for an intravenous (IV) dose of 10 mg/kg tenofovir in rats (9.71 µg hr/mL). The observed systemic bioavailability of intravaginal tenofovir was 7.9%.

In the excretion and distribution study, two groups of four additional rats received a single intravaginal dose of ^{14}C-PMPA (10 mg/kg, 100 µCi/kg) administered as an aqueous gel containing 20 mg tenofovir/g. This study found that much of the dose was lost from the vaginal orifice by leakage. Vaginal tissue contained 0.1% of the dose and less than 0.01% of the dose was recovered in the ovaries and uterus.

Rabbit pK, Distribution, and Bioavailability Studies- Vaginal Administration

Single-dose pharmacokinetics of radio-labeled tenofovir gel in female rabbits has been previously examined (0.5 mL, 1% w/v tenofovir, 5 mg per animal, 50 µCi/kg).[82] Plasma concentrations of radioactivity were highest at the first sample time point (0.5 hour (hr)) and below the level of quantification at 24 hours. Pharmacokinetic parameters including the proportion of dose absorbed systemically could not be estimated, due to the very low plasma concentrations.

In a tissue distribution study using the same radio-labeled tenofovir 1% vaginal gel formulation, dose and strength as the above study, eighteen female rabbits were administered an intravaginal dose using a gavage needle.[73] An additional eighteen rabbits received an intravaginal dose of 3% w/v radio-labeled tenofovir (15 mg per animal). Analysis of vaginal tissue sections found no clear relationship between tissue concentration and dose, with no consistent pattern of distribution. Very little radioactivity was recovered in non-vaginal tissues. Concentrations in blood (0.002 to 0.047 µg-eq/g of tissue) exemplified the variability of distribution of the product although the effect of oral absorption due to grooming behaviors of the animals may have impacted these results.

Systemic and vaginal tissue bioavailability was assessed in female white New Zealand rabbits following single and multiple intravaginal doses (twice a day for 7 or 14 days) of 1 mL of tenofovir 1% gel or a single IV solution of 10 mg tenofovir.[73] Animals that were vaginally and intravenously dosed were sacrificed at the following timepoints: 1) 8 hours after single IV dose; 2) 4 hours after single vaginal dose; 3) 8 hours after single vaginal dose; 4) 4 hours after the thirteenth twice-daily vaginal dose; and, 5) 4 hours after the twenty-seventh twice-daily vaginal dose (see table below). After sacrifice, vaginal tissue was rinsed to remove topical tenofovir, and biopsy samples were taken. Both vaginal rinse and vaginal tissue were analyzed for tenofovir content. Systemic absorption following a single intravaginal dose was barely detectable, and only within the first 30 minutes. Multiple intravaginal administrations of tenofovir 1% gel and the single IV administration of 10 mg tenofovir resulted in systemic levels of tenofovir (see Table 2).

Table 2: Tenofovir Bioassay Data

	Mean 1st Rinse Vaginal Surface (nanogram(ng)/mL)	Mean Vaginal Tissue Concentration (ng/g)	C_{max} (ng/mL)	AUC (0-4 hr) (ng*hr/mL)
Single IV, 8 hr	**362** (19-990)	**950** (120-5,019)	**10,221**	**4,013** (3,192-4,503)
Single vaginal, 8 hr	**97** (7-415)	**940** (10-7,277)	**3**	--
Single vaginal, 4 hr	**1,441** (2-5,100)	**2,817** (35-11,780)	**5**	--
Twice daily x 7d vaginal, 4 hr	**1,086** (145-4,369)	**3,146** (448-14,429)	**239** (29-808)	**342** (54-1,037)
Twice daily x 14d vaginal, 4 hr	**3,361** (33-8,000)	**11,409** (245-50,102)	**71** (24-197)	**94** (12-229)

Macaque pK and Distribution Studies- Vaginal Administration

The pharmacokinetics (PK) of radio-labeled tenofovir gel was evaluated via plasma and vaginal biopsies collected from four rhesus macaques following single-dose intravaginal administration of tenofovir 1% vaginal gel.[73] Radioactivity was detected starting at 15 minutes post application, with peak concentration of tenofovir in vaginal tissue at 8 hrs and remaining high at 12 hours. No significant radioactivity was detected in whole blood or plasma.

Macaque pK Studies- Rectal Administration

Only preliminary assessments of single dose rectal administration of tenofovir 1% gel (PMPA) have been conducted in the setting of a pilot macaque efficacy trial.[83] Plasma samples were assayed for tenofovir concentration by the Clinical Pharmacology and Analytical Chemistry Core of the University of North Carolina Center for Acquired Immunodeficiency Syndrome (AIDS) Research. Drug concentrations in plasma were determined by a validated high pressure liquid chromatography (HPLC) method with ultraviolet detection[84]. This method utilized a dynamic range of 10 to 10,000 ng/mL, with intra- and inter-day variability of <10% across this range. Total tenofovir concentrations were assayed in tissues using a fully validated HPLC method with mass spectrometry detection.

Analysis of intestinal tissue samples collected at necropsy showed that all tenofovir-dosed animals had measurable concentrations of drug in lysates of colorectal tissue at concentrations between 20.8 and 54.2 µg/g protein but no drug was detected in lysates of homogenates from the small intestine. Tissues from untreated animals acted as negative controls. To indirectly estimate the amount of intracellular phosphorylated tenofovir in tissues, samples were analyzed with (to measure the combination of tenofovir + tenofovir monophosphate + tenofovir diphosphate) and without (to measure tenofovir only) phosphatase hydrolysis. Subtracting the concentration of tenofovir obtained from tissue samples without phosphatase, from the concentration of tenofovir obtained from tissue samples with phosphatase,

demonstrated that between 46-75% of total tenofovir in tissues was present as the intracellular monophosphate and diphosphate forms. Based on intracellular data describing tenofovir monophosphate: diphosphate ratios,[85-87] it was estimated that approximately 30-60% of total tenofovir in tissues was present as the intracellular diphosphate form. The relatively low rectal dose of tenofovir gel applied, an average of 10 µg/kg, resulted in a maximum plasma detection level of 0.19%, which was detected 15-minutes after rectal dosing.

Effectiveness

Efficacy studies have been performed for both vaginal and rectal administration of tenofovir gel.[73, 83, 88]

Macaque Efficacy Studies- Vaginal Administration

Six independent nonhuman primate studies provided some degree of evidence for efficacy using vaginally administered 1% or 10% gel (see Table 3).[73] Although these data are limited and a powered statistical determination as to the efficacy of tenofovir 1% gel versus 10% cannot be made, empirical examination of the efficacy data identifies tenofovir 1% gel as the lowest efficacious concentration tested when given within two hours of virus challenge. All studies used SIVmac251, a highly infectious SIV isolate, and Indian-origin rhesus macaques (with the exception of study 6). Study 1 demonstrated protection of all four macaques that received 10% tenofovir gel as compared to no protection in the 2 macaques that received placebo gel. Likewise in study 2, 11 of 15 macaques that received 1% or 10% tenofovir gel were protected as compared to no protection in the 5 untreated control macaques that received no gel product. In studies 3, 4, and 5, <100% of the untreated controls were infected making these data problematic to interpret.

Table 3: Use of Topical Tenofovir to Prevent Vaginal Transmission of SIV

Study*	Number of Exposures	Treatment	Time of Administration	Number Infected	Progesterone Pretreatment
1	2	1 mL vehicle	−24 h, 0 h, 24 h, 48 h	2 of 2	No
		10% tenofovir	−24 h, 0 h, 24 h, 48 h	0 of 4	
2	1	untreated control	N/A	5 of 5	No
		10% tenofovir	−24 h , −15 m, +24 h	1 of 5	
		1% tenofovir	−24 h , −15 m, +24 h	1 of 5	
		1% tenofovir	−15 m	2 of 5	
3	1	untreated control	N/A	2 of 5	No
		vehicle	−15 m	1 of 5	
		1% tenofovir	−15 m	1 of 5	
		1% tenofovir	−2 h	3 of 5	
		1% tenofovir	−8 h	1 of 5	
4	1	untreated control	N/A	4 of 5	No
		vehicle	−15 m	2 of 5	
		1 % tenofovir	−15 m	1 of 5	
		1 % tenofovir	−2 h	1 of 5	
		1 % tenofovir	−8 h	2 of 5	
5	1	untreated control	N/A	2 of 5	No
		vehicle	−2 h	2 of 5	
		1 % tenofovir	−2 h	0 of 5	
6	1	1 % tenofovir	−12 h	5 of 8	Yes
		vehicle	−12 h	8 of 8	
		1 % tenofovir	−24 h	8 of 8	
		vehicle	−24 h	8 of 8	
		untreated control	N/A	8 of 8	
		1 % tenofovir	−72 h, −48 h, −24 h	6 of 8	

* All studies were performed with the SIVmac251 isolate of SIV, and female rhesus macaques were inoculated intravaginally. Virus challenges were performed without progesterone pretreatment in Studies 1–5; macaques in Study 6 were pretreated with 30 mg Dep-Provera 30 days prior to viral challenge. The indicated studies were performed by 3 independent investigators with Studies 2, 3, 4, and 5 being performed by the same laboratory.

Study 6 was different from the first five studies in that Chinese-origin rhesus macaques were used and they were pretreated with progesterone before virus challenge to enhance susceptibility to infection and synchronize reproductive cycles. This study was designed to determine whether topical dosing of tenofovir gel could be disassociated from the coital act while remaining an effective microbicide, in a regimen consistent with the long intracellular half-life of the active metabolite, tenofovir diphosphate. A total of 48 macaques, pretreated with a 30 mg dose of depot medroxyprogesterone acetate (DMPA) 30 days prior to viral challenge, were divided into 6 groups of 8 animals each. Group 1 received one topical vaginal dose of tenofovir 1% gel 12 hours prior to one intravaginal viral challenge with a dilution of SIVmac251 stock representing approximately 50 $TCID_{50}$ (50% tissue culture infective dose). In parallel, Group 2 received matched placebo gel. Group 3 received a single dose of tenofovir 1% gel twenty-four hours prior to viral challenge. The matched placebo gel was administered to Group 4 twenty-four hours prior to viral challenge. Group 5 was an untreated control group receiving only the viral challenge. A single dose of tenofovir 1% gel was administered topically to Group 6

animals at 72, 48, and 24 hours prior to viral challenge. Thus, Group 6 animals received 3 consecutive days of gel; Group 4 served as the placebo control for Group 6. Based on plasma viral load, all untreated control animals became infected as did all placebo gel-treated macaques. Three animals were protected from infection in Group 1 receiving a single dose of tenofovir 1% gel 12 hrs prior to virus exposure. Although no macaques receiving a single dose of tenofovir 1% gel 24 hours prior to virus exposure were protected, two of eight animals in Group 6 receiving multiple doses of tenofovir 1% gel remained uninfected. Infection status was confirmed using virus co-culture, seroconversion and lymph node deoxyribonucleic acid polymerase chain reaction (DNA PCR). These data show 24 of 24 placebo gel-treated or untreated macaques became infected with SIVmac251 while 5 of 24 macaques were protected from SIV infection by vaginally administered tenofovir 1% gel.

Progesterone pretreatment (30 mg DMPA) is used in macaque studies to increase susceptibility to infection by a mechanism thought to involve thinning of the vaginal epithelium. It is generally required to achieve 100% infection in untreated control animals challenged with less infectious Simian/Human Immunodeficiency Virus (SHIV) chimeric viruses. Although animals were pretreated with DMPA in this study but not the previous studies as shown in Table 3 (studies 1−5), this pretreatment may not be required for such a highly infectious virus as SIVmac251. In view of the potent infectivity of this virus, the lack of an endpoint in the animal titration of this stock[88], and increased susceptibility resulting from progesterone pretreatment, it is possible that the amount of virus used was too high, thereby masking any protective effect. Further studies are required to understand the factors that impact protection by intravaginal tenofovir gel in the macaque model.

Macaque Efficacy Studies- Rectal Administration

The rectal application of tenofovir was evaluated for protective efficacy against rectal challenge with simian immunodeficiency virus (SIV) in a well established and standardized pre-clinical macaque model[83]. A total of 20 purpose-bred Indian rhesus macaques were used to evaluate the protective efficacy of topical tenofovir. Six animals received tenofovir 1% gel *per rectum* 15 minutes prior to virus challenge and 3 macaques received tenofovir 1% gel per rectum 2 hours prior to virus challenge, whereas 4 macaques received placebo gel and 4 macaques remained untreated. In addition, 3 macaques were given tenofovir gel 2 hours after virus challenge. Following intrarectal instillation of 20 median rectal infectious doses (MID_{50}) of a non-cloned, virulent stock of $SIV_{mac251/32H}$ all animals were analyzed for virus infection, by virus isolation (VI) from peripheral blood mononuclear cells (PBMC), quantitative proviral DNA load in PBMC, plasma viral ribonucleic acid (vRNA) load by sensitive quantitative competitive (qc)-RT PCR and presence of SIV-specific serum antibodies by ELISA. A significant protective effect was seen (p=0.003; Fisher's Exact Probability test) wherein 8 of 9 macaques given tenofovir *per rectum* either 15 minutes or 2 hours prior to virus challenge were protected from infection (n=6) or had modified virus outcomes (n=2) while 4 of 4 untreated macaques and 3 of 4 macaques given placebo gel were infected, as were 2 of 3 animals receiving

tenofovir gel after challenge. Moreover, analysis of lymphoid tissues *post mortem* failed to reveal sequestration of SIV in the protected animals.

Colorectal explants from non-SIV challenged tenofovir treated macaques were resistant to infection *ex vivo*, whereas no inhibition was seen in explants from the small intestine. Tissue-specific inhibition of infection was associated with the intracellular detection of tenofovir. In colorectal explants from 3 of 4 animals, complete or nearly complete inhibition of virus replication was seen and in the other animals, a high level of variability between replicate samples resulted in lower mean inhibition. In contrast, inhibition of virus replication was not seen in explants from the small intestine suggesting that tenofovir was, at least in part, acting on cells at the virus portal of entry.

Analysis of plasma tenofovir concentration at the time of virus challenge, 15 minutes after gel administration, revealed a strong positive association with protective efficacy. The lowest concentration of plasma tenofovir associated with protection was 119.9 ng/mL. Taking into account estimated plasma volume, protection was associated with as little as 0.11% of the total tenofovir applied; however, this is systemic exposure, rather than local exposure. Moreover, an effect upon plasma viremia was observed with as little as 0.06% of applied tenofovir detected in plasma at 15 minutes. In animals given tenofovir 2 hours prior to virus challenge, plasma tenofovir concentrations at the time of challenge ranged between below the 10 ng/mL limit of detection to 23.3 ng/mL. These results therefore suggest that drug concentration peaks rapidly after rectal dosing. Interestingly ileum/jejunum tissue taken from dosed macaques remained susceptible to infection, and was confirmed by the lack of detectable drug in these tissues. This suggests that secondary distribution to this site is insignificant and supports the importance of comparing an oral, systemically-delivered dose to a topical, locally-delivered dose.

2.4.4.3 Human Studies

MTN-007 and this study will be the first rectal safety studies of the newly reformulated tenofovir 1% gel. Vaginal studies of the new reformulation are also in the planning stages. However, a broad range of studies have been conducted with the old formulation; data are summarized below.

<u>Pharmacokinetics</u>
Data from “Phase 1 Safety and Acceptability Study of the Vaginal Microbicide Agent PMPA Gel”, also known as HPTN 050 has been published.[89] Eighty-four (60 HIV negative and 24 HIV positive) women applied either 0.3% or tenofovir 1% gel once or twice daily for 14 days. Systemic absorption was limited (maximum serum levels 3.1-25.8 ng/mL).

In MTN-002, the first microbicide trial to be conducted during pregnancy, 16 women received a single vaginal dose of tenofovir 1% gel prior to elective cesarean section.

Tenofovir levels were measured in blood, amniotic fluid, cord blood, endometrial tissue, and placental tissue. Plasma tenofovir levels were compared to historical controls. Study results demonstrated that the PK levels of a single vaginal dose of tenofovir 1% gel in pregnant women was similar to those found in non-pregnant women and that serum tenofovir levels were up to 50 – 100 times less as compared to standard oral dosing[90]. Additionally, tenofovir was shown to get to the fetal compartment with low overall cord levels (~40 times less than oral dosing), but with a similar cord blood: maternal ratio. Overall findings suggest that tenofovir is safe for use in term pregnancy and warrants additional investigation during pregnancy.

Safety

In HPTN 050, the tenofovir 1% gel formulation was well tolerated in both HIV-uninfected and -infected women.[89] Further, 94% of female participants and 81% of male participants indicated they would definitely or probably use tenofovir gel in the future. While a number of participants (92%) reported some type of adverse event (AE), the majority of them were mild (87%) and limited to pruritus (n = 18), erythema (n=14), petechiae/ecchymosis (n = 14), vaginal discharge (n = 13), and burning (n = 10). Only four severe AEs were reported, but of these, only one (lower abdominal pain) was thought to be product-related. Product concentration, sexual activity and HIV status were not associated with a specific AE pattern. No clinically significant systemic toxicity was observed. No serious adverse events (SAEs) were reported.

In a male tolerance study (CONRAD A04-099/IND 73,382), tenofovir 1% gel was well tolerated in men following seven days of once daily exposure, for 6 to 10 hours, to the penis. There were few reported and observed genital findings after product use including mild pain (burning, irritation, discomfort) and pruritis[73]. All observed findings were classified as mild, small in size and requiring no treatment. Reported symptoms were mild, of short duration and resolved by the final visit. There were no noticeable differences between signs and symptoms of genital irritation in the circumcised compared to uncircumcised group.[73, 91]

A Phase 2 study of tenofovir 1% gel (HPTN 059) has completed follow up. This study assessed safety and acceptability of, and adherence to a regimen of tenofovir gel for vaginal use in HIV-uninfected women versus a placebo gel.[92] Exploratory objectives included measurement of vaginal flora characteristics, assessment of the effects of gel on genital cytokine and chemokine expression, and the evaluation of cytokine and chemokine expression to correlate expression with evidence of inflammation, epithelial disruption and genital symptoms. The study was a four-arm, three-site, randomized, controlled trial comparing gel used once daily and gel used prior to intercourse, to placebo gel, with 6 months gel exposure and follow-up. The study was conducted among 200 women in Pune, India; Birmingham, Alabama, USA; and New York, New York, USA. Participants were sexually active, HIV-uninfected women between ages 18 and 50, but not menopausal or post menopausal. Participants had six months of study gel exposure and follow-up. They were randomized to either once daily or coitally dependent group, and received

either tenofovir or placebo gel. Participants received single use unit dose tubes and single-use applicators.

No statistically significant differences were seen between those receiving active and placebo gels in complete blood count (CBC), liver function tests, or renal function tests. Among those using a study gel daily, no participants had pelvic exam findings involving generalized erythema or severe edema or deep epithelial disruption at any follow-up visit during the study. At the Week-24 Visit, no participants had exam findings suggestive of vaginitis, cervicitis, superficial disruption, disrupted blood vessels, or intermenstrual bleeding. Adherence to study gel was high, and was supported by PK data. 79% of women reporting gel use in past 12 hrs had low but detectable plasma tenofovir supporting self-reported adherence data. Daily and coital use was highly acceptable to women. These data suggest a favorable safety and acceptability profile of tenofovir gel, and support routine monitoring for genital findings among women without genital symptoms at six month intervals.[92]

A Phase 2b study of vaginally-administered tenofovir 1% gel use (CAPRISA 004) has recently completed follow-up and data analysis.[6] This study, conducted among sexually active, HIV-uninfected women at an urban and rural site in South Africa, compared the safety and effectiveness of tenofovir 1% gel when use within 12-hours before and after intercourse, versus placebo gel (HEC). Safety assessments as well as HIV and urine pregnancy tests were performed at monthly follow-up visits. Pelvic exams were also performed at quarterly visits.

Study results suggest that vaginally-administered, coitally-dependent use of tenofovir 1% gel is safe. No increases in renal, hepatic, pregnancy-related, or genital AEs were observed. Additionally, tenofovir 1% gel was shown to reduce HIV infection by approximately 39% regardless of sexual behavior, condom use, HSV-2 infection, or urban/rural location. It is important to note, however, that the high acceptability rate (~97%) did not correspond to the average adherence rate (~61%). While these data suggest a favorable safety and effectiveness profile for tenofovir 1% gel, further studies must be done to assess whether more frequent (e.g. daily) dosing will enhance adherence and as a result, effectiveness, without compromising participant safety, and whether tenofovir 1% gel is safe, well-tolerated, and efficacious when administered rectally.

Drug Resistance

In HPTN 050, no new resistance mutations evolved in plasma or cervicovaginal lavage after 14 days of tenofovir gel use, but 3 women had plasma mutations associated with low level tenofovir resistance identified at both Day 0 and Day 14 (M41L, L210M, ±T215I/Y).[89]

Other Studies of Tenofovir for HIV Prevention

Several other studies of the safety and/or effectiveness of topical tenofovir 1% gel as an HIV prevention strategy are summarized below in Table 4.

Table 4: Other Studies of Tenofovir 1% Gel

Location	Sponsor	Population	Design
USA, Dominican Republic	CONRAD A04-095/IND 73,382	Sexually abstinent women	PK study; single dose and 14-day once or twice-daily.
South Africa, Uganda, USA	DAIDS/MTN-001/IND 55,690	Sexually active women	Phase 2 Adherence and Pharmacokinetics Study of Oral and Vaginal Preparations of Tenofovir
USA	DAIDS IPCP/RMP-02/MTN-006/CONRAD IND 73,382	Sexually abstinent (for active phases of study and for 5 days following biopsy collection) women and men	Phase 1 Rectal PK and Acceptability

Studies examining the safety and/or effectiveness of oral formulations of tenofovir as a prevention strategy are summarized in Table 5 below.

Table 5: PrEP Studies

Location	Sponsor	Population	PrEP Strategy
**Phase 2**			
West Africa (Ghana, Nigeria, Cameroon)	Family Health International	936 high-risk women	TDF
United States	Centers for Disease Control and Prevention (CDC)	400 men who have sex with men	TDF
**Phase 3**			
Thailand	CDC	2,000 injection drug users (~20% women)	TDF
Botswana	CDC	1,200 men and women	FTC/TDF
Peru, Ecuador, Brazil, Thailand, South Africa, United States	NIH<br><br>(iPrEx Study, IND 71,859)	1,400 men who have sex with men (potential expanded sample size of 3,500)	FTC/TDF
Africa	Family Health International	3,800 high-risk women	FTC/TDF
Africa	University of Washington, Gates Foundation	3,900 HIV-1 seronegative partners within HIV-1 discordant couples	FTC/TDF<br>TDF

On July 16, 2012, the US FDA approved the use of Truvada (TDF and emtricitabine), to be taken once daily in combination with safer sex practices, to reduce the risk of sexually acquired HIV-1 infections in adults who are at high risk of becoming infected with HIV-1.

2.4.5 Justification of Dosing

The primary rationale for selecting the newly reformulated tenofovir 1% vaginal gel is that until a rectal specific formulation of tenofovir is developed, the only product (and volume of tenofovir gel) available will be the vaginal product.

The choice of the tenofovir 1% vaginal gel concentration is based on both the animal and clinical evidence described above suggesting an appropriate safety profile and potency. Animal and human studies have demonstrated minimal vaginal irritation at this concentration. Rectal administration has demonstrated efficacy in a macaque model[83]. Finally, limited vaginal PK tenofovir data in nonhuman primates demonstrate that tenofovir gel is broadly distributed in vaginal tissues following vaginal application and can penetrate to epithelial tissues.[73] The amount of tenofovir administered by intravaginal application of 4 grams (g) of a 1% dose (40 mg) is highly active against HIV and results in a reduction of plasma HIV ribonucleic acid (RNA) of 1.5 log10 copies/mL after daily administration for 21 days. There are no published studies of drug penetration into human colonic tissue after either rectal or oral administration of tenofovir, though these studies are ongoing. However, colon tissue penetration of tenofovir should exceed tenofovir vaginal tissue penetration given the single columnar colon epithelial layer in contrast to 40 cell layers in the stratified squamous epithelium of the vaginal mucosa.

3 STUDY HYPOTHESES

3.1. Stage 1A

This study hypothesizes that STIs and anorectal pathologies will be present in the study population and that standard anoscopy with examination by the naked eye will be as effective in identifying anorectal pathologies as high-resolution anoscopy.

3.2. Stage 1B

This study hypothesizes that characteristics of the gel and applicator as well as pre-coital behaviors will impact the acceptability of and use-adherence to a microbicide gel.

3.3. Stage 2

The study hypothesizes that eight rectal applications of the new tenofovir 1% gel formulation will be safe, well-tolerated, and acceptable among healthy, ethnically diverse, young MSM.

4 OBJECTIVES

4.1. Clinical Objectives:

Stage 1AB

- Primary objective: To determine the prevalence of STIs and anal and rectal pathologies that may facilitate HIV infection

- Secondary objective: To determine whether standard anoscopy with examination by the naked eye is as effective in identifying anorectal pathologies as high-resolution anosocopy (HRA)

Stage 2

- Primary objective: To evaluate the safety of tenofovir 1% gel when applied rectally
- Exploratory objective: To determine whether use of tenofovir 1% gel is associated with rectal mucosal damage

4.2. Behavioral Objectives:

Stage 1AB

- Primary objective: To determine the acceptability of and use-adherence to a placebo gel delivered rectally with a rectal delivery device
- Secondary objective: To determine the prevalence of behavioral practices associated with anal intercourse that may detract from microbicide effectiveness
- Tertiary objective: To determine the feasibility of recruitment and retention of MSM with high risk sexual behavior, such as sex workers, for microbicide studies, and their likelihood to use non-condom based HIV prevention strategies

Stage 2

- Primary objective: To determine the acceptability of and use-adherence to a placebo gel or tenofovir 1% gel delivered rectally with a vaginal delivery device
- Secondary objective: To determine the prevalence of behavioral practices associated with anal intercourse that may detract from microbicide effectiveness

5 STUDY DESIGN

5.1. Identification of Study Design

A two-stage longitudinal study: a clinical and behavioral evaluation (Stage 1A) with an acceptability and adherence trial (Stage 1B), followed by a Phase 1 randomized, double-blind, multi-site, placebo-controlled trial (Stage 2). Initially 280 sexually active young MSM, including 40 sex workers, will be enrolled in Stage 1A. The first 140 eligible participants, including 20 sex workers, who complete the clinical and behavioral evaluation (Stage 1A) will continue on to the acceptability and adherence trial (Stage 1B) during which they will receive HEC placebo gel. The first 24 eligible participants to complete Stage 1B will continue onto Stage 2, the Phase 1 randomized (1:1 ratio), double-blinded, multi-site, placebo-controlled trial of the tenofovir 1% gel.

Figure 1. Study Schema

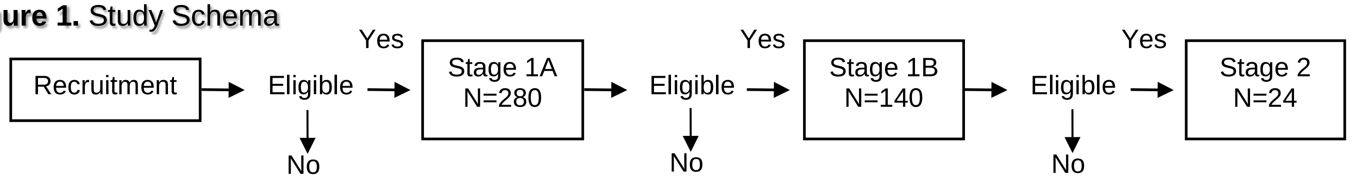

5.2. Summary of Major Endpoints

5.2.1 Clinical Endpoints:

Stage 1AB

- <u>Primary endpoint</u>: Presence of STIs and anal and rectal pathologies as detected by standard anoscopy
- <u>Secondary endpoint</u>: Percent of agreement between reports of anal and rectal pathologies by 2 assessment methods (standard anoscopy versus high-resolution anoscopy)

Stage 2

- <u>Primary endpoint</u>: Grade 2 or higher AEs, as defined by the Division of AIDS Table for Grading the Severity of Adult and Pediatric Adverse Events, Version 1.0, Dec 2004 and/or Addenda 3 (Rectal Grading Tables for Use in Microbicide Studies)
- <u>Exploratory endpoints</u>: Changes in the following parameters:
  - Intestinal histopathology
  - Intestinal mucosal mononuclear cell phenotype
  - Intestinal mucosal cytokine messenger RNA (mRNA)
  - Cytokine profile in rectal secretions

5.2.2 Behavioral Outcomes:

Stage 1AB

- <u>Primary outcomes</u>:
  - Identification of factors related to acceptability and adherence
  - Proportion of participants who report via the acceptability questionnaire that they would be very likely to use a similar candidate microbicide gel during receptive anal intercourse (RAI)
  - Proportion of RAI episodes in which the gel was used by participants
  - Comparison between self-reports of gel use and applicator counts
- <u>Secondary outcomes</u>:
  - Identification of factors related to sexual behaviors (e.g., douching, lubricant use, recreational drug use, condom use, partner selection)
  - Prevalence of risky sexual practices, douching, lubricant use, recreational drug use, and condom use

- Tertiary outcomes:
  - Proportion of individuals identifying as sex workers screened to individuals enrolled in the sex worker cohort
  - Proportion of visits completed by participants enrolled in the sex worker cohort
  - Likelihood of using microbicide gels, PrEP, or a rapid HIV Home Test with clients and non-commercial partners for individuals identifying as sex workers

Stage 2

- Primary outcomes:
  - Identification of factors related to acceptability and adherence
  - Proportion of participants who report via the acceptability questionnaire that they would be very likely to use a similar candidate microbicide gel during receptive anal intercourse (RAI)
  - Proportion of RAI episodes in which the gel was used by participants
  - Comparison between self-reports of gel use and applicator counts
- Secondary outcomes:
  - Identification of factors related to sexual behaviors (e.g., douching, lubricant use, recreational drug use, condom use, partner selection)
  - Prevalence of risky sexual practices, douching, lubricant use, recreational drug use, and condom use

5.3. Description of Study Population

The study population will be ethnically diverse, HIV-uninfected MSM, age 18-30 who meet the criteria outlined in Section 6.2 and 6.3.

5.4. Time to Complete Accrual

Accrual is expected to be complete within approximately 1 year.

5.5. Study Groups

Stage 1A- Approximately 280 MSM between the ages of 18-30 will be enrolled, including a subset of 40 sex workers

Stage 1B- Approximately 140 participants from Stage 1A will progress to Stage 1B; including a subset of 20 sex workers

Stage 2- Approximately 24 eligible participants from Stage 1B will progress to Stage 2 and be randomized at a 1:1 ratio to tenofovir 1% gel or HEC placebo gel.

5.6. Expected Duration of Participation

Each participant completing Stages 1A, 1B, and 2 will be on study for approximately 6 months. The total duration of the study will be approximately 1.5 years.

6 STUDY POPULATION

6.1. Selection of Study Population

The inclusion and exclusion criteria in Sections 6.2 and 6.3 will be utilized to ensure the appropriate selection of study participants for this study.

6.1.1 Recruitment

Members of the research teams at the study sites will recruit from a diversity of sources such as various clinical sites at which they are providing direct patient care to potential study participants. Some sites will also recruit from the greater academic community, related catchment areas, and volunteers from previous research studies if those participants have previously signed an authorization permitting this type of contact, and postings in newspapers, on the Internet, and at community-based organizations. The study sites have experience in identifying and recruiting men who have sex with men (MSM) with a history of RAI into behavioral and/or clinical studies. Site Institutional Review Board (IRB)-approved recruitment materials will be used. Written informed consent will be obtained prior to the initiation of any study-related procedures.

6.1.2 Retention

Each site will establish participant retention procedures. Study site staff members at each site are responsible for developing and implementing retention procedures and efforts.

6.2. Inclusion Criteria

Inclusion Criteria for Participants in Stage 1A (non sex-workers):

1. Willing and able to provide written informed consent to take part in the study
2. Willing and able to communicate in English or Spanish
3. Must agree not to participate in other drug trials
4. Biologically male and identifies as male
5. Age 18-30 years at screening
6. HIV-1 status antibody negative or unknown per patient report
7. Understands and agrees to local STI reporting requirements
8. Able and willing to provide adequate information for locator purposes
9. Availability to return for all study visits, barring unforeseen circumstances
10. A history of consensual RAI at least once in past month
11. Reporting at least one occasion of unprotected RAI in the prior year*
    In order to identify participants at increased risk for acquiring HIV

Inclusion Criteria for Participants in Stage 1A (sex-workers):

1. Willing and able to provide written informed consent to take part in the study
2. Willing and able to communicate in English or Spanish
3. Must agree not to participate in other drug trials
4. Biologically male, including male-to-female transgender women
5. Age 18-30 years at screening
6. HIV-1 status antibody negative or unknown per patient report
7. Understands and agrees to local STI reporting requirements
8. Able and willing to provide adequate information for locator purposes
9. Availability to return for all study visits, barring unforeseen circumstances
10. A history of consensual RAI at least once in past month
11. Reporting at least one occasion of unprotected RAI in the prior year*
12. Reporting at least two occasions of RAI as part of transactional sex (i.e., having received money or other goods/services in exchange for sex) in the prior 2 months*

    **In order to identify participants at increased risk for acquiring HIV*

Inclusion Criteria for Participants in Stage 1B (both sex workers and non sex-workers):

1. Completed Stage 1A
2. HIV-1 uninfected at screening based on HIV rapid test results
3. Reports unprotected RAI in the prior three months on at least one occasion*
   In order to obtain acceptability data from individuals most likely to benefit from microbicide availability
4. Availability to return for all Stage 1B visits, barring unforeseen circumstances

Inclusion Criteria for Participants in Stage 2:

1. Completed study stage 1AB
2. HIV-1 uninfected at screening based on HIV rapid test results
3. Availability to return for all Stage 2 visits, barring unforeseen circumstances

6.3. Exclusion Criteria

Exclusion Criteria for Participants in Stage 1A (both sex workers and non sex-workers):

1. Any condition or prior therapy that, in the opinion of the investigator, would make study participation unsafe, make the individual unsuitable for the study or unable to comply with the study requirements. Such conditions may include, but are not limited to, current or recent history of severe, progressive, or uncontrolled substance abuse, or renal, hepatic, hematological, gastrointestinal, endocrine, pulmonary, neurological, or cerebral disease

Exclusion Criteria for Participants in Stage 1B (both sex workers and non sex-workers):

1. Clinical or laboratory diagnosis of active rectal infection requiring treatment per current CDC guidelines. Infections requiring treatment include Chlamydia (CT), gonorrhea (GC), syphilis, active HSV lesions, chancroid, genital sores or ulcers, and

if clinically indicated, genital warts. Note that an HSV-2 seropositive diagnosis with no active lesions is allowed, since treatment is not required

2. Positive Hepatitis B surface antigen test indicating hepatitis B infection
3. Allergy to methylparaben, propylparaben, or latex
4. History of significant drug allergy or recurrent urticaria
5. History of inflammatory bowel disease
6. Currently engage or plan to engage in unprotected RAI with HIV-infected partners
7. By participant report planning to receive another investigational drug while participating in this study
8. Any other condition or prior therapy that, in the opinion of the investigator, would make study participation unsafe, make the individual unsuitable for the study or unable to comply with the study requirements. Such conditions may include, but are not limited to, current or recent history of severe, progressive, or <u>uncontrolled substance abuse</u>, or renal, hepatic, hematological, gastrointestinal, endocrine, pulmonary, neurological, or cerebral disease

Exclusion Criteria for Participants in Stage 2:

1. Meet any of the exclusion criteria for Stage 1B
2. Reporting a history of transactional sex (i.e., having received money or other goods/services in exchange for sex) in the prior 6 months
3. Undergoing or completed gender reassignment
4. Grade 2 or higher liver function, creatinine, coagulation, electrolyte, or hematology abnormality in accordance with DAIDS toxicity table values (normal values based on site specific laboratory criteria) at screening (or Visit 2 PT/INR for coagulation), and confirmed by retest/and or redraw
5. History of significant gastrointestinal bleeding
6. History of inflammatory bowel disease
7. Abnormalities of the rectal mucosa, or significant rectal symptom(s), which in the opinion of the clinician represents a contraindication to biopsy (including but not limited to presence of any unresolved injury, infectious or inflammatory condition of the local mucosa, and presence of symptomatic external hemorrhoids
8. Per participant report, anticipated use and/or unwillingness to abstain from the following medications during the period of Stage 2 study participation:
    a. Heparin, including Lovenox®
    b. Warfarin
    c. Plavix® (clopidogrel bisulfate)
    d. Aspirin >81 mg per day
    e. Non-steroidal anti-inflammatory drugs (NSAIDS)
    f. Any other drugs that are associated with increased likelihood of bleeding following rectal biopsy
9. By participant report, use of systemic immunomodulatory medications within the 4 weeks prior to the Stage 2 Enrollment Visit and throughout study participation
10. By participant report, use of rectally administered medications, rectally administered products (including condoms) containing N-9, or any investigational products within the 2 weeks or 10 half-lives of the drug, whichever is longer, prior to the Stage 2

Enrollment Visit, or is planning to receive another investigational drug while participating in this study

11. Any other condition or prior therapy that, in the opinion of the investigator, would make study participation unsafe, make the individual unsuitable for the study or unable to comply with the study requirements. Such conditions may include, but are not limited to, current or recent history of severe, progressive, or uncontrolled substance abuse, or renal, hepatic, hematological, gastrointestinal, endocrine, pulmonary, neurological, or cerebral disease

7 STUDY PRODUCT

7.1. Regimen

Stage 1B

Each participant will receive a package of 20 doses of HEC placebo gel at Visit 2 and up to 20 additional applicators approximately 6 weeks later at Visit 3.

All participants will be instructed to insert the entire contents of one applicator rectally within 90 minutes prior to each episode of RAI. Participants will also be instructed to place the used applicators without washing them into individual plastic bags with a zip enclosure labeled “Used Applicators” and to return the used and unused applicators at the next study visit.

Stage 2

Each participant will be randomized to one of two blinded study regimens. Participants will receive either tenofovir 1% gel or HEC placebo gel.

Study participants will receive one dose of study product, at the Treatment 1 Visit (Visit 7), under observation.

At the Treatment 2 Safety Clearance Visit (Visit 9), the participant will receive his assigned package with the remaining 8 applicators. This will provide the participant an extra dose should one of the applicators become unusable for any reason. All participants will be instructed to insert the entire contents of one applicator rectally daily at night before bed, or before longest period of rest. Participants will be encouraged to have their partners use the condoms provided by the study staff.

In Stage 2, there will be at least a 7-day washout period between the Treatment 1 visit (Visit 7) and Treatment 2 Safety Clearance visit (Visit 9). The participant will have a maximum of 7 days to initiate a consecutive 7-day regimen.

7.2. Administration

Stage 1B

Study staff will instruct participants in proper methods of administering and storing their study product (HEC placebo gel). Participants will be instructed to insert one dose (the entire contents of one applicator) of gel into the rectum within 90 minutes of anticipated RAI. Participants will also be encouraged to have their partners use the condoms provided by the study staff.

Stage 2

Study staff will instruct participants in proper methods of administering and storing their study product tenofovir 1% gel or HEC placebo gel.

Based on randomization number, each participant will be assigned a package of 9 applicators. From this assigned package the participant will receive one applicator for administration at the Treatment 1 Visit (Visit 7). At this visit, the participant's first dose (the entire contents of one applicator) of study product will be administered or supervised by the IoR or designee.

At the Treatment 2 Safety Clearance Visit (Visit 9), the participant will receive their assigned package with the remaining 8 applicators. During the period of daily administration study participants will be instructed to insert one dose (the entire contents of one applicator) of gel into the rectum once daily throughout the 7-day period. Rectal administration of study product should occur before bedtime, usually in the evening, or the longest period of rest. If the product is used in the context of RAI, participants will be encouraged to have their partners use the condoms provided by the study staff.

If a participant misses a dose, he must insert rectally the missed dose as soon as possible, unless the next dose is estimated to be due within 6 hours. If the next dose is estimated to be due within 6 hours, the missed dose must be skipped. The next dose will be inserted rectally as originally scheduled.

7.3. Study Product Formulation

7.3.1 HEC Universal Placebo Gel for Stage 1B and Stage 2

The placebo gel, sometimes called the "Universal Placebo Gel" contains hydroxyethyl cellulose as the gel thickener, purified water, sodium chloride, sorbic acid and sodium hydroxide.[63] The gel is isotonic, formulated at a pH of 4.4, and has minimal buffering capacity to avoid the inactivation of sexually transmitted pathogens. Hydroxyethyl cellulose, the gelling agent, is used to approximate the viscosity of other microbicide gel candidates.

7.3.2 Tenofovir 1% Gel for Stage 2

Tenofovir 1% gel (weight/weight) is a gel formulation of tenofovir (PMPA, 9-[(R)-2-(phosphonomethoxy)propyl]adenine monohydrate), formulated in purified water with

edetate disodium, citric acid, glycerin, methylparaben, propylparaben, and hydroxyethyl cellulose, with a pH adjusted to 4-5. Tenofovir 1% gel is a transparent, viscous gel.

7.3.3 Comparison of HEC Placebo Gel and Tenofovir 1% Gel

Chemical Name	HEC Placebo Gel % w/w	New RGVF of tenofovir gel % w/w
Glycerin	0	5.00
Hydroxyethyl cellulose	2.70	3.0
Carbopol 974	0	0
Sodium carboxymethylcellulose	0	0
Sodium chloride	0.85	0
Methylparaben	0	0.22
Propylparaben	0	0.05
Purified water	96.3	89.66
Disodium edetate	0	0.05
Citric acid	0	1.00
Sorbic acid	0.10	0
PMPA	0	1.00
Sodium hydroxide	As needed	As needed
Diluted hydrochloric Acid	0	As needed
pH	4.4	4.5
Osmolality (mmol/kg)	isotonic	846

7.4. Study Product Supply and Accountability

7.4.1 Study Product Supply

7.4.1.1 HEC placebo gel for Stage 1B

The HEC placebo gel for Stage 1B will be manufactured under direction from the Principal Investigators, by DPT Laboratories, Ltd., (San Antonio, TX USA), which is a contract manufacturing facility. DPT Laboratories will manufacture the HEC gel and analyze/release the gels under cGMP. DPT will fill the applicators with HEC gel to create and package pre-filled applicators. Each identical, pre-filled, opaque white, single-use plastic applicator will contain a dose of approximately 6.65 mL of the HEC gel to deliver a dose of approximately 4 mL. The applicator measures approximately 14.5 cm long and 2.5 cm wide, and has a barrel-and-plunger design with extended wings for placement of fingers and a Fleet® comfort tip. Each filled applicator will be wrapped individually. A mechanism on the applicator prevents refilling and reuse.

7.4.1.2 HEC placebo gel for Stage 2

The HEC universal placebo gel for Stage 2 will be supplied by CONRAD (Arlington, VA, USA). Under direction from CONRAD, DPT Laboratories will manufacture the HEC placebo gel and analyze/release the gels under cGMP. DPT Laboratories will fill the applicators with HEC placebo gel to create pre-filled applicators and package each applicator and plunger in a wrapper. Each pre-filled applicator will contain and deliver a dose of approximately 4 mL of HEC gel.

7.4.1.3 Tenofovir 1% Gel for Stage 2

Tenofovir 1% gel will be supplied by CONRAD (Arlington, VA, USA). Under direction from CONRAD, DPT Laboratories will manufacture the tenofovir 1% gel and analyze/release the gels under cGMP. DPT Laboratories will fill the applicators designed for vaginal use with tenofovir 1% gel to create pre-filled applicators and package each applicator and plunger in a wrapper. Each pre-filled applicator will contain and deliver a dose of approximately 4 mL of tenofovir 1% gel (equal to 4.4 g).

7.4.2 Study Product Receipt

Study staff will be required to maintain complete study records of all study product supplies received. These records will contain documentation of receipt as well as dispensing of all study supplies.

7.4.3 Storage

Study participants will be instructed to store their applicators at the recommended storage conditions, and away from direct sources of light and heat, in an area out of reach of children. Study products will be stored in accordance with the protocol.

HEC gel should be stored at 25°C (77°F). Excursions are permitted between 15°C and 30°C (59°F and 86°F).

Tenofovir 1% gel must be stored at controlled room temperature, 25°C (77°F), at all times. Excursions are permitted between 15°C and 30°C (59°F and 86°F).

Storage conditions for protocol-provided study products will include segregation, security, and temperature monitoring, as well as appropriate conditions of light, moisture, ventilation and sanitation. Study products should be stored in a limited access area that is locked when not in use. The study products should be accessible only to authorized personnel.

7.4.4 Dispensing

<u>Stage 1B</u>

Study products will be dispensed only to enrolled study participants, or to study staff on behalf of the participant. Twenty individually wrapped pre-filled applicators dispensed in a bag with a zip enclosure will be provided at Visit 2. The participants will be provided with sealable individual bags to store each used applicator plus a larger bag labeled “Used Applicators” into which the individual bags containing used applicators will be collected for return to the clinic. Approximately six weeks later, at Visit 3, participants will return all used and unused applicators. Once the applicators are counted, the participant’s unused applicators will be re-dispensed to the participant along with

additional applicators for a total of 20 applicators. Unused applicators may not be dispensed to another participant.

Stage 2

Study products will be dispensed only to enrolled study participants, or to study staff on behalf of the participant, upon receipt of a written prescription from an authorized prescriber. An authorized prescriber includes the Investigator of Record (IoR) or a licensed clinician directly responsible to the IoR as noted on the FDA 1572.

At Treatment 1 Visit (Visit 7) one individually wrapped pre-filled applicator will be dispensed to study staff in a bag with a zip enclosure to be clinician administered at this visit. At the Treatment 2 Safety Clearance Visit (Visit 9), eight individually wrapped pre-filled applicators will be dispensed to study staff in a bag with a zip enclosure for use at home. Additionally this bag will contain a bag labeled “used applicators.” Depending on randomization, the participant will receive 8 pre-filled applicators containing tenofovir 1% gel or HEC placebo gel.

Participants will be instructed to contact the study site to request additional supplies in the event that additional supplies between visits are needed. All circumstances resulting in this additional supply will be documented fully by the site investigator or designee. The pharmacist will record the dispensing of any additional study product on the documents maintained by the Pharmacist of Record or designee. The participants will be provided with sealable bags to collect and store all used applicators, for return to the clinic.

7.4.5 Accountability

The Pharmacist of Record (PoR) is required to maintain complete records of all study products received and subsequently dispensed.

7.4.6 Retrieval of Used and Unused Study Products

Study participants will be instructed to bring all used and unused study products back to the enrollment site for disposal. In the event that used and unused study products are not returned to the enrollment site, study staff members will make attempts to retrieve used and unused study products.

7.4.7 Assessment of Retrieved Study Products and Participant Adherence

Counts of used and unused applicators will generate data for comparison to data collected via the interactive voice response phone reporting system (PRS) on adherence. Section 11 outlines how data on participant adherence will be incorporated into analysis of the study results.

To monitor adherence, participants will be asked to use the interactive voice response PRS after each episode of gel use. To access the PRS, participants call a toll-free

number, identify themselves to the system using a unique ID number and pin, and then respond to pre-recorded questions on product use, use of other products rectally, whether they had RAI, and if so, whether a condom was used and their partners' reactions to the gel, and whether there is any comment related to this particular occasion of product use. Responses can be entered by either pressing keys (1 for yes, 2 for no) or by voice response that is understood and registered by the system. Participants receive a small monetary incentive for each call regardless of their report of product use or lack of use; furthermore, a bonus at the end of the trial period (either three months or seven days for Stage 1B and Stage 2, respectively) is accrued by those who have not missed any weekly call for Stage 1B or daily call for Stage 2.

Automatic follow-up calls will be made to participants who have not called in either 6 days (144 hours) during Stage 1B or 48 hours in Stage 2. The system will prompt participants to answer the questionnaire. If a participant is not available or does not respond to questions during this follow-up call, the system will ask them to call when available within the next 24 hours. If a call is not received within 24 hours from the time of the follow-up call, the system will send an alert to the site coordinators and staff members at Columbia University via email. The study coordinator at the study site will contact participants to inquire about missed calls (if they forgot to call) and adherence to the study product regimen. Thus, this system allows monitoring of adherence to calling the PRS on a time-stamped basis; the calls will constitute one measure of study product adherence. Self-reports will be cross-validated with applicator counts to assess adherence.

7.5. Concomitant Medications and Procedures

All concomitant medications, including those used to treat AE's, will be recorded in the participant's chart on forms designed for that purpose. Prescription medications, over-the-counter preparations, vitamins and nutritional supplements, and herbal preparations will all be recorded as concomitant medications. Medications/procedures not listed below under precautionary and prohibited medications and procedures are permitted.

7.6. Prohibited Medications and Procedures

Oil-based lubricants are known to damage the integrity of latex male condoms and are not recommended.

<u>Stage 2</u>
Study participants will be prohibited from using the following medications throughout the Stage 2 study period: heparin (including Lovenox®), warfarin, Plavix® (clopidogrel bisulfate), rectally administered medications (including over-the-counter products), aspirin or NSAIDS, and other drugs that are associated with increased likelihood of bleeding following mucosal biopsy.

Following procurement of mucosal biopsies by anoscopy participants will be advised to refrain from RAI or any practices, which include rectal insertion of any product including those used during sexual intercourse (sex toys) for 7 days.

Furthermore, study participants will be advised not to use the following products within 2 weeks or 10 half-lives of the drug, whichever is longer, of Visit 6 and throughout Stage 2 study participation: systemic immunomodulatory medications, rectally administered medications, rectally administered products containing N-9, or any other investigational products. Should participants report use of any of these medications or products, they will be required to discontinue use of study product, but will continue to complete all scheduled study visits.

7.7. Required Medications and Procedures

Latex male condoms are recommended for use during RAI by participants enrolled in this study. Study sites will provide a single brand of lubricated male latex condoms to participants in quantities expected to be sufficient according to study-specific procedures when study product is dispensed. These male condoms will not be impregnated or coated with any type of spermicide. Male condoms will be recommended for all sexual encounters during the study period. In the event that a participant needs additional male condoms between visits, he may request these from study sites at any time. Participants will be provided with a list of approved brands that can be used in place of the study provided condoms to help encourage condom use.

8 STUDY PROCEDURES

An overview of the study visit and evaluations schedule is presented in Appendix I. Presented in this section is additional information on visit-specific study procedures. Detailed instructions to guide and standardize sample collection are provided in Section 8.11 and the study-specific procedures manual. Unless otherwise specified, the laboratory procedures listed in this section are performed at the local study site laboratories.

In addition to any Interim Visits that may occur in accordance with guidance outlined in Section 8.9, the following visits should take place for study participants:

- Stage 1A
  - Screening/Enrollment (Visit 1)
- Stage 1B
  - Screening/Enrollment (Visit 2)
  - 6 week outpatient use of HEC placebo gel
  - Mid-trial Follow-up (Visit 3)
  - 6 week outpatient use of HEC placebo gel
  - Final Follow-up (Visit 4)
- Stage 2
  - Screening (Visit 5)

*may be combined with Visit 6 if less than 28 days between Visit 4 and Visit 5

- Enrollment (Visit 6)
- Treatment 1 (Visit 7)
- Treatment 1 Follow-Up Phone Call (Visit 8)
- Treatment 2 Safety Clearance (Visit 9)
- 1 Week outpatient use of study gel
- Treatment 2 Follow-up (Visit 10)
- Final Follow-Up Phone Call (Visit 11)

8.1. Stage 1A

8.1.1 Screening/Enrollment Visit (Visit 1)

Written informed consent will be obtained before any procedures are initiated. For participants who do not meet the eligibility criteria, screening will be discontinued once ineligibility is determined. All Visit 1 procedures must be completed within 48 hours.

Table 6: Visit 1

Stage 1A Screening/Enrollment Visit	
**Component**	**Procedure/Analysis**
**Administrative**	• Obtain written informed consent<br>• Assign participant ID (PTID)<br>• Collect demographic and locator information<br>• Assess eligibility<br>• Provide reimbursement for study visit
**Behavioral**	• Participant to complete Web-based Baseline Behavioral Questionnaire (BBQ)
**Clinical**	• Collect medical history<br>• Perform physical exam<br>• Perform digital rectal exam and anoscopy<br>• Provide counseling and condoms<br>o Condom use, HIV/STI risk reduction<br>o HIV pre-and post-test
**Oral**	• Collect buccal swab and perform HIV rapid test, if not using blood sample<br>• Collect oral rinse specimen for HPV typing
**Urine**	• Collect urine sample for GC/CT testing by NAAT
**Blood**	• Collect blood specimens for<br>o CBC with differential and platelets<br>o BUN, creatinine, electrolyte panel, ALT, AST<br>o Syphilis rapid plasma reagin (RPR) (confirmatory tests as needed)<br>o HSV serologies (HSV 1 and 2 antibody testing)<br>o Hepatitis B surface antigen test<br>o Hepatitis C antibody test<br>o HIV serostatus, if HIV rapid test positive or using blood instead of buccal sample for HIV rapid test<br>** If HBsAg negative with no history of vaccination, participant will be offered a referral for vaccination<br>• Perform HIV rapid test, if not using buccal sample
**Rectal Specimens**	• Collect rectal swabs for<br>o *Chlamydia trachomatis* and *Neisseria gonorrhea* by NAAT<br>o HPV typing

* HRA will be performed at the Pittsburgh and Boston sites in addition to standard anoscopy (non sex-workers only).

8.2. Stage 1B

8.2.1 Screening/Enrollment Visit (Visit 2)

Visit 2 must take place no more than 28 days following Visit 1; after this period, participants will need to be partially rescreened to ensure that they receive medical clearance. Rescreening will include the following laboratory tests:

- HIV rapid test
- HIV serostatus, if HIV rapid test positive
- Syphilis rapid plasma reagin (RPR) (confirmatory tests as needed)
- Hepatitis B surface antigen test

- BUN, creatinine, electrolyte panel, ALT, AST
- Chlamydia trachomatis and Neisseria gonorrhea by NAAT (rectal only)

For participants who do not meet the eligibility criteria, screening will be discontinued once ineligibility is determined. Participants not meeting the eligibility criteria may be rescreened at a later date, if appropriate, as in the case of a medication washout period. However, individuals deemed ineligible due to sexually transmitted infections of the rectum, will not be rescreened.

Table 7: Visit 2

Stage 1B Day 0: Screening/Enrollment Visit	
**Component**	**Procedure/Analysis**
**Administrative**	• Obtain written informed consent<br>• Confirm/update locator information<br>• Provide any outstanding test results<br>• Confirm eligibility<br>• Provide reimbursement for study visit<br>• Schedule next study visit
**Behavioral**	• Participant to complete video teleconference with "*Alex*"<br>o Interactive voice response PRS use and emails to "*Alex*"
**Clinical**	• Review/update medical history<br>• Record any AEs and concomitant medications<br>• Perform physical exam, if clinically indicated<br>• Perform digital rectal exam and standard anoscopy, if clinically indicated<br>• Provide counseling and condoms<br>o Condom use, HIV/STI risk reduction<br>o HIV pre-and post-test, if clinically indicated
**Oral, if clinically indicated**	• Collect buccal swab and perform HIV rapid test, if not using blood sample
**Urine, if clinically indicated**	• Collect urine sample *for Chlamydia trachomatis* and *Neisseria gonorrhea* by NAAT
**Blood, if clinically indicated**	• Collect blood specimens for<br>o CBC with differential and platelets<br>o PT/INR (non sex-workers only)<br>o BUN, creatinine, electrolyte panel, ALT, AST<br>o Syphilis rapid plasma reagin (RPR) (confirmatory tests as needed)<br>o HSV serologies (HSV 1 and 2 antibody testing)<br>o Hepatitis B surface antigen test**<br>o Hepatitis C antibody test<br>o HIV serostatus, if HIV rapid test positive or using blood instead of buccal sample for HIV rapid test<br>** If HBsAg negative with no history of vaccination, participant will be offered a referral for vaccination<br>• Perform HIV rapid test, if not using buccal sample
**Rectal Specimens, if clinically indicated**	• Collect rectal swabs *for Chlamydia trachomatis* and *Neisseria gonorrhea* by NAAT
**Study Product Supply**	• Dispense 20 doses of HEC universal placebo gel<br>o Educate patient regarding study product use, storage, and return

8.2.2 Six-week outpatient use of HEC placebo gel

Participants will use the HEC placebo gel in the context of RAI and report use via interactive voice response PRS and optional emails to "*Alex*". Participants will place used

applicators in individual bags with a zip enclosure and then place the individual bag into the larger bag labeled "Used Applicators".

8.2.3 Mid-Trial Follow-up (Visit 3)

Table 8: Visit 3

**Stage 1B Day 42 (+/- 7 days): Mid-Trial Follow-up Visit**	
**Component**	**Procedure/Analysis**
**Administrative**	• Confirm informed consent in place<br>• Confirm/update locator information<br>• Provide any outstanding test results<br>• Provide reimbursement for study visit<br>• Schedule next study visit
**Behavioral**	• Interactive voice response PRS use and emails to "*Alex*"
**Clinical**	• Review/update medical history<br>• Record any AEs and concomitant medications<br>• Perform symptom directed physical exam<br>• Perform digital rectal exam and standard anoscopy<br>• Provide counseling and condoms<br>o Condom use, HIV/STI risk reduction<br>o HIV pre-and post-test, if clinically indicated
**Oral, if clinically indicated**	• Collect buccal swab and perform HIV rapid test
**Urine**	• Collect urine sample *for Chlamydia trachomatis* and *Neisseria gonorrhea* by NAAT
**Blood, if clinically indicated**	• Collect blood specimens for<br>o CBC with differential and platelets<br>o BUN, creatinine, electrolyte panel, ALT, AST<br>o Syphilis rapid plasma reagin (RPR) (confirmatory tests as needed)<br>o Hepatitis B surface antigen test**<br>o HIV serostatus, if HIV rapid test positive or using blood instead of buccal sample for HIV rapid test<br>** If HBsAg negative with no history of vaccination, participant will be offered a referral for vaccination<br>• Perform HIV rapid test
**Rectal Specimens**	• Collect rectal swabs *for Chlamydia trachomatis* and *Neisseria gonorrhea* by NAAT
**Study Product Supply**	• Collect used and unused applicators<br>• Dispense up to 20 doses of HEC universal placebo gel<br>o Educate patient regarding study product use, storage, and return

8.2.4 Six-week outpatient use of HEC placebo gel

Participants will use the HEC placebo gel in the context of RAI and report on use via interactive voice response PRS and optional emails to "*Alex*". Participants will place used applicators in a bag with a zip enclosure labeled "Used Applicators".

8.2.5 Final Follow-Up (Visit 4)

Table 9: Visit 4

**Stage 1B Day 84 (+/- 7 days): Final Follow-up Visit**	
**Component**	**Procedure/Analysis**
**Administrative**	• Confirm informed consent in place<br>• Confirm/update locator information<br>• Provide test results<br>• Provide reimbursement for study visit
**Behavioral**	• Participant to complete Web-based PAQ and SBQ<br>• Participant to complete video teleconference with "*Alex*"*<br>o Discuss acceptability, adherence, and research technology
**Clinical**	• Review/update medical history<br>• Record any AEs and concomitant medications<br>• Perform physical exam<br>• Perform digital rectal exam and standard anoscopy<br>• Provide counseling and condoms<br>o Condom use, HIV/STI risk reduction<br>o HIV pre-and post-test
**Oral**	• Collect buccal swab and perform HIV rapid test, if not using blood sample<br>• Collect oral rinse specimen for HPV typing
**Urine**	• Collect urine sample for *Chlamydia trachomatis* and *Neisseria gonorrhea* by NAAT
**Blood**	• Collect blood specimens for<br>o CBC with differential and platelets<br>o BUN, creatinine, electrolyte panel, ALT, AST<br>o Syphilis rapid plasma reagin (RPR) (confirmatory tests as needed)<br>o Hepatitis B surface antigen test**<br>o HIV serostatus, if HIV rapid test positive or using blood instead of buccal sample for HIV rapid test<br>** If HBsAg negative with no history of vaccination, participant will be offered a referral for vaccination<br>• Perform HIV rapid test, if not using buccal sample
**Rectal Specimens**	• Collect rectal swabs for<br>o *Chlamydia trachomatis* and *Neisseria gonorrhea* by NAAT<br>o HPV typing
**Study Product Supply**	• Collect used and unused applicators

*Video teleconference must take place within 72 hours of Visit 4 clinical assessments

8.3. Stage 2

8.3.1 Screening (Visit 5)

Written informed consent will be obtained before any screening procedures are initiated. For participants who do not meet the eligibility criteria, screening will be discontinued once ineligibility is determined. Participants not meeting the eligibility criteria may be rescreened at a later date, if appropriate, as in the case of a medication washout period.

Table 10: Visit 5

Stage 2: Screening Visit*<br>* If has current (i.e., within last 28 days) laboratory results, physical exam, and anoscopy on file at the site, these assessments do not need to be repeated.	
**Component**	**Procedure/Analysis**
**Administrative**	• Obtain written informed consent<br>• Assess eligibility<br>• Obtain locator information<br>• Provide reimbursement for study visit<br>• Schedule next study visit
**Clinical**	• Review/update medical history<br>• Record any AEs and concomitant medications<br>• Perform physical exam<br>• Perform digital rectal exam and standard anoscopy<br>• Provide counseling and condoms<br>o Condom use, HIV/STI risk reduction<br>o HIV pre-and post-test
**Oral**	• Collect buccal swab and perform HIV rapid test, if not using blood sample
**Urine**	• Collect urine sample for *Chlamydia trachomatis* and *Neisseria gonorrhea* by NAAT
**Blood**	• Collect blood specimens for<br>o CBC with differential and platelets<br>o PT/INR (if more than 6 months since last PT/INR)<br>o BUN, creatinine, electrolyte panel, ALT, AST<br>o Syphilis rapid plasma reagin (RPR) (confirmatory tests as needed)<br>o Hepatitis B surface antigen test**<br>o HSV serologies (HSV 1 and 2 antibody testing)<br>o HIV serostatus, if HIV rapid test positive or using blood instead of buccal sample for HIV rapid test<br>** If HBsAg negative with no history of vaccination, participant will be offered a referral for vaccination<br>• Perform HIV rapid test, if not using buccal sample
**Rectal Specimens**	• Collect rectal swabs for<br>o *Chlamydia trachomatis* and *Neisseria gonorrhea* by NAAT

8.3.2 Enrollment (Visit 6)

Visit 6 can be combined with Visit 5, if eligibility can be determined based on current (i.e., within last 28 days) laboratory results, physical exam, and anoscopy on file at the site from Stage 1AB.

Table 11: Visit 6

Stage 2 Day 0: Enrollment Visit	
**Component**	**Procedure/Analysis**
**Administrative**	• Confirm informed consent in place<br>• Confirm/update locator information<br>• Provide test results<br>• Complete randomization<br>• Provide reimbursement for study visit<br>• Schedule next study visit
**Clinical**	• Review/update medical history<br>• Record any AEs and concomitant medications<br>• Perform symptom directed physical exam<br>• Perform digital rectal exam and anoscopy<br>• Provide counseling and condoms<br>o Condom use, HIV/STI risk reduction<br>o HIV pre-and post-test
**Oral, if clinically indicated**	• Collect buccal swab and perform HIV rapid test, if not using blood sample
**Urine, if clinically indicated**	• Collect urine sample for *Chlamydia trachomatis* and *Neisseria gonorrhea* by NAAT
**Blood, if clinically indicated**	• Collect blood specimens for<br>o CBC with differential and platelets<br>o BUN, creatinine, electrolyte panel, ALT, AST<br>o Syphilis rapid plasma reagin (RPR) (confirmatory tests as needed)<br>o Hepatitis B surface antigen test**<br>o HIV serostatus, if HIV rapid test positive or using blood instead of buccal sample for HIV rapid test<br>** If HBsAg negative with no history of vaccination, participant will be offered a referral for vaccination<br>• Perform HIV rapid test, if not using buccal sample
**Rectal Specimens**	• Collect rectal swabs for GC/CT by NAAT<br>• Collect rectal sponges for cytokine profile by Luminex®<br>• Collect anorectal biopsies (~5) for<br>o Histopathology<br>o Mononuclear cell phenotype<br>o Cytokine mRNA profile

8.3.3 Treatment 1 (Visit 7)

Visit 7 will occur 7 to 28 days after Visit 6.

Table 12: Visit 7

**Stage 2 Day 7-28: Treatment 1 Visit**	
**Component**	**Procedure/Analysis**
**Administrative**	• Confirm informed consent in place<br>• Confirm/update locator information<br>• Provide test results<br>• Complete randomization<br>• Provide reimbursement for study visit<br>• Schedule next study visit
**Clinical**	• Review/update medical history<br>• Record any AEs and concomitant medications<br>• Perform symptom directed physical exam<br>• Perform digital rectal exam and anoscopy<br>• Provide counseling and condoms<br>o Condom use, HIV/STI risk reduction<br>o HIV pre-and post-test
**Oral, if clinically indicated**	• Collect buccal swab and perform HIV rapid test, if not using blood sample
**Urine, if clinically indicated**	• Collect urine sample for GC/CT by NAAT
**Blood, if clinically indicated**	• Collect blood specimens for<br>o CBC with differential and platelets<br>o BUN, creatinine, electrolyte panel, ALT, AST<br>o Syphilis rapid plasma reagin (RPR) (confirmatory tests as needed)<br>o Hepatitis B surface antigen test**<br>o HIV serostatus, if HIV rapid test positive or using blood instead of buccal sample for HIV rapid test<br>** If HBsAg negative with no history of vaccination, participant will be offered a referral for vaccination<br>• Perform HIV rapid test, if not using buccal sample
**Rectal Specimens**	• Collect rectal swabs for GC/CT by NAAT, if clinically indicated<br>• Collect rectal sponges for cytokine profile by Luminex®<br>• Collect anorectal biopsies (~5) for<br>o Histopathology<br>o Mononuclear cell phenotype<br>o Cytokine mRNA profile
**Study Product Supply**	• Administer single dose of study product rectally

8.3.4 Treatment 1 Follow-Up Phone Call (Visit 8)

A follow-up phone will be scheduled to take place within 24 hours of the Treatment 1 Visit (Visit 7). Study staff will follow-up with participants to inquire about AEs they might experience as a result of study product or procedures performed during the Treatment 1 Visit (Visit 7).

Table 13: Visit 8

**Stage 2: Treatment 1 Follow-up Phone Call**	
**Component**	**Procedures/Analysis**
**Clinical**	• Record any AEs and concomitant medications

8.3.5 Treatment 2 Safety Clearance (Visit 9)

Visit 9 will occur 7 to 21 days after Treatment 1 Visit (Visit 7).

Table 14: Visit 9

**Stage 2 Day 14-49: Treatment 2 Safety Clearance Visit**	
**Component**	**Procedure/Analysis**
**Administrative**	• Confirm informed consent in place<br>• Confirm/update locator information<br>• Provide test results<br>• Provide reimbursement for study visit<br>• Schedule next study visit
**Clinical**	• Review/update medical history<br>• Record any AEs and concomitant medications<br>• Perform symptom directed physical exam<br>• Perform digital rectal exam and anoscopy<br>• Provide counseling and condoms<br>o Condom use, HIV/STI risk reduction<br>o HIV pre-and post-test
**Behavioral**	• Participant to complete video teleconference with "*Alex*"<br>o Interactive voice response PRS use and emails to "*Alex*"
**Oral, if clinically indicated**	• Collect buccal swab and perform HIV rapid test, if not using blood sample
**Urine, if clinically indicated**	• Collect urine sample for GC/CT by NAAT
**Blood, if clinically indicated**	• Collect blood specimens for<br>o CBC with differential and platelets<br>o BUN, creatinine, electrolyte panel, ALT, AST<br>o Syphilis rapid plasma reagin (RPR) (confirmatory tests as needed)<br>o Hepatitis B surface antigen test**<br>o HIV serostatus, if HIV rapid test positive or using blood instead of buccal sample for HIV rapid test<br>** If HBsAg negative with no history of vaccination, participant will be offered a referral for vaccination<br>• Perform HIV rapid test, if not using buccal sample
**Rectal Specimens, if clinically indicated**	• Collect rectal swabs for GC/CT by NAAT
**Study Product Supply**	• Dispense 8 doses of study product<br>• Explain study product use, storage, and return

8.3.6 One-Week Outpatient Use of Study Product

Initiation of outpatient study product use must occur within 7 days of the Treatment 2 Safety Clearance Visit (Visit 9). Participants will use the study product once daily for 7 consecutive days and report use via interactive voice response PRS and optional emails

to "*Alex*". Participants will place used applicators in individual bags with a zip enclosure and then place the individual bag into the larger bag labeled "Used Applicators". A follow-up phone will be scheduled to take place on the fourth day of daily dosing. Study staff will follow-up with participants to inquire about AEs they might experience as a result of study product or procedures performed during the Visit 9.

8.3.7 Treatment 2 Follow-up/Final Clinic Visit (Visit 10)

Visit 10 will occur 7-14 days after Visit 9 on the day after the last dose of study product.

Table 15: Visit 10

**Stage 2 Day 21-56: Treatment 2 Follow-up Visit**	
**Component**	**Procedure/Analysis**
**Administrative**	• Confirm informed consent in place<br>• Confirm/update locator information<br>• Provide test results<br>• Provide reimbursement for study visit<br>• Schedule final follow-up phone call
**Behavioral**	• Participant to complete Web-based PAQ and SBQ<br>• Participant to complete video teleconference with "*Alex*"*<br>o Discuss acceptability, adherence, and research technology
**Clinical**	• Review/update medical history<br>• Record any AEs and concomitant medications<br>• Perform symptom directed physical exam<br>• Perform digital rectal exam and anoscopy<br>• Provide counseling and condoms<br>o Condom use, HIV/STI risk reduction<br>o HIV pre-and post-test
**Oral**	• Collect buccal swab and perform HIV rapid test, if not using blood sample
**Urine**	• Collect urine sample *for* GC/CT by NAAT
**Blood**	• Collect blood specimens for<br>o CBC with differential and platelets<br>o BUN, creatinine, electrolyte panel, ALT, AST<br>o Syphilis rapid plasma reagin (RPR) (confirmatory tests as needed)<br>o Hepatitis B surface antigen test**<br>o HIV serostatus, if HIV rapid test positive or using blood instead of buccal sample for HIV rapid test<br>** If HBsAg negative with no history of vaccination, participant will be offered a referral for vaccination<br>• Perform HIV rapid test, if not using buccal sample
**Rectal Specimens**	• Collect rectal swabs for GC/CT by NAAT<br>• Collect rectal sponges for cytokine profile by Luminex®<br>• Collect anorectal biopsies (~5) for<br>o Histopathology<br>o Mononuclear cell phenotype<br>o Cytokine mRNA profile
**Study Product Supply**	• Collect used and unused applicators

*Video teleconference must take place within 72 hours of Visit 10 clinical assessments

8.3.8 Final Follow-Up Phone Call (Visit 11)

A follow-up phone will be scheduled to take place within 24 hours of the Visit 10. Study staff will follow-up with participants to inquire about AEs they might experience as a result of study product or procedures performed during the Visit 10. If all clinical laboratory test results are not available at the time of the follow-up call, another call will be made once the results are available (See Section 8.9).

Table 16: Visit 11

**Stage 2: Final Follow-up Phone Call**	
**Component**	**Procedures/Analysis**
**Clinical**	• Provide test results<br>• Record any AEs and concomitant medications

8.4. Follow-up Procedures for Participants Who Discontinue Study Product

Participants who temporarily or permanently discontinue study product will not routinely be withdrawn from the study. Rather, every effort will be made to complete all protocol-specified visits and procedures for that stage with these participants with the exceptions described below.

8.5. Participants Who Become Infected with HIV

Study staff will capture seroconversions on study case report forms (CRFs). Protocol-specified procedures will continue until the current stage is completed except:

- HIV serology
- Provision of study product
- Adherence counseling (protocol and product use)
- Counseling for HIV/STI risk reduction. Counseling will be modified to address primary and secondary HIV/STI prevention for infected individuals.
- Anoscopy
- Anorectal biopsies

8.6. Participants Who Voluntarily Discontinue Study Product

If the participant does not withdraw from the study, all protocol-specified study procedures will continue until the current stage is completed except:

- Provision of study product
- Adherence counseling (protocol and product use)
- Anoscopy
- Anorectal biopsies

8.7. Participants Who Are Discontinued from Study Product by the Site Investigator

If the participant is not withdrawn from the study, all protocol-specified study procedures will continue until the current stage is completed except:

- Provision of study product
- Adherence counseling (protocol and product us)
- Anoscopy
- Anorectal biopsies

8.8. Participants Who Withdraw or Are Withdrawn from the Study

If the participant withdraws or is withdrawn from the study, an Early Termination Visit will be conducted, if possible.

Table 17: Early Termination Visit

**Stage 1B or Stage 2: Early Termination Visit**<br>The following procedures will be performed as clinically indicated	
**Component**	**Procedure/Analysis**
**Administrative**	• Confirm informed consent in place<br>• Confirm/update locator information<br>• Provide test results<br>• Provide reimbursement for study visit
**Behavioral**	• Participant to complete Web-based Product Acceptability Questionnaire (PAQ) and Study Burden Questionnaire (SBQ), if applicable<br>• Participant to complete interactive voice response PRS use and emails to "*Alex*", if applicable<br>• Participant to complete video teleconference with "*Alex*", if applicable<br>o Discuss acceptability, adherence, and research technology
**Clinical**	• Review/update medical history<br>• Record any AEs and concomitant medications<br>• Perform physical exam<br>• Perform digital rectal exam and standard anoscopy<br>• Provide counseling and condoms<br>o Condom use, HIV/STI risk reduction<br>o HIV pre-and post-test
**Oral**	• Collect buccal swab and perform HIV rapid test, if not using blood sample<br>Terminates during Stage 1B<br>• Collect oral rinse specimen for HPV typing
**Urine**	• Collect urine sample for GC/CT by NAAT
**Blood**	• Collect blood specimens for<br>o CBC with differential and platelets<br>o BUN, creatinine, electrolyte panel, ALT, AST<br>o Syphilis rapid plasma reagin (RPR) (confirmatory tests as needed)<br>o Hepatitis B surface antigen test**<br>o HIV serostatus, if HIV rapid test positive or using blood instead of buccal sample for HIV rapid test<br>** If HBsAg negative with no history of vaccination, participant will be offered a referral for vaccination<br>• Perform HIV rapid test, if not using buccal sample
**Rectal Specimens**	Terminates during Stage 1B<br>• Collect rectal swabs for<br>o GC/CT by NAAT<br>o HPV typing<br>Terminates during Stage 2 after using study product<br>• Collect rectal swabs for GC/CT by NAAT<br>• Collect rectal sponges for cytokine profile by Luminex®<br>• Collect anorectal biopsies (~5) for<br>o Histopathology<br>o Mononuclear cell phenotype<br>o Cytokine mRNA profile
**Study Product Supply**	• Collect used and unused applicators, if applicable

8.9. Interim Contacts and Visits

Interim visits may be performed at any time during the study. All interim contacts and visits will be documented in participants' study records and on applicable case report forms.

Some Interim Visits may occur for administrative reasons. For example the participant may have questions for study staff. Other interim contacts and visits may occur in response to AEs experienced by study participants. When interim contacts or visits are completed in response to participant reports of AEs, study staff will assess the reported event clinically and provide or refer the participant to appropriate medical care.

Table 18: Interim Contacts and Visits

Stage 1B or Stage 2: Interim Visit	
**Component**	**Procedure/Analysis**
**Administrative**	• Review/update demographic information<br>• Review/update locator information<br>• Provide test results, if applicable<br>• Schedule next visit, if applicable
**Clinical**	• Review/update medical history<br>• Record any AEs and concomitant medications<br>• Perform symptom directed physical exam, if clinically indicated<br>• Perform digital rectal exam and standard anoscopy, if clinically indicated<br>• Reinforce counseling<br>o Condom use, HIV/STI risk reduction<br>o HIV pre-and post-test counseling
**Specimens**	• Collect appropriate specimens and perform testing as clinically indicated
**Study Product**	• Return used/unused study product

8.10. Final Contact

The Final Clinic Visit for all participants will include clinical laboratory testing. A final contact may be required to provide these study test results and post-test counseling. All final contacts will be documented in participant study records.

8.11. Clinical Evaluations and Procedures

The following history and physical exam components will be conducted at select visits.

Medical History

- Each participant will be asked about any symptoms or AEs experienced since their previous visit

Physical Exam

- Height (may be omitted after the Stage 1A Visit)
- Weight (may be omitted after the Stage 1A Visit)
- Vital signs

  - Temperature
  - Pulse
  - Blood pressure
- General appearance
- Abdomen
- Other components as indicated by participant symptoms

<u>Rectal Exam and Rectal Specimen Collection</u>

The participant will be positioned in the left lateral decubitus position for the following procedures:

<u>Rectal Specimen Collection Before Rectal Exam</u>

- Rectal swab collection for GC/CT: The anal margin will be gently everted and swabs blindly inserted 1 inch proximal to the anal margin, turned through 360 degrees and withdrawn.
- Rectal swab for HPV: The examiner will place a water moistened Dacron swab 2 inches proximal to the anal margin. Then with lateral pressure and a spiral motion he/she will slowly remove the swab over 10 seconds.

<u>Rectal Exam</u>

- Visual and digital rectal exam: The examiner will conduct a visual examination of the anus and surrounding area and note any abnormality. The examiner will then insert a lubricated gloved finger into the anal canal and sweep around the internal anal circumference.
- Standard anoscopy: A lubricated plastic anoscope will be gently and fully inserted (until the lateral ‘wings’ touch the anal margin) and the obturator removed. The anal and rectal mucosa will be visually inspected by the clinician’s naked eye.
- High-resolution anoscopy: After standard anoscopy is performed, a high-resolution anoscopic assessment will be made of the anorectal epithelium. Images will be captured from the rectum, anorectal transition zone, anal canal, and perianal area using Second Opinion Software (Second Opinion Software, LLC, Torrance, CA) and linked with a written description of the clinical findings reported by the site physician. Dr. Ross Cranston and Dr. Lori Panther will review all the Pittsburgh and Boston images to determine the presence or absence of anorectal pathology. They will share 10% of all images taken to assess similarity of assessments between sites.

<u>Rectal Specimen Collection After Digital Rectal Exam</u>

- Rectal sponge collection for cytokines: The sponge is inserted through the anoscope and placed in contact with rectum and remain there for 5 minutes. The sponge will then be removed and packaged, then the anoscope will be slowly removed
- Anoscopic biopsy: A lubricated anoscope will be inserted into the anorectum until the ‘wings’ touch the anal verge. Biopsies will be taken at approximately 9 cm using biopsy forceps

8.12. Behavioral Measures

8.12.1 Stage 1AB

The behavioral measures used in this protocol include:

Baseline Behavioral Questionnaire (BBQ)

Participants will complete a web-based self-interview at the Stage 1A Screening Visit that will start with an introductory page featuring a friendly picture of the research assistant (*"Alex"*) and some welcome-to-the-study remarks.

Table 19. Baseline Behavioral Questionnaire

Title	Content and rationale for inclusion
Demographics	Age, educational level, ethnicity, sexual identity, employment status and income will be used for sample description.
Sexual Behavior (prior 3 months)	Participants 1) habitual sexual behavior with HIV-negative, positive, or unknown status men and women, 2) sexual roles -- insertive, receptive, versatile, 3) frequency of condom use and 4) partner type (lover, one-night stand, other) or (client, non-commercial partner for sex workers). This will allow understanding of the participant's habitual sexual behavior and circumstances in which microbicide use needs to fit.
Douching (hygienic practices prior 3 months)	Frequency of enema use, purpose, enema use prior and after sex, injuries resulting from douching, and side effects. This will allow understanding of degree of similarity between habitual practices and study requirements, and how this may affect acceptability.
Lubricant Use (prior 3 months)	Types of rectal lubricants (e.g., silicone, oil or water based) participant's use, preferences, amount used, and mode of application. This will allow understanding of possible preexisting preferences and how they may affect attitudes about different formulations of study gel.
Substance Use (prior 3 months)	This will identify what recreational drugs were used in conjunction with sex at the individual uses. Viagra and other sexual enhancing drugs will also be included.
HIV Testing	By eligibility criteria, all participants will be HIV-negative by self-report. This will assess frequency of HIV testing and latest test occasion prior to enrollment in the study.
Likelihood of Rectal Microbicide Use	This section will identify likelihood of microbicide gel use before RAI with different types of partners and when using substances. This will identify pre-trial attitudes about such product and how they may affect later ratings.
PEP/PREP	Given that some consideration is currently being given to microbicides that could be taken as pills, either in anticipation of possible HIV exposure or after it, this section will assess knowledge about PEP/PREP likelihood of use.
**Additional Sections for Sex Worker Cohort Only**	
Sex Work/Victimization	This section assesses the length of time involved in sex work, reasons for getting involved, reasons continues to engage, how clients are met, abuse experienced while engaging in sex work or because of presumed status as gay, transgender, or sex worker, post-abuse follow-up care.
Prioritization of HIV-prevention/Risk perception/Motivation to Remain HIV-uninfected	This section measures the perceived likelihood of getting HIV or an STD in the future and the most important issues when about to have sex, and level of motivation to avoid getting HIV.
Perceived Effectiveness and Difficulty of Discussing the HIV test with Clients	This section addresses participant's perceived comfort-level under different circumstances of using a rapid HIV home test with clients.
Intentions to use a Rapid HIV-home test	Likelihood of using the rapid HIV home test on oneself or with clients, and amount participant would be willing to pay for each test.

Adherence Questionnaire (AQ)

Adherence will be assessed by an interactive voice response phone reporting system (PRS) to which participants will be asked to call in after each product use in Stage 1B and Stage 2. Participants will keep tallies of their sexual behavior by calling the PRS with voice commands recorded in *Alex's* voice. They will be encouraged to call as soon as possible following a sexual encounter but not less than once a week, regardless of whether a sexual encounter took place. Modest monetary incentives will be used to encourage regular calls. The phone system will be used to report each sexual encounter, occurrence of RAI, condom use, and study product use. This information will be cross-validated with used/unused returned applicator counts.

Product Acceptability Questionnaire (PAQ)

This web-based self-interview will be completed by participants at the end of Stage 1B and at the end of Stage 2.

Table 20: Product Acceptability Questionnaire

Factor	Title:	Content
**Vehicle associated**	Product acceptability	Likes and dislikes of physical characteristics of the product and sensations
**Application associated**	Application process	Ease of application, position, timing before intercourse
	Applicator	Likes and dislikes about applicator, its tip, and its portability
**Use associated**	Changes in hygiene due to product use	Will inquire about changes in douching practices concurrent with gel use and reasons for the change
	Experiences using the product	Leakage, soiling, or other product side effects, and how much the participant was bothered by them; feeling that the product was absorbed in the intestine
**Related covariates**	Partner's reaction	Type of partner with whom product was used, type of relationship, emotional closeness/lack of, length of relationship, disclosure of product use to partner, partner's reaction/product opinion
	Sexual enjoyment after product use	Respondent and partner's enjoyment of sex after product use as compared to usual sexual experience
	Condoms	Attitudes about condom use during the trial, if applicable
	Changes in sexual practices due to product use	Ease of penetration, interruption of sex, attitudes about it
	Possibility of covert use	Possibility of non-disclosure of product use to different types of partners
**Likelihood of use**	Likelihood of product use in the future	Likelihood of using a product that contributes to providing some protection against HIV; likelihood of using it when condoms are not used, if wait period were required, and according to its cost.
	Willingness to use a higher volume	Given that the volume of a rectal microbicide needed to convey protection has not been agreed upon yet, this will explore participant's willingness to use more (or less) than the volume used in the study.
	Recommendations	Suggested changes to the product

Study Burden Questionnaire (SBQ)

This questionnaire will be administered at the end of Stage 1B and the end of Stage 2. The SBQ will explore through structured questions the participant's overall experiences

during the trial, his likes and dislikes, burden due to study procedures, and suggestions for future trials.

<u>Video Teleconferences (VTC)</u>

The teleconferences will be conducted via the Internet using a webcam at the Visits 2, 4, 9, and 10. The interview will follow an IRB-approved guide, but will be handled by the interviewer ("*Alex*") in a flexible manner, following the natural flow of the information presented by the interviewee. New topics are likely to appear in each interview, which may be incorporated into subsequent interviews for further exploration. Two teleconferences are planned:

- Pre-trial teleconference (Visit 2 and Visit 9)

  The live meeting via the Internet will serve as an introduction between the interviewer and participant to establish initial rapport. The main purpose of this initial contact is to personalize the experience for the participant and walk participants through all of the steps and procedures for this trial. Participants will receive information about the phone reporting system and possibility of sending email messages, as well as a calendar of events outlining all of the procedures.

- End-trial teleconference (Visit 4 and Visit 10)

  Through open-ended questions, the interviewer will explore which aspects of the gel, application method, and applicator the participant liked most and least and which he would change, particularly concerning gel use with RAI. Questions will also be asked to review and explore phone reports. The interview guide will include the following topics:

  - Gel use: What it was like for participants to use the gel during the past three months/week?
  - Problems: Did participants experience any problems? Describe.
  - Rectal Practices: Did participants use any douches or products rectally? Describe.
  - RAI: What it was like to have RAI with the gel?
  - Partner's reaction: What characterizes the relationship of participant with his sexual partner(s), what level of emotional closeness (or lack of it) exists, and how did these factors influence the use of the gel?
  - Likelihood of microbicide use in the future: How likely would participant be to use the gel in the future based on gel use in the study in comparison with other products?

  With the sex worker cohort, the following will also be explored:

  - Sex work environment: how using non-condom based HIV-prevention strategies may fit in with sex work
  - RAI with clients: What was it like using the placebo gel with clients?
  - Likelihood of use of non-condom based HIV prevention strategies: How likely would participant be to use microbicide gel, rapid HIV home test with clients, or PrEP?

These topics and related questions are meant to be openings for the discussion of factors (vehicle associated, application associated, use associated, or otherwise related factors) that will complement the data collected quantitatively.

Besides product use related issues, interviewers will ask how the participant felt about the research technology used:

- Computer, Internet, and email use
- Phone Reporting System
- Teleconferencing
- Study participation

Email messages

Participants will have the option to send email messages to Alex as a complement to the phone reports. The addition of email messages to the data collected through the automated phone reporting system will result in enriched records of participants' experiences with the gel. Use of an anonymous email account will be possible, if participants so choose. "*Alex*" will assist participants in setting up the account using the participant ID number (e.g., TellAlex1001@gmail.com) and PIN. Participants will be free to write as lengthy an email as they want on any topic. Participants will be alerted that "*Alex*" will not be answering the messages as they arrive, but that they will be discussed at the next teleconference. This is done so that the participant is aware that the email message cannot be used for issues that require immediate attention like the reporting of adverse events. If, despite all these warnings, the participant were to report an adverse event via email messages to "*Alex*", "*Alex*" will inform the study site. Furthermore, the following auto reply message will be sent in order to prevent any miscommunication.

If you experience any physical problems or reactions like rashes, fever, bleeding or other physical symptoms,<br><br>OR<br><br>If you experience any emotional distress like severe anxiety, depression, nervousness, despair, or other emotional symptoms,<br><br>YOU MUST CONTACT THE STUDY COORDINATOR<br>AT YOUR LOCAL CLINIC SITE<br>to arrange for ANY follow-up care, whether or not any of these are related to the use of the gel.<br><br>We will not be able to routinely communicate anything you tell us here to your clinical site of care.<br><br>THIS EMAIL ACCOUNT IS FOR RESEARCH PURPOSES ONLY AND NOT TO BE USED FOR CLINICAL CARE.

8.13. Laboratory Evaluations

8.13.1 Local Laboratory Testing

The local laboratory or designee will run the following, as indicated:

- CBC with platelets and differential
- PT/INR
- BUN, creatinine, AST, ALT
- HSV serology (HSV 1 and 2)
- Hepatitis B surface antigen test
- Hepatitis C antibody test
- Syphilis testing by RPR with confirmatory testing as needed
- HIV Rapid Test
- HIV-1 serology, with confirmatory testing as needed
- Urine GC/CT by NAAT

8.13.2 Research Laboratory Testing

The following specimens will be collected and stored locally until shipped for analysis by the McGowan Research Laboratory.

- Oral Rinse Specimens:
  - HPV by PCR
- Rectal swabs:
  - HPV by PCR
- Rectal sponge for cytokines (Luminex)
- Rectal biopsies:
  - Cytokines (RT PCR)
  - Phenotyping (flow cytometry)
  - Histology

The following specimens will be collected and stored locally until shipped for analysis by the Hillier Research Laboratory.

- Rectal swabs:
  - GC/CT by NAAT *Pittsburgh and Puerto Rico sites

The following specimens will be collected and stored locally until shipped for analysis by the Quest Diagnostics Laboratory.

- Rectal swabs:
  - GC/CT by NAAT *Boston site

8.13.3 Specimen Collection and Processing

Each study site will adhere to the standards of good clinical laboratory practice and site standard operating procedures for proper collection, processing, labeling, transport, and storage of specimens at the local laboratory. Specimen collection, testing, and storage at the site laboratories will be documented. In cases where laboratory results are not available due to administrative or laboratory error, sites are permitted to re-draw specimens.

8.13.4 Specimen Handling

Specimens will be handled in accordance with standard laboratory procedures.

8.13.5 Storage of Specimens for Future Use

The mucosal biopsy samples will be processed for histology, cell isolation and flow cytometry, and RNA isolation. Histology blocks will be stored at the McGowan Laboratory in Pittsburgh. The cells isolated from the gut biopsies will be consumed by the flow cytometry process and there will be no residual cells. The RNA will be used for RT-PCR amplification in Pittsburgh. It is anticipated that there will be residual RNA and plasma archive specimens stored in Pittsburgh. After all protocol testing is complete, any residual samples will be stored based on initial consent from the participant. If the participant did not give consent to store samples after completion of the study, each site will discard specimens according to institute policy. After all protocol testing is complete each site will discard all other specimens according to Institute policy.

8.13.6 Biohazard Containment

As the transmission of HIV and other blood-borne pathogens can occur through contact with contaminated needles, blood, and blood products, appropriate blood and secretion precautions will be employed by all personnel in the drawing of blood and shipping and handling of all specimens for this study as recommended by the CDC and NIH. All biological specimens will be transported using packaging mandated by US Code of Federal Regulations (CFR) 42 Part 72. All dangerous goods materials, including diagnostic specimens and infectious substances, must be transported according to instructions detailed in the International Air Transport Association (IATA) Dangerous Goods Regulations. Biohazardous waste will be contained according to institutional, transportation/carrier, and all other applicable regulations.

9 ASSESSMENT OF SAFETY

9.1. Safety Monitoring

The study site investigators are responsible for continuous close safety monitoring of all study participants, and for alerting the protocol team if unexpected concerns arise.

9.2. Clinical Data Safety Review

The study site investigators are responsible for the initial evaluation and reporting of safety information at the participant level and for alerting Dr. McGowan if unexpected concerns arise. If at any time, a decision is made to discontinue study gel in all participants**,** CONRAD, after consultation with the protocol team will inform the US Food and Drug Administration (FDA). The site PIs will notify the responsible IRBs expeditiously.

9.3. Adverse Events Definitions and Reporting Requirements

The Principal Investigators will be responsible for providing monthly summaries of related AEs to CONRAD, as well as periodic AE summaries to the NICHD Program Officer upon request.

9.3.1 Adverse Events

An adverse event is defined as any untoward medical occurrence in a clinical research participant administered an investigational product and which does not necessarily have a causal relationship with the investigational product. As such, an AE can be an unfavorable or unintended sign (including an abnormal laboratory finding, for example), symptom or disease temporally associated with the use of an investigational product, whether or not considered related to the product.

This definition is applied to all participants in Stage 1B and those continuing onto Stage 2 from the time study product (i.e., HEC placebo gel or tenofovir 1% gel) is dispensed in Stage 1B. The term “investigational product” for this study refers to both study products listed in Section 7 plus the gel applicator.

Study participants will be instructed to contact the study site staff to report any AEs they may experience at any time between enrollment and completion of their participation. In the case of a life-threatening event, they will be instructed to seek immediate emergency care. Where feasible and medically appropriate, participants will be encouraged to seek evaluation where the study clinician is based, and to request that the clinician be contacted upon their arrival. Sites will obtain written permission from the participant to obtain and use records from non-study medical providers to complete any missing data element on a CRF related to an adverse event. All participants reporting an untoward medical occurrence will be followed clinically, until the occurrence resolves (returns to baseline) or stabilizes.

The site investigators will determine AE resolution or stabilization in their best clinical judgment, but may seek medical consultation from Dr. McGowan regarding follow up or additional evaluations of an AE. Study site staff will document in source documents and on case report forms all AEs reported by or observed in study participants enrolled in Stage 1B and Stage 2 regardless of severity and presumed relationship to study product.

Participants will be encouraged to report to the study clinician any problems experienced by their partners that might be potentially related to study product. If any such problems

are reported, study staff should evaluate and document the occurrence. Should any concerns arise with regard to partner safety, Dr. McGowan will advise all study sites on appropriate action.

Grading Severity of Events

The Division of AIDS Table for Grading the Severity of Adult and Pediatric Adverse Events, Version 1.0, December 2004, Addenda 3 (Rectal Grading Tables for Use in Microbicide Studies) will be the primary tool for grading adverse events for this protocol. Adverse events not included in Addenda 3 will be graded by the DAIDS AE Grading Table Version 1.0, December 2004. In cases where an AE is covered in multiple tables, Addendum 3 (Rectal Grading Table for Use in Microbicide Studies) will be the grading scale utilized.

The Division of AIDS Table for Grading the Severity of Adult and Pediatric Adverse Events (DAIDS AE Grading Table), Version 1.0, December 2004, and Addenda 3 are available online at http://rcc.tech-res.com/safetyandpharmacovigilance/.

9.3.2 Adverse Event Relationship to Study Product

The relationship of all AEs to study product will be assessed according to the information provided in the tenofovir gel investigator’s brochure, the placebo gel investigator’s brochure, and clinical judgment.

- *Related*: There is a reasonable possibility that the AE may be related to the study agent(s)

- *Not related*: There is not a reasonable possibility that the AE is related to the study agent(s)

9.3.3 Serious Adverse Events

Serious adverse events (SAEs) will be defined as AEs occurring at any dose that:

- Results in death
- Is life-threatening
- Results in persistent or significant disability/incapacity
- Requires inpatient hospitalization or prolongation of existing hospitalization

  Note: Per ICH SAE definition, hospitalization itself is not an adverse event, but is an outcome of the event. Thus, hospitalization in the absence of an adverse event is not regarded as an AE, and is not subject to expedited reporting. The following are examples of hospitalization that are not considered to be AEs:
  - Protocol-specified admission (e.g. for procedure required by study protocol)
  - Admission for treatment of target disease of the study, or for pre-existing condition (unless it is a worsening or increase in frequency of hospital admissions as judged by the clinical investigator)

- Diagnostic admission (e.g. for a work-up of an existing condition such as persistent pretreatment lab abnormality)
- Administrative admission (e.g. for annual physical)
- Social admission (e.g. placement for lack of place to sleep)
- Elective admission (e.g. for elective surgery)

Important medical events that may not result in death, be life-threatening, or require hospitalization may be considered a serious adverse drug experience when, based upon appropriate medical judgment, they may jeopardize the patient or subject and may require medical or surgical intervention to prevent one of the outcomes listed above.

9.3.4 Serious Adverse Event Reporting Requirements

SAEs (as defined above) must be reported from the time study product is dispensed in Stage 1B up to and including the individual participant's final study contact.

Any adverse event that is determined to be serious (whether expected or unexpected) regardless of relationship to the study agent(s) must be immediately reported to Dr. McGowan, CONRAD, and the NICHD Medical Officer (21 CFR 312.64). An SAE Form must be completed and sent to Dr. McGowan, CONRAD, and the NICHD Medical Officer within 3 business days (by 5 PM Eastern Time (ET)) after site awareness that the event has occurred at a reportable level. NICHD MO will review and discuss the SAE report with CONRAD if there are any concerns. An SAE Form must be completed and sent to the IRB per local IRB regulations.

CONRAD will notify the FDA of any unexpected serious adverse events associated with the use of the drug as soon as possible, but no later than 7 calendar days after initial receipt of the information from the investigator.

For unexpected serious adverse events associated with the use of the drug, CONRAD will submit the safety reports provided by the sites to the IND no later than 15 calendar days after the initial receipt of the information and send copies of the submission to the NICHD MO and Dr. McGowan. Dr. McGowan or his designee at the coordinating center (University of Pittsburgh) will distribute the IND safety reports to all the study sites.

Expected AEs are AEs that have been previously observed with use of the study agent(s) and are listed in the package insert or Investigator's Brochure. Expectedness is not based on what might be anticipated from the pharmacological properties of the study agent.

Unexpected AEs are AEs for which the nature or severity (intensity) is not consistent with the applicable agent information (Investigator's Brochure, package insert, or summary of agent characteristics).

Study Agents for SAE Reporting

The study agents that must be considered in determining relationships of AEs requiring expedited reporting to CONRAD and the NICHD MO are: tenofovir 1% gel, HEC placebo gel, and the gel applicators.

9.3.5 Social Harms Reporting

Although study sites make every effort to protect participant privacy and confidentiality, it is possible that participants' involvement in the study could become known to others, and that social harms may result (i.e., because participants could become known as HIV-infected or at "high risk" for HIV infection). For example, participants could be treated unfairly or discriminated against, or could have problems being accepted by their families and/or communities. Social harms that are judged by the site investigator to be serious or unexpected will be reported to responsible site IRB at least annually, or according to their individual requirements. In the event that a participant reports social harm, every effort will be made by study staff to provide appropriate care and counseling to the participant, and/or referral to appropriate resources for the safety of the participant as needed.

10 CLINICAL MANAGEMENT

Guidelines for clinical management and product hold/discontinuation are outlined in this section.

In general, the site investigator has the discretion to hold study product at any time if she/he feels that continued product use would be harmful to the participant or interfere with treatment deemed clinically necessary. Unless otherwise specified below, the investigator should immediately consult Dr. McGowan for further guidance on resuming study product, continuing the hold temporarily, or progressing to permanent discontinuation.

The site investigator or designee will document all product holds and discontinuations on applicable case report forms.

10.1. Grading System

The primary grading system is located in the Rectal Grading Table for Use in Microbicide Studies, which is labeled as Addendum 3 in the Division of AIDS Table for Grading Severity of Adult and Pediatric Adverse Events (DAIDS AE Grading Table), Version 1.0, December 2004, which can be found on the RCC website: http://rsc.tech-res.com/safetyandpharmacovigilance/.

10.2. Dose Modification

No dose modifications will be undertaken in this study.

10.3. Discontinuation of Study Product in the Presence of Toxicity

Grade 1 or 2

In general, participants who develop a Grade 1 or 2 AE regardless of relatedness to study product that is not specifically addressed below may continue use of study products (i.e., placebo gel or tenofovir 1% gel) per protocol.

Grade 3

Participants who develop a Grade 3 AE or toxicity that is not specifically addressed below and is judged to be related to study product should have study product permanently discontinued.

Grade 4

Participants who develop a Grade 4 AE or toxicity that is not specifically addressed below (regardless of relationship to study product) should have the study product permanently discontinued.

10.4. General Criteria for Discontinuation of Study Product

Study participants will be permanently discontinued from product use by the site investigator or designee in the event of HIV seroconversion.

10.5. Management of Specific Adverse Events

Specific product hold requirements are specified here in the context of clinical management of adverse events.

10.5.1 Hemorrhage Following Rectal Mucosal Biopsy

If bleeding continues after the anoscopy procedure that is uncontrolled (occurring between bowel movements) and results in the passage of blood clots per rectum, the participant will be referred for assessment in the emergency department of the nearest hospital.

10.5.2 Infection Following Rectal Mucosal Biopsy

The rate of local or systemic infection following anorectal biopsy is very low (< 1 per 1,500 - R Cranston personal communication). Any participant presenting with local or systemic features compatible with infection (fever, localized anorectal pain, anal discharge) will be referred to the emergency department of the nearest hospital.

10.5.3 Perforation of Rectum Following Rectal Mucosal Biopsy

The rate of perforation of a hollow viscus following endoscopic biopsy is less than 0.88:1,000.[93] However, anoscopic rectal sampling is even less likely to perforate a hollow

viscus due to sampling being below the reflection of the pelvic peritoneum and the absence of insufflation for the procedure. Any participant presenting with local or systemic clinical features suggestive of this condition (abdominal pain, swelling, fever) will be referred to the emergency department of the nearest hospital.

10.6. Criteria for Early Termination of Study Participation

Participants may voluntarily withdraw from the study for any reason at any time. The site investigator also may withdraw participants from the study to protect their safety and/or if they are unwilling or unable to comply with required study procedures, after consultation with the Principal Investigators. Participants also may be withdrawn if the study sponsors, government or regulatory authorities (including the Office of Human Research Protections), or site IRBs terminate the study prior to its planned end date. Every reasonable effort is made to complete a final evaluation of participants who withdraw or are withdrawn from the study prior to completing follow-up. Study staff members will record the reason(s) for all withdrawals in participants' study records. In the event that participants who voluntarily withdraw from the study wish to re-join the study, they may resume product use (if applicable) and follow-up through their originally scheduled study exit date.

11 STATISTICAL CONSIDERATIONS

11.1. Overview and Summary of Design

This study is a two-stage longitudinal safety, acceptability, and adherence study, including clinical and behavioral evaluations. After completing a screening evaluation, 280 eligible participants, including 40 sex workers, will be enrolled into Stage 1A of the study during which they will undergo a baseline medical evaluation for both history and presence of STIs and anorectal health pathologies or injuries, as well as a detailed Web-based baseline behavioral assessment. The first 140 eligible participants, including 20 sex workers, reporting at least one occasion of unprotected RAI in the previous 3 months will be invited to enroll into Stage 1B. In Stage 1B participants will apply the universal placebo gel (HEC) rectally using an applicator designed for rectal use prior to each episode of RAI over a 3-month period, reporting each use via a phone reporting system; they will complete a Web-based questionnaire and take part in a video teleconference at the end of the 3 months. The subset of sex workers who took part in Stages 1A and 1B will terminate participation at the end of 1B. The first 24 eligible participants completing Stage 1B will be invited to enroll in Stage 2. Eligible participants will be randomized to receive either tenofovir 1% gel or HEC placebo gel as part of Stage 2, the Phase 1 safety study. Following a baseline visit, participants will return to the clinic, where a single dose of the study gel will be administered rectally using an applicator designed for vaginal use. Within approximately 30 minutes, rectal swab, and rectal biopsy specimens will be obtained via anoscopy. After a one-week recovery period participants will return to the clinic for assessment. If no significant adverse events (AEs) are reported they will begin to self-administer once-daily

outpatient doses of the study gel for 7 days, after which they will return to the clinic for evaluation and specimen collection.

Figure 1. Study Schema

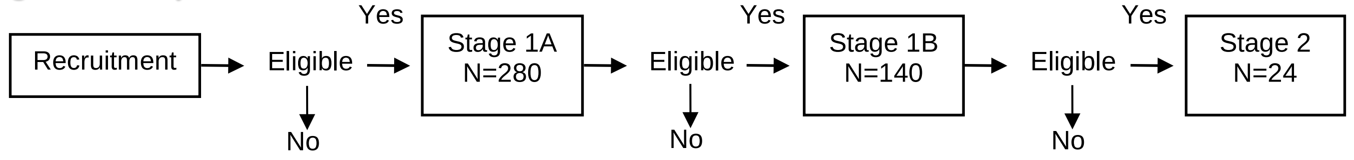

11.2. Study Endpoints

11.2.1 Clinical Endpoints

Stage 1AB

- Primary endpoint: Presence of STIs and anal and rectal pathologies as detected by standard anoscopy
- Secondary endpoint: Percent of agreement between reports of anal and rectal pathologies by 2 assessment methods (standard anoscopy versus high-resolution anoscopy)

Stage 2

- Primary endpoint: Grade 2 or higher AEs, as defined by the Division of AIDS Table for Grading the Severity of Adult and Pediatric Adverse Events, Version 1.0, Dec 2004 and/or Addenda 3 (Rectal Grading Tables for Use in Microbicide Studies)
- Exploratory endpoints: Changes in the following parameters:
    - Intestinal histopathology
    - Intestinal mucosal mononuclear cell phenotype
    - Intestinal mucosal cytokine messenger RNA (mRNA)
    - Cytokine profile in rectal secretions

11.2.2 Behavioral Outcomes

Stage 1AB and Stage 2

- Primary outcomes:
    - Identification of factors related to acceptability and adherence
    - Proportion of participants who report via the acceptability questionnaire that they would be very likely to use a similar candidate microbicide gel during receptive anal intercourse (RAI)
    - Proportion of RAI episodes in which the gel was used by participants
    - Comparison between self-reports of gel use and applicator counts
- Secondary outcomes:

- Identification of factors related to sexual behaviors (e.g., douching, lubricant use, recreational drug use, condom use, partner selection)
- Prevalence of risky sexual practices, douching, lubricant use, recreational drug use, and condom use

- Tertiary outcomes:
  - Proportion of individuals identifying as sex workers screened to individuals enrolled in the sex worker cohort
  - Proportion of visits completed by participants enrolled in the sex worker cohort
  - Likelihood of using microbicide gels, PrEP, or a rapid HIV Home Test with clients and non-commercial partners for individuals identifying as sex workers

11.3. Accrual, Sample Size, Randomization, and Blinding

The study will recruit from the three study sites a total of 280 sexually active, ethnically diverse, HIV uninfected men, age 18-30, who report engaging in RAI using condoms inconsistently or not at all. In Stage 2, approximately 24 participants will progress to randomization to one of two study arms at a 1:1 ratio. Based on prior studies with similar eligibility requirements, each site is expected to enroll 8 participants per month. Therefore accrual is anticipated to take approximately 6 months. The target for retention will be 95% of enrolled participants over the study period. To preserve the study power in the case of discontinuation/non-adherence, additional participants may enroll, at discretion of the protocol team, to replace participants who are discontinued or non-adherent to study product or scheduled study visits. Therefore the total sample size may be slightly exceeding 280 at the end of the study.

Each study site will be provided with a series of numbered, sealed envelopes containing the randomization assignment for each participant. The envelopes will be assigned sequentially by site staff. Each participant will be assigned a product code number. Using a blinded list of product codes and assigned products, the pharmacist at each site will supply the study product. Multiple codes will be utilized to conceal and protect the randomization assignments in this study.

Throughout the period of study implementation, neither study staff nor participants will be informed of the participants' random assignments. Study staff and participants will be unblinded after all study visits are completed. As described in Section 10, if an investigator is concerned that a participant might be put at an undue risk by continuing product use, the investigator may discontinue the product use by this participant and document the discontinuation. In emergency situations, if a participant experiences an SAE, that in the opinion of the investigator requires unblinding to protect participant safety, the investigator will notify the Principal Investigators to consider and rule upon the request when possible.

For the proposed study sample size of each stage, the statistical properties are summarized below.

Stage 1A

In preliminary studies with MSM in Adolescent Medicine Trials Network (ATN) for HIV/AIDS Interventions trials[94-96], the HIV prevalence has ranged from 5% to 20%. Table 21 shows the required sample sizes in order to ensure a certain margin of error (length between the point estimator, percentage, and either of the two limits, i.e., half of the length of the confidence interval). The table below demonstrates that the proposed sample of 280 subjects will be sufficient to give a precise estimate of the HIV and other STIs prevalence estimates. As shown in Table 22 below, the sample size will not be large enough to detect stable and precise HIV & STI incidence rates.

Table 21: Prevalence Power Calculations

Estimated HIV prevalence (p)	Margin of error (L)	Sample size
.05	0.03	203
.05	0.04	114
.05	0.05	73
.10	0.03	384
.10	0.04	216
.10	0.05	138
.15	0.03	544
.15	0.04	306
.15	0.05	196
.20	0.03	683
.20	0.04	384
.20	0.05	246

Table 22: Power calculations for HIV/STI Incidence

Estimated Incidence	Margin of Error (L)	Sample size
0.03	0.01	1,118
0.03	0.015	497
0.04	0.01	1,475
0.04	0.015	656
0.04	0.02	369
0.05	0.01	1,825
0.05	0.015	811
0.05	0.02	456

Stage 1B

Given the limited information available on adherence to rectal gel use, the power analysis was based on parameter estimates (number of partners and number of RAI occasions in the past two months, and likelihood of rectal microbicide use in the future) from the NICHD funded microbicide acceptability trial conducted in Boston, MA with MSM (# R01-HD46060). From this dataset, 103 participants were randomly assigned to three potential adherence trajectories: no change (stabilizing slope), low adherence (decreasing slope), or high adherence (increasing slope). The assumption was made that at baseline all subjects would have an equal probability of gel use or not use. Based on previous studies on applicator use[97], it was conservatively estimated that about 5% to 10% of them would not change their applicator use rate over the 12 week study period, about 30% to 35% of them would consistently decrease their applicator use rate by 5% per week, and the remaining 60% to 65% of subjects would increase their applicator use rate by 5% per week in the same time period. With the assumption that the study

Table 23: Power to detect various applicator use-rate increases over time with sample size 140 and Type I error rate controlled at .05 level.

Percentage mixtures of the 3-applicator use trajectories (increase, decrease, constant)	Use-rate increase per week	Power
(.60, .35, .05)	1.0%	.83
(.60, .30, .10)	1.2%	.94
(.65, .30, .05)	1.4%	.98
(.65, .25, .10)	1.6%	.99

population displays various percentage mixtures of the three applicator-use trajectories (stabilized, decreasing, and increasing), it was estimated that the applicator use rate would increase 1.0% to 1.6% per week with a standard deviation of .0345 to .0376. This power calculation was based conservatively to assume the largest estimated standard deviation (i.e., .0376). As shown in Table 23, with a sample size of 140 subjects, the proposed study the power to detect an increase of 1.0% per week or more with Type I error rate controlled at the.05 level.

<u>Stage 2</u>

Stage 2 will recruit participants from Stage 1B for a total of 24 sexually active, ethnic minority, HIV uninfected MSM from the three study sites. Within each study site, eight participants will be enrolled and randomized to tenofovir 1% or HEC placebo gel in a 1:1 ratio. Based on the accrual plan of Stage 1AB, each site is expected to enroll approximately 1-2 participants per month. Therefore accrual is anticipated to take approximately 6 months. The target for retention will be 95% of enrolled participants over the study period. To preserve the study power in the case of discontinuation/non-adherence, additional participants may enroll, at discretion of the protocol team, to replace participants who are discontinued or non-adherent to study product or scheduled study visits. Therefore the total sample size may be slightly exceeding 24 at the end of the study.

For the proposed study sample size, the statistical properties of this study in assessing the safety of study products are summarized below. With 12 participants in each study arm, the probability of observing zero safety events, at least one safety event, and two or more safety events are listed in the following table assuming various true event rates. For instance, if the true rate of a safety endpoint is 5%, the probability of observing that endpoint in at least one participant out of 12 participants is 46%. A higher true event rate will result in a larger probability to observe at least one event.

Table 24: Power Consideration for Stage 2

Event Rate	*Pr(0 event/n=12)*	*Pr(> 1 event/n=12)*	*Pr(> 2 events/n=12)*
1%	*0.89*	*0.11*	*0.01*
5%	*0.54*	*0.46*	*0.12*
10%	*0.28*	*0.72*	*0.34*
15%	*0.14*	*0.86*	*0.56*
25%	*0.03*	*0.97*	*0.84*

The statistical properties of this study may also be characterized by the width of the confidence intervals (CI) around observed event rate. The following table presents the exact 95% confidence intervals (Clopper-Pearson method) of the estimated rate when zero, one, or two endpoints are observed among 12 participants:

Table 25: Confidence Intervals

Number of Endpoints	Lower Bound of CI	Upper Bound of CI
0	0.0%	26.5%
1	0.2%	38.5%
2	2.1%	48.4%

11.4. Data Analysis

Descriptive statistics and graphics will be used to summarize participant characteristics. For categorical variables, the numbers and the proportions will be tabulated; for continuous variables, the mean, median, standard deviation, and quartiles will be reported. To assess the change of an endpoint from baseline to post-visit levels, McNemar's test (for categorical variables) or paired *t*-test (for continuous variables) will be used. To assess the difference of certain endpoints between groups, Chi-square tests will be used for categorical variables with exact *P*-values if the expected cell count in some stratum is small; t-test or linear regression will be used for continuous variables; nonparametric methods such as Wilcoxon rank-sum test may be used if sample size is small and data are non-normal. Generalized linear models will be used to regress continuous or categorical response variables, with or without adjusting for important baseline predictors. The longitudinal data will be analyzed using generalized estimation equations (GEE) with robust variance estimates.

11.4.1 Primary Analysis of prevalence of anal and rectal pathologies and STIs that may facilitate HIV infection

The analytic goal is to determine whether or not naked eye exam is equivalent to high-resolution anoscopy to assess anorectal clinical signs. Outcome measures will be Yes or No for a variety of signs and an overall Yes / No for any of these signs. The percent agreement and Cohen's Kappa will be calculated for each. Kappa values above 0.7 are indicative of strong agreement between the two methods. Percent agreement values of 0.8 or higher will be used as well.

In order to assess whether the two methods are statistically different, the 95% confidence interval of the difference between the proportion having the sign based on the anoscopy from the proportion having the sign based on the exam will be calculated. If the upper and lower bounds are within an acceptable threshold equivalence will be concluded. With a sample size of 240 (non sex-workers) and assuming an anoscopy rate of 20% the study is sufficiently powered to accept equivalence with a + or – 10% difference.

11.4.2 Primary and Secondary Analysis prevalence of behavioral practices associated with anal intercourse that may detract from microbicide effectiveness

The primary indicators of sexual risk behavior will be frequency of unprotected anal intercourse (UAI, receptive and/or insertive) in the prior three months. The UAI count variable will likely have a skewed distribution (due to atypically high frequencies of sexual activity among a relatively small proportion of the sample) and thus undergo an appropriate transformation prior to analyses. The receptive and/or insertive variable will be a dichotomous variable indicating whether the participant reported any unprotected anal intercourse in the prior three months. The generalized linear models (GLM) will be constructed using the normal distribution and an identity link for the continuous outcome (frequency of UAI), and the binomial distribution and a logit link for the dichotomous sexual risk variable (no UAI vs. some UAI).

In the multivariate analyses (GLM models with normal distribution for the continuous risk variable and binomial distribution for the dichotomous risk variable), a final model will be fitted selecting from the candidate variables identified in exploratory analyses using backward stepwise elimination of non-significant variables. This multivariate model will reveal independent effects of predictors on the outcome. Secondary analyses will seek to simplify the multiple regression model by constructing a composite index for each of the major independent domains based on the independent variables which enter the final regression model. If the proportion of explained variance remains close to that of the detailed regression model (within 5 percentage points), the simplified regression model will be reported for ease of interpretation of the results.

Investigators may propose additional analyses for subsequent publications (e.g., the association between demographic variables and study burden; concordance between phone reports of applicator-use and product counts; and, the prevalence of sexual behaviors and likelihood of rectal microbicide use). Research questions, analyses, results, and their interpretation will be developed by members of all research teams involved.

11.4.3 Primary and Secondary Analysis on acceptability of and use-adherence to study gel and applicators

<u>Qualitative Data</u>

All teleconferences will be audio (not video) recorded. The recording of the pre-trial teleconferences will only be used to make sure that the voice of the participant is clearly registered and that there are no technical problems. The recording of the end-trial teleconferences will be transcribed verbatim. As soon as an interview has been completed it will be sent for transcription, and shortly after the transcript is received, it will be checked for accuracy against audio recordings to make sure there are no missing or incorrect data. Once the transcripts are verified, they will be entered in NVivo (QSR International Pty Ltd, Cambridge MA), a software program designed for qualitative data analysis, and the audio files will be destroyed.

A list of major themes with definitions, inclusion and exclusion criteria, and examples will be developed based on interview guides. These themes will constitute the first level codes that will be used to analyze the transcripts. Using these codes, coders will independently code six or seven transcripts. The coded material will then be compared to identify concurrence or lack of it in the coding. The codebook will be further specified and new independent coding will be conducted until an 80% intercoder convergence is reached. From this point onwards, each transcript will be coded by only one team member.

Once all the interviews have been coded, code reports will be generated for each primary code. All of these codes will be integrated into a single database that will make it possible to take advantage of the built-in possibilities of NVivo to determine overlap between different codes and the creation of analytic matrices. Secondary codes

emerging from the data will be developed by the research team, once again with definitions, inclusion and exclusion criteria, and examples. This iterative work will lead to the identification of topics related to acceptability and adherence of the placebo gel that may constitute the focus of a scientific report, either based exclusively on the qualitative data or combined with quantitative results. In the latter case, the qualitative data will contextualize and provide nuance to the quantitative data collected in this study. This follows the model proposed by Morrow and Ruiz[9] and Morrow et al. for conducting microbicide acceptability studies.[20] Furthermore, from the data collected in Stage 1B new items may be developed to be included in the quantitative assessment of Stage 2.

<u>Quantitative Data</u>
As a first step, descriptive statistics will be used to describe the overall acceptability of the gel, the delivery device, and the method of application as reported in the Web-based assessments. ANOVAS and ANCOVAS will be used to explore associations of acceptability ratings with factors that are likely to influence them (e.g., presence or absence of leakage, partner reaction). Descriptive statistics will also be used to determine the proportion of sexual acts in which the gel was used, as tallied by the phone reporting system. Furthermore, the data collected though the phone reporting system will be analyzed with growth curve estimation (GCE) to model participants' product use adherence across the three-month period. Hierarchical Linear Modeling[98] will be used to conduct these analyses. Contrary to a repeated measures regression, growth curve estimation overcomes autocorrelation bias (i.e., within subject correlations due to repeated observations from any given individual) by allowing the variance to be divided into within-individual variation (level-1 model; i.e., change in adherence over time) and between-individual variation (level-2 model; i.e., person characteristics). This strategy will also adjust for any random effects introduced due to multi-site design. The level-1 GCE model assumes the within-variation for participant's adherence outcome (Y_{ti}) at each observed datapoint can be modeled by estimating his baseline adherence score (π_{0i}), the linear and nonlinear growth parameters (π_{pi}), and his associated within-individual random error term (e_{ti}), where t is the wave of each observation and i is the individual. As shown in Equation 1, this strategy assesses the association between the adherence curve and other time-varying covariates such number of partners across the three-month period.

Equation 1: $\text{Adherence}_{ti} = \pi_{0i} + \pi_{1i}\,(\text{Time})_{ti} + \pi_{2i}\,(\text{Number of Partners})_{ti} + e_{ti}$

The level-2 GCE model tests whether the growth slopes vary across different person characteristics such as race and income. Cultural and epidemiologic differences may lead to variation in respondents' adherence trajectories. As shown in Equation 2, interethnic variations will be tested for in the level-1 model by including race as a moderator/interaction effect on the GCE predictors (baseline, changes over time, and number of partners).

Equation 2: $\pi_{0i} = B_{00} + B_{01}(\text{Black}) + B_{02}(\text{Latino}) + r_{0i}$
$\pi_{1i} = B_{10} + B_{11}(\text{Black}) + B_{12}(\text{Latino}) + r_{1i}$
$\pi_{2i} = B_{20} + B_{21}(\text{Black}) + B_{22}(\text{Latino}) + r_{2i}$

Consequently, the level-2 model can be modeled with a grand mean for White youth (B_{x0}), an additive effect for Black and Latino respondents (B_{x1} and B_{x2}, respectively), and a between-individual random error term (r_{pi}).

11.4.4 Primary and Secondary Analysis on Safety of Tenofovir 1% gel and HEC Placebo gel

The frequency of > Grade 2 adverse events as defined by the DAIDS Genital Toxicity Table (released in 2007) will be used as the primary measure of safety of tenofovir 1% gel. Analyses of safety endpoints will be based on descriptive summaries of frequency and severity of safety endpoints among men in both study arms. Endpoints will be considered singly and jointly. For each type of endpoint, proportions of participants affected will be calculated with accompanying exact binomial 95% confidence limits. Event rates will be compared between arms using a Fisher's Exact test with a one-sided alternative.

11.4.5 Secondary Analysis on the Mucosal Damage by Tenofovir 1% gel

As participants in Stage 2 will be randomized to the two treatment groups, systematic baseline differences are not expected; however, due to the relatively small study size, it is quite likely that many parameters will be quite different between treatment groups at baseline. All analyses will therefore be conducted both with and without controlling for baseline measurements. For continuous measures, it is well-known that controlling for baseline differences in regression models is more powerful than analyzing changes from baseline.[99]

The basic model for analyzing continuous measures adjusting for baseline differences will be the analysis of covariance (ANCOVA) model regressing the continuous parameter at either post-single dose (Visit 7) or post-7 daily doses (Visit 9) on the continuous baseline value and the categorical treatment group membership (with placebo as the reference group). The estimated regression coefficients for the treatment group will then be directly interpretable as the difference between tenofovir 1% gel vs. placebo, post-single dose (Visit 7) or post-7 daily doses (Visit 9) after adjusting for baseline differences. This model without the baseline values reduces to the simple one-way ANOVA model with treatment group as the predictor. For analyzing binary or ordinal measures, the logistic regression version of these models will be used. That is, the outcome post-single dose (Visit 7) or post-7 daily doses (Visit 9) will be regressed on group membership and on baseline value (when adjusting for baseline differences).

The multiple comparisons problem will be addressed both informally and formally. Informally, particular group differences significant at the 0.05 level or smaller will be flagged as at least suggestive and those with extremely strong p-values (e.g. 0.001 or less) will be carefully examined. In addition, differences seen consistently for related parameters will carry more weight. Formally, corrections to the p-values will be applied

using step-down methods[100] implemented in the Multitest procedure in SAS v 9.1 (SAS Institute, Cary, NC), which are more powerful than the Bonferroni method.

Longitudinal (three or four time points) and/or multivariate (simultaneous examination of multiple measures in a set) modeling techniques will also be investigated. The longitudinal analyses will most likely not be additionally informative, as there are only three time points for most of the parameters. Multivariate techniques are expected to result in potentially substantial power increases. Previous studies with phenotype and cytokine panels have shown strong correlations between measures as well as good stability over time (i.e. high intrasubject correlations) in steady state for many of the measures. In this setting, for example, factors derived using principal components analysis would likely have stronger association with group membership than any one particular measure in the set. An additional strength of the longitudinal and multivariate approaches is that they provide an alternative solution to the multiple testing problems that arise with examining each time point and each parameter individually.

11.4.6 Analysis of Specific Mucosal Parameters

- **Histology:** Slides will be reviewed and scored by a qualified pathologist using a qualitative scoring system (Appendix III) used in previous studies.[14] Examination of two point (normal versus abnormal) or three point (normal, slightly abnormal, abnormal) scales will be undertaken.
- **Flow cytometry:** Many of the flow cytometry measurements from the HPTN 056 study are quite stable across time. Parameters with high intrasubject correlations (RFI measures are log-transformed) include %CD3, %CD4, %CD8, %CD31, CD38RFI on CD4+, CD38RFI on CD8+, %CD38||CD4, and %CD38||CD8 which have intrasubject correlations of between 0.7 and 0.9.[14] It is expected that clinically important differences in these parameters between treatment groups will be found to be statistically significant by these analyses.
- **Mucosal Cytokine levels:** The cytokine data, in HPTN 056 and earlier work, for RANTES, IFN-γ, and IL-10 all showed strong stability (intrasubject correlations between 0.7 or 0.8; analysis is on log-transformed scale for all three of these).[14] Again, this suggests that any clinically meaningful differences between treatment groups are likely to result in small p-values.

11.4.7 Exploratory Analyses of Mucosal Endpoints and Correlation Between Histological Abnormality and Changes in Mucosal Biomarkers

Analyses will be performed to evaluate the correlation between mucosal biomarkers and histological abnormality across arms and three time points. At each time point, various cytokines, cell phenotypes or calprotectins will be compared between groups with different levels of histological abnormality, defined by histopathology. Longitudinal data modeling (GEE method) combining three time points will be employed to evaluate the collected association of biomarkers with mucosal damage over the study period.

11.4.8 Tertiary Analysis of Feasibility of Recruitment and Retention of Sex Workers and their Likelihood of Use of Non-Condom Based HIV Prevention Strategies

The primary purposes of this pilot study are to demonstrate the feasibility of methods proposed for a subsequent large-scale trial, to stabilize procedures so they are replicable, and to determine important parameters with sufficient accuracy to allow reliable estimates of sample size and for the subsequent study. "Mission-critical" parameters include proportions for dichotomous variables (such as the recruitment rate (i.e., proportion of individuals identifying as sex workers screened to individuals enrolled in the sex worker cohort) and the retention rate (i.e., proportion of visits completed by participants enrolled in the sex worker cohort)); means and standard deviations for continuous endpoints (such as the likelihood of using microbicide gels, PrEP, or a rapid HIV Home Test with clients and non-commercial partners for individuals identifying as sex worker). Thus, statistical planning of this pilot study aims to ensure that the mission-critical parameters for the large trial are estimated as informatively as possible. For the dichotomous variables, exact binomial methods will be employed to estimate outcome proportions together with one-sided 90% confidence intervals for subsequent trial planning. The lower confidence limit for the recruitment rate is given by the value P_L that satisfies the equation $\Sigma_i \; {}_nC_i \; P_L^{\,i}(1–P_L)^{n–i} = 0.10$ where ${}_nC_i$ is the binomial coefficient n-choose-i for n sex worker screened, and where the sum extends over indices i greater than or equal to the observed number of participants enrolled in the study whereas for the lower limit for the retention rate, n represents the sample size and the sum extends over indices i greater than or equal to the observed number of participants to the primary endpoint. For the continuous measure such as the likelihood of product use, means and standard deviations will be estimated. For approximately normally distributed variables, an upper one-sided 90% confidence limit for the standard deviation, σ_U will be constructed from $\sigma_U = s\cdot(\chi^2_{\nu,.10})^{1/2}$ where s is the sample standard deviation and $\chi^2_{\nu,.10}$ is the upper 10th percentile of the chi-squared distribution with ν degrees of freedom. For approximately log-normally distributed variables, a logarithmic transformation will be applied to achieve approximate symmetry and normality.

12 DATA HANDLING AND RECORDKEEPING

12.1. Data Management Responsibilities

Study case report forms will be developed in conjunction with the protocol team.

12.2. Source Documents and Access to Source Data/Documents

All study sites will maintain source data/documents in accordance with NIH requirements. Each investigator will maintain, and store securely, complete, accurate and current study records throughout the study. In accordance with US regulations, for each of the investigational products tested, the investigator will retain all study records for at least two years following the date of marketing approval for the study product for the indication in which it was studied. If no marketing application is to be filed or if the application is not approved, the records must be retained until two years after the investigation is discontinued and the US FDA is notified.

Study records must be maintained on site for the entire period of study implementation. Thereafter, instructions for record storage will be provided by CONRAD. No study records may be moved to an off-site location or destroyed prior to receiving approval from CONRAD.

12.3. Quality Control and Quality Assurance

All study sites will conduct quality control and quality assurance procedures in accordance with NIH/NICHD/NIMH requirements.

12.4. Study Activation

Following IRB review and approval, all study sites will request permission to begin enrollment from the Principal Investigators.

12.5. Study Coordination

CONRAD holds the IND applications for this study. Assignment of all sponsor responsibilities for this study will be specified in a Clinical Trials Agreement (CTA) executed by CONRAD and the Principal Investigators.

Study implementation will be directed by this protocol, which may not be amended without prior written approval from the Principal Investigators and the NICHD Medical Officer.

Close coordination between protocol team members is necessary to track study progress, respond to queries about proper study implementation, and address other issues in a timely manner. The Principal Investigators will address issues related to participant eligibility and AE management and reporting as needed to assure consistent case management, documentation, and information-sharing across sites. Rates of accrual, adherence, follow-up, and AE incidence will be monitored closely by the team.

13 COMPLIANCE WITH APPLICABLE LAWS

All clinical research supported by NIH must comply with U.S., state, and local regulations. Whenever the regulations differ between authorities, the more restrictive regulation will apply.

14 CLINICAL SITE MONITORING

Site visits by NIH personnel, CONRAD, or their contractor representatives may be conducted periodically to monitor study conduct, documentation, and GCP compliance. Visit frequency will be determined by the NIH branch program officer and CONRAD, and specific visit dates will be determined by mutual agreement of site investigator and the NIH branch program officer. The NIH will conduct direct oversight of GCP compliance documentation during the Program Officer site visits for Stage 1A and Stage 1B; however, regular contractor monitoring visits are not anticipated. CONRAD will conduct direct oversight of GCP compliance documentation during monitoring visits for Stage 2. NIH and CONRAD reserve the discretion to revisit questions of periodic monitoring and AE reporting and revise these arrangements if appropriate should there be any indication of unanticipated frequency or severity of AE's, or unanticipated operational difficulties. The site investigator will facilitate such site visits and will ensure that visiting NIH and CONRAD staff members (or their representatives) are provided full access to study personnel, records, and facilities.

Study monitors will visit the site to do the following:

- Review informed consent forms, procedures, and documentation
- Assess compliance with the study protocol and applicable regulatory requirements (US and non-US), including the International Conference on Harmonisation E6: Good Clinical Practices (GCP), Section 6 and the US Code of Federal Regulations (CFR) Title 45 Part 46 and Title 21 Parts 50**,** 56, and 312.
- Perform source document verification to ensure the accuracy and completeness of study data
- Verify proper collection and storage of biological specimens
- Verify proper storage, dispensing, and accountability of investigational study products
- Assess implementation and documentation of internal site quality management procedures
- Assess site staff training needs

Site investigators will allow study monitors to inspect study facilities and documentation (e.g., informed consent forms, clinic and laboratory records, other source documents, case

report forms), as well as observe the performance of study procedures. Investigators also will allow inspection of all study-related documentation by authorized representatives of CONRAD, NIH, and US regulatory authorities. A site visit log will be maintained at the study site to document all visits.

15 HUMAN SUBJECTS PROTECTIONS

Before study initiation, the Principal Investigators are responsible for submitting written documentation to NICHD that all study staff responsible for the design or conduct of the research have received appropriate training in the protection of human subjects. The investigators will make efforts to minimize risks to participants. Volunteers and study staff members will take part in a thorough informed consent process. Before beginning the study, the investigators will have obtained IRB approval and the protocol will have been submitted to the FDA. The investigators will permit audits by the NIH, CONRAD, FDA, OHRP, or any of their appointed agents.

15.1. Institutional Review Boards

Each participating institution is responsible for assuring that this protocol and the associated site-specific informed consent documents and study-related documents (such as participation education and recruitment materials) are reviewed by an Institutional Review Board (IRB) responsible for oversight of research conducted at the study sites. Any amendments to the protocol must be approved by the responsible IRB and the Principal Investigators prior to implementation.

Subsequent to the initial review and approval, the responsible IRBs must review the study at least annually. Each investigator will make safety and progress reports to the IRBs at least annually and within three months after study termination or completion. These reports will include the total number of participants enrolled in the study, the number of participants who completed the study, all changes in the research activity, and all unanticipated problems involving risks to human subjects or others. In addition, the results of all safety reviews of the study will be provided to the IRBs.

15.2. Risk Benefit Statement

15.2.1 Risks

General

Phlebotomy may lead to discomfort, feelings of dizziness or faintness, and/or bruising, swelling and/or infection. There are no risks associated with the collection of oral rinse or urine specimens. Disclosure of STI status may cause sadness or depression in volunteers. Participation in clinical research includes the risks of loss of confidentiality and discomfort with the personal nature of questions.

Although study sites make every effort to protect participant privacy and confidentiality, it is possible that participants' involvement in the study could become known to others, and that social harms may result (i.e., because participants could become known as HIV-infected or at "high risk" for HIV infection). For example, participants could be treated unfairly or discriminated against, or could have problems being accepted by their families and/or communities.

Participants in sites requiring partner notification in response to diagnosed STIs or HIV infection could have problems in their relationships with their sexual partners. Participants also could have problems in their partner relationships associated with use or attempted use of study products. In addition, participants could misunderstand the current experimental status of the study gels (i.e., their unknown safety and unproven efficacy) and as a result increase their HIV risk behaviors while in the study.

Data on participant risk behaviors and the occurrence of other potential social harms are collected from all participants on a quarterly basis. The protocol team monitors trends in risk behaviors over time based on these data, as well as the occurrence of other potential social harms, and takes any required follow-up action.

Participants will also be counseled on the importance of using condoms throughout the duration of the study to minimize risk of unknown effects of the study gels on the rectum.

Buccal Swabs, Rectal Swabs, and Rectal Sponges
There is the risk of mild discomfort from both these procedures in addition to a slight risk of bleeding.

Anoscopy
Anoscopy is a commonly practiced medical procedure and the procedures done in this trial will not involve any unusual risks or discomforts. The risk associated with anoscopy is a small amount of bleeding.

Mucosal Biopsies
The biopsies are painless and heal quickly within 3 to 5 days. On extremely rare occasions, biopsies may lead to pain, bleeding or perforation of the gastrointestinal tract, or infection. Perforation occurs approximately less than once out of every 1,000 procedures. If this extremely rare complication occurs, antibiotics and surgery to repair the tear may be necessary.

Rectal Applicator
Use of the rectal applicator to deliver a microbicide into the rectal compartment may be associated with discomfort. The Fleet® comfort tip applicator is unlikely to be associated with even minor anorectal trauma (e.g., lacerations and bruising in the anorectal area).

Vaginal Applicator
Use of a vaginal applicator to deliver a vaginal microbicide into the rectal compartment may be associated with minor anorectal trauma including lacerations and bruising in the anorectal area.

HEC placebo gel
Unformulated hydroxyethylcellulose is known to be a non-irritating substance in humans (skin sensitization is unusual). There is currently no rectal safety data in humans regarding the use of HEC gel, but a recent rectal safety study in macaques did not reveal any safety issues. Human studies of vaginal application of HEC gel, however, have been conducted. Twice daily intravaginal administration of HEC gel over the course of two weeks resulted in mild genital irritation, including genital burning, soreness, and pelvic pain, in 2 out of 14 women (14.3%).[63, 72] Three out of the 14 women (12.4%) had colposcopic findings which included erythema, petechiae and peeling, although no findings with deep disruption were observed during follow-up. HEC gel did not appear to alter vaginal health or shift vaginal flora and no SAEs were reported.

Tenofovir 1% Gel
There is currently no rectal safety data regarding the use of the new tenofovir 1% gel formulation. Administration of the previous tenofovir gel formulation intravaginally at 0.3% and 1% concentrations in the HPTN 050 Phase 1 study resulted in minimal local irritation and little or no systemic adverse effects were identified.[89] Although 92% of participants reported at least 1 AE, 87% of those reported AEs were mild, and 70% of the AEs were limited to the genitourinary tract. Four severe AEs were reported, with only one, lower abdominal pain, thought to be product-related. The risks associated with tenofovir gel are believed to be less than those identified for systemic use. In the HPTN 050 Phase 1 study of tenofovir gel, serum PK analysis in a subset of participants demonstrated that there is no clinically significant systemic toxicity. Fourteen of 24 women with PK results had low, but detectable, serum tenofovir levels.

Given that Phase 1 data demonstrates measurable plasma concentrations of tenofovir in some participants, participants with hepatitis B infection might be at risk for development of tenofovir resistant hepatitis B. However, participants with known hepatitis B infection will not be eligible for enrollment. It is not known what effect tenofovir gel could have on the HIV virus or HIV disease progression in HIV infected participants or their partners. There is a theoretical risk that tenofovir absorbed systemically from rectal tenofovir gel could result in mutations of the HIV virus in participants who become infected with HIV during the study, or their partner, if the partner is infected with HIV. Limited resistance data from HPTN 050 show no new resistance mutations in plasma or cervicovaginal lavage specimens after 14 days of tenofovir gel use. No participant had high level tenofovir mutations (e.g., K65R).

In the male tolerance study of tenofovir 1% gel, there were few genital findings observed after product use and all findings were classified as mild, were small in size and required no treatment.[91] The most common symptoms included mild pain (burning, irritation, discomfort) and pruritus. All reported urogenital symptoms were felt to be mild.

Some of the possible side effects of the study gel are dryness, itching, burning, or pain in the rectal area.

15.2.2 Benefits

Participation in this study likely will have no direct benefit to volunteers other than access to screening for STIs and appropriate referral if STIs are diagnosed. Some volunteers may have the opportunity to access expedient treatment and decreased morbidity due to early diagnosis and treatment of abnormalities in serology, blood count, liver or kidney function tests. Lastly, the participants and others may benefit in the future from information learned from this study. Specifically, information learned in this study may lead to the development of safe and effective interventions to prevent HIV transmission. The participant may appreciate the opportunity to contribute to the body of knowledge in the field of microbicide research. However, there is no guarantee that volunteers will receive any of these benefits.

15.3. Informed Consent Process

Written informed consent will be obtained from each study participant prior to both screening and enrollment. In obtaining and documenting informed consent, the investigators and their designees will comply with applicable local and US regulatory requirements and will adhere to GCP and to the ethical principles that have their origin in the Declaration of Helsinki. Study staff must document the informed consent process. Participants will be provided with copies of the informed consent forms if they are willing to receive them.

Each study site is responsible for developing study informed consent forms for local use, based on the templates in the Appendices that describe the purpose of screening and of the study, the procedures to be followed, and the risks and benefits of participation, in accordance with all applicable regulations.

The informed consent process will cover all elements of informed consent required by research regulations. In addition, the process specifically will address the following topics of import to this study:

- The unknown safety and unproven efficacy of the study products
- The need to practice safer sex behaviors regardless of study treatment group
- The importance of participants in all study groups to the success of the study
- The importance of adherence to the study visit and procedures schedule

- The potential medical risks of study participation (and what to do if such risks are experienced)
- The potential social harms associated with study participation (and what to do if such harms are experienced)
- The real yet limited benefits of study participation
- The distinction between research and clinical care
- The right to withdraw from the study at any time

The informed consent process will include an assessment of each potential participant's understanding prior to enrollment and randomization of concepts identified by the protocol team as essential to the informed consent decision. Participants who are not able to demonstrate adequate understanding of key concepts after exhaustive educational efforts will not be enrolled in the study.

15.4. Participant Confidentiality

All study procedures will be conducted in private, and every effort will be made to protect participant privacy and confidentiality to the extent possible. Each study site will implement confidentiality protections that reflect the local study implementation plan and the input of study staff and community representatives to identify potential confidentiality issues and strategies to address them. In addition to local considerations, the protections described below will be implemented at all sites.

All study-related information will be stored securely at the study site. All participant information will be stored in locked file cabinets in areas with access limited to study staff. Data collection, process, and administrative forms, laboratory specimens, and other reports will be identified by a coded number only to maintain participant confidentiality. All local databases will be secured with password-protected access systems. Forms, lists, logbooks, appointment books, and any other listings that link participant ID numbers to other identifying information will be stored in a separate, locked file in an area with limited access. Participants' study information will not be released without their written permission, except as necessary for review, monitoring, and/or auditing by:

- NIH and/or its contractors, including study monitors
- Representatives of CONRAD
- The US FDA
- Office for Human Research Protections (OHRP)
- Site IRBs

15.5. Special Populations

This section outlines considerations made for the inclusion or exclusion of special populations in this study.

15.5.1 Children

The NIH has mandated that children be included in research trials when appropriate. This study meets “Justifications for Exclusion” criteria for younger children as set forth by the NIH. Specifically, “insufficient data are available in adults to judge potential risk in children” and “children should not be the initial group to be involved in research studies.” This study does not plan to enroll children under 18 years old.

15.6. Compensation

Pending IRB approval, participants will be compensated for their time and effort in this study, which may include reimbursement for travel to study visits, child care, and time away from work.

15.7. Communicable Disease Reporting

Study staff will comply with all applicable local requirements to report communicable diseases including HIV identified among study participants to local health authorities. Participants will be made aware of all reporting requirements during the study informed consent process.

15.8. Access to HIV-related Care

15.8.1 HIV Counseling and Testing

HIV test-related counseling will be provided to all potential study participants who consent to undergo HIV screening to determine their eligibility for this study, and to all enrolled participants at each follow-up HIV testing time point. Counseling will be provided in accordance with standard HIV counseling policies and methods at each site and additionally will emphasize the unknown efficacy of the study products in preventing HIV infection. In accordance with the policies of the US National Institutes of Health, participants must receive their HIV test results to take part in this study.

15.8.2 Care for Participants Identified as HIV-Infected

Participants will be provided with their HIV test results in the context of post-test counseling. Participants found to be HIV-infected will be referred to available sources of medical and psychosocial care and support, and local research studies for HIV-infected adults.

15.9. Study Discontinuation

This study may be discontinued at any time by the NIH, CONRAD, FDA, OHRP, or site IRBs.

16 PUBLICATION POLICY

The primary data sharing mechanism for dissemination of scientific information generated by this R01 grant will be through presentation of data at conferences and in scientific journals. In either situation the data will be unlinked from any identifying data. Once studies have been presented and/or published the team will consider presenting data in a public access format. On a day-to-day basis, data sharing between the groups will be facilitate though the use of a study specific website as has been done in previous program grants undertaken by members of this consortium

An agreement between CONRAD and the Principal Investigators will govern publication of the results of this study. Any presentation, abstract, or manuscript will be submitted by the investigator to CONRAD, for review prior to submission. All publications will acknowledge the financial support of the NICHD and NIMH.

17 LIST OF APPENDICES

APPENDIX I: **SCHEDULE OF STUDY VISITS AND EVALUATIONS**
APPENDIX II: **TOXICITY TABLES**
APPENDIX III: **HISTOPATHOLOGY SCORING SYSTEM**
APPENDIX IV: **SAMPLE INFORMED CONSENT FORM (STAGE 1A)**
APPENDIX V: **SAMPLE INFORMED CONSENT FORM (STAGE 1B)**
APPENDIX VI: **SAMPLE INFORMED CONSENT FORM (STAGE 2)**
APPENDIX VII: **SAMPLE INFORMED CONSENT FORM (SAMPLE STORAGE)**

18 REFERENCES

1. Sifakis F, Hylton JB, Flynn C, Solomon L, MacKellar DA, Valleroy LA, et al. Racial disparities in HIV incidence among young men who have sex with men: the Baltimore Young Men's Survey. J Acquir Immune Defic Syndr 2007 Nov 1;46(3):343-8.
2. Valleroy LA, MacKellar DA, Karon JM, Rosen DH, McFarland W, Shehan DA, et al. HIV prevalence and associated risks in young men who have sex with men. Young Men's Survey Study Group. JAMA 2000 Jul 12;284(2):198-204.
3. Koblin B, Chesney M, Coates T. Effects of a behavioural intervention to reduce acquisition of HIV infection among men who have sex with men: the EXPLORE randomised controlled study. Lancet 2004 Jul 3;364(9428):41-50.
4. MacQueen KM, Cates W, Jr. The multiple layers of prevention science research. Am J Prev Med 2005 Jun;28(5):491-5.
5. McGowan I. Microbicides: a new frontier in HIV prevention. Biologicals 2006 Dec;34(4):241-55.
6. Karim QA, Abdool Karim SS, Frohlich JA, Grobler AC, Baxter C, Mansoor LE, et al. Effectiveness and Safety of Tenofovir Gel, an Antiretroviral Microbicide, for the Prevention of HIV Infection in Women. Science 2010 Sep 3;329(5996):1168-74.
7. Elias C, Coggins C. Acceptability research on female-controlled barrier methods to prevent heterosexual transmission of HIV: Where have we been? Where are we going? J Womens Health Gend Based Med 2001 Mar;10(2):163-73.
8. Severy L, Newcomer S. Critical issues in contraceptive and STI acceptability research. Journal of Social Issues 2005;61(1):45-65.
9. Mantell JE, Myer L, Carballo-Dieguez A, Stein Z, Ramjee G, Morar NS, et al. Microbicide acceptability research: current approaches and future directions. Soc Sci Med 2005 Jan;60(2):319-30.
10. Morrow KM, Ruiz MS. Assessing microbicide acceptability: a comprehensive and integrated approach. AIDS Behav 2008 Mar;12(2):272-83.
11. Tolley EE, Severy LJ. Integrating behavioral and social science research into microbicide clinical trials: challenges and opportunities. Am J Public Health 2006 Jan;96(1):79-83.
12. WHO. Beyond acceptability: Users' perspectives on contraception. London: Reproductive Health Matters for WHO; 1997.
13. Rockefeller Foundation. The science of microbicides: Accelerating development. A report by the Science Working Group of the Microbicide Initiative. New York: Rockfeller Foundation; 2002.
14. McGowan I, Elliott J, Cortina G, Tanner K, Siboliban C, Adler A, et al. Characterization of baseline intestinal mucosal indices of injury and inflammation in men for use in rectal microbicide trials (HIV Prevention Trials Network-056). J Acquir Immune Defic Syndr 2007 Dec 1;46(4):417-25.
15. Dilley JW, Woods WJ, Sabatino J, Lihatsh T, Adler B, Casey S, et al. Changing sexual behavior among gay male repeat testers for HIV: a randomized, controlled trial of a single-session intervention. J Acquir Immune Defic Syndr 2002 Jun 1;30(2):177-86.
16. Tabet SR, Surawicz C, Horton S, Paradise M, Coletti AS, Gross M, et al. Safety and toxicity of nonoxynol-9 gel as a rectal microbicide. Sex Transm Infect 1999 Nov;26(10):564-71.

17. Turner CF, Ku L, Rogers SM, Lindberg LD, Pleck JH, Sonenstein FL. Adolescent sexual behavior, drug use, and violence: increased reporting with computer survey technology. Science 1998 May 8;280(5365):867-73.

18. Rosen RK, Morrow KM, Carballo-Dieguez A, Mantell JE, Hoffman S, Gai F, et al. Acceptability of tenofovir gel as a vaginal microbicide among women in a phase I trial: a mixed-methods study. J Womens Health (Larchmt) 2008 Apr;17(3):383-92

19. Bentley ME, Morrow KM, Fullem A, Chesney MA, Horton SD, Rosenberg Z, et al. Acceptability of a novel vaginal microbicide during a safety trial among low-risk women. Fam Plann Perspect 2000 Jul;32(4):184-8.

20. Morrow K, Rosen R, Richter L, Emans A, Forbes A, Day J, et al. The acceptability of an investigational vaginal microbicide, PRO 2000 Gel, among women in a phase I clinical trial. J Womens Health (Larchmt) 2003 Sep;12(7):655-66.

21. El-Sadr WM, Mayer KH, Maslankowski L, Hoesley C, Justman J, Gai F, et al. Safety and acceptability of cellulose sulfate as a vaginal microbicide in HIV-infected women. AIDS 2006 May 12;20(8):1109-16.

22. Koo HP, Woodsong C, Dalberth BT, Viswanathan M, Simons-Rudolph A. Context of acceptabilty of topical microbicides: Sexual relationships. J Social Issues 2005;61(1):67-93.

23. Tanner AE. Perceptions of acceptability and utility of microbicides in Ghana, West Africa: A qualitative, exploratory study. SAHARA J 2008 Apr;5(1):11-8.

24. Veldhuijzen N, Nyinawabega J, Umulisa M, Kankindi B, Geubbels E, Basinga P, et al. Preparing for microbicide trials in Rwanda: focus group discussions with Rwandan women and men. Cult Health Sex 2006 Sep;8(5):395-406.

25. Carballo-Dieguez A, Dolezal C. HIV risk behaviors and obstacles to condom use among Puerto Rican men in New York City who have sex with men. Am J Public Health 1996 Nov;86(11):1619-22.

26. Gross M, Celum CL, Tabet SR, Kelly CW, Coletti AS, Chesney MA. Acceptability of a bioadhesive nonoxynol-9 gel delivered by an applicator as a rectal microbicide. Sex Transm Dis 1999 Nov;26(10):572-8.

27. Exner TM, Correale J, Carballo-Dieguez A, Salomon L, Morrow KM, Dolezal C, et al. Women's anal sex practices: implications for formulation and promotion of a rectal microbicide. AIDS Educ Prev 2008 Apr;20(2):148-59.

28. Carballo-Dieguez A, Exner T, Dolezal C, Pickard R, Lin P, Mayer KH. Rectal microbicide acceptability: results of a volume escalation trial. Sex Transm Dis 2007 Apr;34(4):224-9.

29. Turner CF, Ku L, Rogers SM, Lindberg LD, Pleck JH, Sonenstein FL. Adolescent sexual behavior, drug use, and violence: increased reporting with computer survey technology. Science 1998 May 8;280(5365):867-73.

30. Carballo-Dieguez A, Dolezal C, Bauermeister J, Ventuneac A, O'Brien W, Mayer K. Preference for gel over suppository as delivery vehicle for a rectal microbicide: results of a randomised, crossover acceptability trial among men who have sex with men. Sexually Transmitted Infections 2008;84:483-7.

31. Carballo-Dieguez A, Exner T, Dolezal C, Pickard R, Lin P, Mayer KH. Rectal microbicide acceptability: results of a volume escalation trial. Sexually Transmitted Diseases 2007;34(4):224-9.

32. Poles MA, Elliott J, Taing P, Anton PA, Chen IS. A preponderance of CCR5(+) CXCR4(+) mononuclear cells enhances gastrointestinal mucosal susceptibility to human immunodeficiency virus type 1 infection. J Virol 2001;75(18):8390-9.

33. Leynaert B, Downs AM, de Vincenzi I. Heterosexual transmission of human immunodeficiency virus: variability of infectivity throughout the course of infection. European Study Group on Heterosexual Transmission of HIV. American Journal of Epidemiology 1998;148(1):88-96.

34. Vittinghoff E, Douglas J, Judson F, McKirnan D, MacQueen K, Buchbinder SP. Per-contact risk of human immunodeficiency virus transmission between male sexual partners. American Journal of Epidemiology 1999;150(3):306-11.

35. Gray RH, Wawer MJ, Brookmeyer R, et al. Probability of HIV-1 transmission per coital act in monogamous, heterosexual, HIV-1-discordant couples in Rakai, Uganda. Lancet 2001;357(9263):1149-53.

36. Padian NS, Shiboski SC, Glass SO, Vittinghoff E. Heterosexual transmission of human immunodeficiency virus (HIV) in northern California: results from a ten-year study. American Journal of Epidemiology 1997;146(4):350-7.

37. Geboes K, Riddell R, Ost A, Jensfelt B, Persson T, Lofberg R. A reproducible grading scale for histological assessment of inflammation in ulcerative colitis.[see comment]. Gut 2000;47(3):404-9.

38. Anton PA. A phase I safety and acceptability study of the UC-781 microbicide gel applied rectally in HIV seronegative adults: an interim safety report at 50% completion. In: Microbicides 2008; 2008; New Delhi, India; 2008.

39. Shacklett BL, Yang O, Hausner MA, et al. Optimization of methods to assess human mucosal T-cell responses to HIV infection. Journal of Immunological Methods 2003;279(1-2):17-31.

40. Anton PA, Elliott J, Poles MA, et al. Enhanced levels of functional HIV-1 co-receptors on human mucosal T cells demonstrated using intestinal biopsy tissue. AIDS 2000;14(12):1761-5.

41. Lapenta C, Boirivant M, Marini M, et al. Human intestinal lamina propria lymphocytes are naturally permissive to HIV-1 infection. European Journal of Immunology 1999;29(4):1202-8.

42. Patterson BK, Czerniewski M, Andersson J, et al. Regulation of CCR5 and CXCR4 expression by type 1 and type 2 cytokines: CCR5 expression is downregulated by IL-10 in CD4-positive lymphocytes. Clinical Immunology 1999;91(3):254-62.

43. Weissman D, Dybul M, Daucher MB, Davey RT, Jr., Walker RE, Kovacs JA. Interleukin-2 up-regulates expression of the human immunodeficiency virus fusion coreceptor CCR5 by CD4+ lymphocytes in vivo. Journal of Infectious Diseases 2000;181(3):933-8.

44. Brenchley JM, Hill BJ, Ambrozak DR, et al. T-cell subsets that harbor human immunodeficiency virus (HIV) in vivo: implications for HIV pathogenesis. Journal of Virology 2004;78(3):1160-8.

45. Brenchley JM, Price DA, Schacker TW, et al. Microbial translocation is a cause of systemic immune activation in chronic HIV infection.[see comment]. Nature Medicine 2006;12(12):1365-71.

46. Fichorova RN, Bajpai M, Chandra N, et al. Interleukin (IL)-1, IL-6, and IL-8 predict mucosal toxicity of vaginal microbicidal contraceptives. Biology of Reproduction 2004;71(3):761-9.

47. Fichorova RN, Richardson-Harman N, Alfano M, et al. Biological and technical variables affecting immunoassay recovery of cytokines from human serum and simulated vaginal fluid: a multicenter study. Analytical Chemistry 2008;80(12):4741-51.

48. McGowan I, Elliott J, Fuerst M, et al. Increased HIV-1 mucosal replication is associated with generalized mucosal cytokine activation. Journal of Acquired Immune Deficiency Syndromes: JAIDS 2004;37(2):1228-36.

49. McGowan I, Radford-Smith G, Jewell DP. Cytokine gene expression in HIV-infected intestinal mucosa. AIDS 1994;8(11):1569-75.

50. Luster AD. Chemokines--chemotactic cytokines that mediate inflammation. New England Journal of Medicine 1998;338(7):436-45.

51. MacDermott RP, Sanderson IR, Reinecker HC. The central role of chemokines (chemotactic cytokines) in the immunopathogenesis of ulcerative colitis and Crohn's disease. Inflammatory Bowel Diseases 1998;4(1):54-67.

52. Nabel G, Baltimore D. An inducible transcription factor activates expression of human immunodeficiency virus in T cells.[erratum appears in Nature 1990 Mar 8;344(6262):178]. Nature 1987;326(6114):711-3.

53. Pantaleo G, Fauci AS. Immunopathogenesis of HIV infection. Annual Review of Microbiology 1996;50:825-54.

54. Bjerke K, Halstensen TS, Jahnsen F, Pulford K, Brandtzaeg P. Distribution of macrophages and granulocytes expressing L1 protein (calprotectin) in human Peyer's patches compared with normal ileal lamina propria and mesenteric lymph nodes. Gut 1993;34(10):1357-63.

55. Johne B, Fagerhol MK, Lyberg T, et al. Functional and clinical aspects of the myelomonocyte protein calprotectin. Molecular Pathology 1997;50(3):113-23.

56. Brun JG, Ulvestad E, Fagerhol MK, Jonsson R. Effects of human calprotectin (L1) on in vitro immunoglobulin synthesis. Scandinavian Journal of Immunology 1994;40(6):675-80.

57. Yui S, Mikami M, Yamazaki M. Induction of apoptotic cell death in mouse lymphoma and human leukemia cell lines by a calcium-binding protein complex, calprotectin, derived from inflammatory peritoneal exudate cells. Journal of Leukocyte Biology 1995;58(6):650-8.

58. Roseth AG, Aadland E, Jahnsen J, Raknerud N. Assessment of disease activity in ulcerative colitis by faecal calprotectin, a novel granulocyte marker protein. Digestion 1997;58(2):176-80.

59. Tibble J, Teahon K, Thjodleifsson B, et al. A simple method for assessing intestinal inflammation in Crohn's disease. Gut 2000;47(4):506-13.

60. Thjodleifsson B, Sigthorsson G, Cariglia N, et al. Subclinical intestinal inflammation: an inherited abnormality in Crohn's disease relatives?[see comment]. Gastroenterology 2003;124(7):1728-37.

61. Patton DL, Cosgrove Sweeney YT, McCarthy TD, Hillier SL. Preclinical safety and efficacy assessments of dendrimer-based (SPL7013) microbicide gel formulations in a nonhuman primate model. Antimicrobial Agents & Chemotherapy 2006;50(5):1696-700.

62. Tien D, Schnaare RL, Kang F, et al. In vitro and in vivo characterization of a potential universal placebo designed for use in vaginal microbicide clinical trials. AIDS Research & Human Retroviruses 2005;21(10):845-53.

63. CONRAD. Investigator's Brochure: Universal Placebo Gel. 2005;Version 1.0.

64. Abner SR, Guenthner PC, Guarner J, et al. A human colorectal explant culture to evaluate topical microbicides for the prevention of HIV infection. Journal of Infectious Diseases 2005;192(9):1545-56.

65. Dezzutti CS, James VN, Ramos A, et al. In vitro comparison of topical microbicides for prevention of human immunodeficiency virus type 1 transmission. Antimicrobial Agents & Chemotherapy 2004;48(10):3834-44.

66. Patty's Industrial Hygiene and Toxicology Online. http://www3.interscience.wiley.com/cgi-bin/mrwhome/104554795/HOME; accessed 4/25/08:Section 43.4.

67. Guttner J, Klaus S, Heinecke H. Embryotoxizitat intraperitoneal applizierter Hydroxyethylcellulose bei der Maus. Anatomischer Anzeiger 1981;149(3):282-5

68. http://depts.washington.edu/terisweb/teris/index.html; accessed January 8, 2008.

69. Patton DL. A Combination Microbicide Shows Differential Toxicity Compared With Standalone Products. In: Microbicides 2008; New Delhi.

70. Patton DL, Cosgrove Sweeney YT, Paul KJ. A summary of preclinical topical microbicide rectal safety and efficacy evaluations in a pigtailed macaque model. Sexually Transmitted Diseases 2009;36(6):350-6.

71. Dreisbach R. Handbook of Poisoning. 9 ed. Los Altos, CA: Lang Medical Publications; 1977.

72. Schwartz JL, Ballagh SA, Kwok C, et al. Fourteen-day safety and acceptability study of the universal placebo gel. Contraception 2007;75(2):136-41.

73. CONRAD. Investigator's Brochure: Tenofovir Gel (GS-1278). Fourth Edition.

74. Unpublished Work, C. Dezzutti.

75. Rohan LC, Moncla BJ, Kunjara Na Ayudhya RP, et al. In Vitro and Ex Vivo Testing of Tenofovir Shows It Is Effective As an HIV-1 Microbicide. PLoS ONE 2010;5:e9310;1-13.

76. Gilead, Sciences. Viread Investigator's Brochure. September 2006.

77. Gilead, Sciences. Viread® (tenofovir disoproxil fumarate) tablets. Package Insert November 2008.

78. Gilead, Sciences. Viread Company Core Safety Information. September 18, 2006.

79. Weber J, Chakraborty B, Weberova J, Miller MD, Quinones-Mateu ME. Diminished replicative fitness of primary human immunodeficiency virus type 1 isolates harboring the K65R mutation. Journal of Clinical Microbiology 2005;43:1395-400.

80. Fairchild D. 14-day vaginal tissue irritation and toxicity study of PMPA in rats. Report No M016-97.

81. Lund B, Weber S. Pharmacokinetics and distribution of intravaginal [14C] PMPA (GS-1278) following single dose administration to rats. Gilead Sciences Report No 96-DDM-1278-004.

82. Creek MR. Pharmacokinetics and tissue distribution study of PMPA (GS-1278) following intravaginal administration in female New Zealand White Rabbits. SRI Report No B010-97.

83. Cranage M, Sharpe S, Herrera C, et al. Prevention of SIV Rectal Transmission and Priming of T Cell Responses in Macaques after Local Pre-exposure Application of Tenofovir Gel. PLoS Medicine 2008;5:1-13.

84. Rezk NL, Crutchley RD, Kashuba AD. Simultaneous quantification of emtricitabine and tenofovir in human plasma using high-performance liquid chromatography after solid phase extraction. Journal of Chromatography B: Analytical Technologies in the Biomedical & Life Sciences 2005;822:201-8.

85. Balzarini J, Van Herrewege Y, Vanham G. Metabolic activation of nucleoside and nucleotide reverse transcriptase inhibitors in dendritic and Langerhans cells. AIDS 2002;16:2159-63.

86. Delaney WEt, Ray AS, Yang H, et al. Intracellular metabolism and in vitro activity of tenofovir against hepatitis B virus. Antimicrobial Agents & Chemotherapy 2006;50:2471-7.

87. Robbins BL, Srinivas RV, Kim C, Bischofberger N, Fridland A. Anti-human immunodeficiency virus activity and cellular metabolism of a potential prodrug of the acyclic nucleoside

phosphonate 9-R-(2-phosphonomethoxypropyl)adenine (PMPA), Bis(isopropyloxymethylcarbonyl)PMPA. Antimicrobial Agents & Chemotherapy 1998;42:612-7.

88. Unpublished Work, R. Black.

89. Mayer KH, Maslankowski LA, Gai F, et al. Safety and tolerability of tenofovir vaginal gel in abstinent and sexually active HIV-infected and uninfected women. AIDS 2006;20:543-51.

90. Beigi R, Noguchi L, Macio I, et al. Maternal Single-Dose Pharmacokinetics and Placental Transfer of Tenofovir 1% Vaginal Gel Among Healthy Term Gravidas In: Microbicides 2010; 2010; Pittsburgh, PA; 2010.

91. CONRAD. Protocol #A04-099 (Study Report), Male Tolerance Study.

92. Hillier SL. Safety and acceptability of daily and coitally dependent use of 1% tenofovir over six months of use. In: Microbicides 2008; New Delhi.

93. Gatto NM, Frucht H, Sundararajan V, Jacobson JS, Grann VR, Neugut AI. Risk of perforation after colonoscopy and sigmoidoscopy: a population-based study.[see comment]. Journal of the National Cancer Institute 2003;95:230-6.

94. Wilson CM, Houser J, Partlow C, Rudy BJ, Futterman DC, Friedman LB. The REACH (Reaching for Excellence in Adolescent Care and Health) project: study design, methods, and population profile. J Adolesc Health 2001 Sep;29(3 Suppl):8-18.

95. Vermund SH, Wilson CM, Rogers AS, Partlow C, Moscicki AB. Sexually transmitted infections among HIV infected and HIV uninfected high-risk youth in the REACH study. Reaching for Excellence in Adolescent Care and Health. J Adolesc Health 2001 Sep;29(3 Suppl):49-56.

96. Moscicki AB, Durako SJ, Houser J, Ma Y, Murphy DA, Darragh TM, et al. Human papillomavirus infection and abnormal cytology of the anus in HIV-infected and uninfected adolescents. AIDS 2003 Feb 14;17(3):311-20.

97. Hogarty K, Kasowitz A, Herold BC, Keller MJ. Assessment of adherence to product dosing in a pilot microbicide study. Sex Transm Dis 2007 Dec;34(12):1000-3.

98. Bingenheimer JB, Raudenbush SW. Statistical and substantive inferences in public health: issues in the application of multilevel models. Annu Rev Public Health 2004;25:53-77.

99. Vickers AJ. The use of percentage change from baseline as an outcome in a controlled trial is statistically inefficient: a simulation study. BMC Med Res Methodol 2001;1:6.

100. Westfall PH, Young SS. Resampling-based Multiple Testing. New York,NY: Wiley; 1993.

101. Gaya DR, Lyon TD, Duncan A, Neilly JB, Han S, Howell J, et al. Faecal calprotectin in the assessment of Crohn's disease activity. QJM 2005 Jun;98(6):435-41.

102. Langhorst J, Elsenbruch S, Mueller T, Rueffer A, Spahn G, Michalsen A, et al. Comparison of 4 neutrophil-derived proteins in feces as indicators of disease activity in ulcerative colitis. Inflamm Bowel Dis 2005 Dec;11(12):1085-91.

APPENDIX I: SCHEDULE OF STUDY VISITS AND EVALUATIONS

	Stage 1A	Stage 1B					Stage 2							
		Adherence & Acceptability					Phase 1 Rectal Safety and Acceptability							
	Screening/ Enrollment	Screening/ Enrollment	Wk 1-6	Mid trial	Wk 7-12	Final	Screening†	Enrollment	Tx 1	Phone Call	Tx 2 Clearance	Tx 2 (7d)	Final	Phone Call
**Visit**	**1**	**2**		**3**		**4**	**5**	**6**	**7**	**8**	**9**	**Outpt**	**10**	**11**
**Recruitment**														
Eligibility determination	X	X					X							
Informed consent	X	X					X							
Locator Info	X	X		X		X	X	X	X	X	X		X	
**Medical evaluation**														
Med history	X	X		X		X	X	X	X		X		X	
AE/ concomitant med assessment		X		X		X	X	X	X	X	X		X	X
Physical exam	X	▲		■		X	X	■	■		■		■	
Digital rectal exam	X	▲		X		X	X	X	X		X		X	
Anoscopy*	X	▲		X		X	X	X	X		X		X	
Biopsies								X	X				X	
**Safety labs**														
CBC with differential and platelets	X	▲		▲		X	X	▲	▲		▲		X	
BUN, creatinine, ALT, AST	X	▲		▲		X	X	▲	▲		▲		X	
Electrolyte panel	X	▲		▲		X	X	▲	▲		▲		X	
PT/INR		▲**					▲							
**STI screen**														
HIV rapid test	X	▲		▲		X	X	▲	▲		▲		X	
Blood sample for confirmatory HIV testing prn	X	▲		▲		X	X	▲	▲		▲		X	
RPR	X	▲		▲		X	X	▲	▲		▲		X	
GC/CT (urine and rectal)	X	▲		X		X	X	▲	▲		▲		X	
HPV (oral and rectal)	X					X								
HSV serology	X	▲					X							
Hepatitis BsAg	X	▲		▲		X	X	▲	▲		▲		X	
Hepatitis C Ab	X	▲												
**Mucosal Assays**														
Cytokine expression								X	X				X	
Histopathology								X	X				X	
MMC phenotype								X	X				X	
Cytokine mRNA profile								X	X				X	
**Behavioral**														
Web-based CASI	X					X							X	
Video TC		X				X					X		X	
Phone reporting			X	X	X							X		
Email to Alex			X		X							X		
Condom counseling	X	X		X		X	X	X	X		X		X	
**Study Product**														
Product dispensed		X		X					X		X			
Product Use			X		X				X			X		
Collect applicators				X		X							X	

* HRA will be performed at the Pittsburgh and Boston sites in addition to standard anoscopy at Visit 1 (non sex-workers only)
† Visit 5 & 6 can be combined, if eligibility can be determined based on current (i.e., within last 28 days) laboratory results, physical exam, and anoscopy on file at the site from Stage 1AB. In that case it will not be necessary to repeat the laboratory tests, physical examination, or anoscopy.
▲ If clinically indicated; ▲** If clinically indicated, non sex-workers only) ■ Symptom directed

APPENDIX II: TOXICITY TABLES

The DAIDS AE Grading Table Version 1.0, December 2004, Addendum 3 (Rectal Grading Table for Use in Microbicide Studies) will be the primary tool for grading adverse events for this protocol. Adverse events not included in that table will be graded by the DAIDS AE Grading Table Version 1.0, December 2004. In cases where an AE is covered in both tables, Addendum 3 (Rectal Grading Table for Use in Microbicide Studies) will be the grading scale utilized.

The Division of AIDS Table for Grading the Severity of Adult and Pediatric Adverse Events (DAIDS AE Grading Table), Version 1.0, December, 2004, is available online at http://rcc.tech-res-intl.com/tox_tables.htm

APPENDIX III:
HISTOPATHOLOGY SCORING SYSTEM

Participant ID: _________ Visit No.: _______ Visit Date: ______

Please Circle the Grade	Please Circle Subgrade	
**Grade 0**<br>**No abnormality**		
**Grade 1**<br>**Mononuclear cell infiltrate**	**Low**	**High**
**Grade 2**<br>**Neutrophilic infiltrate-lamina propria**	**Low**	**High**
**Grade 3**<br>**Neutrophilic infiltrate-epithelium**	**Low**<br>***< 50% of crypts***	**High**<br>***> 50% of crypts***
**Grade 4**<br>**Crypt destruction**	**Low**<br>***probable***	**High**<br>***unequivocal***
**Grade 5**<br>**Erosion or ulceration**	**Low**<br>***Restitution, probable erosion***	**High**<br>***Unequivocal erosion or ulceration***
