# Supplementary material for: Project Gel a Randomized Rectal Microbicide Safety and Acceptability Study in Young Men and Transgender Women
Source: PLoS One. 2016 Jun 30;11(6):e0158310. doi: 10.1371/journal.pone.0158310 (PMC4928823; doi:10.1371/journal.pone.0158310)
Supplement: S1 Table — (DOCX) [file pone.0158310.s003.docx]

**Supplementary Table 1** Project Gel Visit Schedule

| **Stage** | **Visit Number** | **Purpose of Visit** |
| --- | --- | --- |
|  |  |  |
| 1A | 1 | Stage 1A Visit; informed consent, demographics, behavioral assessment, physical examination and STI screen |
|  |  |  |
| Stage 1B | 2 | Stage 1B enrollment visit; informed consent, demographics, behavioral assessment, physical examination*, STI screen*, and provision of placebo applicators |
|  | 3 | Stage 1B Mid-Trial Visit; behavioral assessment, physical examination, STI screen*, and provision of placebo applicators |
|  | 4 | Stage 1B Final Visit; behavioral assessment, physical examination and STI screen |
|  |  |  |
| Stage 2 | 5 | Stage 2 Screening Visit*; physical examination and STI screen |
|  | 6 | Stage 2 Enrollment Visit*; physical examination, STI screen*, and collection of baseline mucosal samples |
|  | 7 | Stage 2 Single Dose Administration Visit; physical examination, STI screen*, administration of study product, anoscopy and collection of rectal biopsies |
|  | 8 | Stage 2 Follow-up Phone Call; adverse event and concomitant medication assessment |
|  | 9 | Stage 2 Multiple Dose Administration Visit; physical examination, STI screen* |
|  | 10 | Stage 2 Final Visit; physical examination, STI screen, administration of final study product, anoscopy and collection of rectal biopsies |
|  | 11 | Stage 2 Follow-up Phone Call; adverse event and concomitant medication assessment |

*If indicated
