# Supplementary material for: Project Gel a Randomized Rectal Microbicide Safety and Acceptability Study in Young Men and Transgender Women
Source: PLoS One. 2016 Jun 30;11(6):e0158310. doi: 10.1371/journal.pone.0158310 (PMC4928823; doi:10.1371/journal.pone.0158310)
Supplement: S2 Table — (DOCX) [file pone.0158310.s004.docx]

**Supplementary Table 2:** Stage 2 Adverse events summary by system and by arm

| **Adverse Events** | **Overall**  **(N = 24)** | **Tenofovir**  **(N = 12)** | **Placebo**  **(N = 12)** |
| --- | --- | --- | --- |
|  |  |  |  |
| **Cardiac disorders** |  |  |  |
| - Grade 1 | 1 (100%) | 1 (100%) | 0 (0.00%) |
| - Grade 2 | 0 (0.00%) | 0 (0.00%) | 0 (0.00%) |
| - Grade 3 | 0 (0.00%) | 0 (0.00%) | 0 (0.00%) |
| - Grade 4 | 0 (0.00%) | 0 (0.00%) | 0 (0.00%) |
| - Total | 1 (100%) | 1 (100%) | 0 (0.00%) |
|  |  |  |  |
| *Palpitations* |  |  |  |
| - Grade 1 | 1 (100%) | 1 (100%) | 0 (0.00%) |
| - Grade 2 | 0 (0.00%) | 0 (0.00%) | 0 (0.00%) |
| - Grade 3 | 0 (0.00%) | 0 (0.00%) | 0 (0.00%) |
| - Grade 4 | 0 (0.00%) | 0 (0.00%) | 0 (0.00%) |
| - Total | 1 (100%) | 1 (100%) | 0 (0.00%) |
|  |  |  |  |
| **Eye disorders** |  |  |  |
| - Grade 1 | 1 (100%) | 1 (100%) | 0 (0.00%) |
| - Grade 2 | 0 (0.00%) | 0 (0.00%) | 0 (0.00%) |
| - Grade 3 | 0 (0.00%) | 0 (0.00%) | 0 (0.00%) |
| - Grade 4 | 0 (0.00%) | 0 (0.00%) | 0 (0.00%) |
| - Total | 1 (100%) | 1 (100%) | 0 (0.00%) |

|  |  |  |  |
| --- | --- | --- | --- |
| *Conjunctival irritation* |  |  |  |
| - Grade 1 | 1 (100%) | 1 (100%) | 0 (0.00%) |
| - Grade 2 | 0 (0.00%) | 0 (0.00%) | 0 (0.00%) |
| - Grade 3 | 0 (0.00%) | 0 (0.00%) | 0 (0.00%) |
| - Grade 4 | 0 (0.00%) | 0 (0.00%) | 0 (0.00%) |
| - Total | 1 (100%) | 1 (100%) | 0 (0.00%) |
|  |  |  |  |
| **Gastrointestinal disorders** |  |  |  |
| - Grade 1 | 9 (90%) | 6 (85.71%) | 3 (100%) |
| - Grade 2 | 0 (0.00%) | 0 (0.00%) | 0 (0.00%) |
| - Grade 3 | 1 (10%) | 1 (14.29%) | 0 (0.00%) |
| - Grade 4 | 0 (0.00%) | 0 (0.00%) | 0 (0.00%) |
| - Total | 10 (100%) | 7 (100%) | 3 (100%) |
|  |  |  |  |
| *Faeces discoloure* |  |  |  |
| - Grade 1 | 1 (100%) | 1 (100%) | 0 (0.00%) |
| - Grade 2 | 0 (0.00%) | 0 (0.00%) | 0 (0.00%) |
| - Grade 3 | 0 (0.00%) | 0 (0.00%) | 0 (0.00%) |
| - Grade 4 | 0 (0.00%) | 0 (0.00%) | 0 (0.00%) |
| - Total | 1 (100%) | 1 (100%) | 0 (0.00%) |
|  |  |  |  |
| *Haematochezia* |  |  |  |
| - Grade 1 | 1 (100%) | 1 (100%) | 0 (0.00%) |
| - Grade 2 | 0 (0.00%) | 0 (0.00%) | 0 (0.00%) |
| - Grade 3 | 0 (0.00%) | 0 (0.00%) | 0 (0.00%) |
| - Grade 4 | 0 (0.00%) | 0 (0.00%) | 0 (0.00%) |
| - Total | 1 (100%) | 1 (100%) | 0 (0.00%) |
|  |  |  |  |
| *Anal haemorrhage* |  |  |  |
| - Grade 1 | 1 (100%) | 0 (0.00%) | 1 (100%) |
| - Grade 2 | 0 (0.00%) | 0 (0.00%) | 0 (0.00%) |
| - Grade 3 | 0 (0.00%) | 0 (0.00%) | 0 (0.00%) |
| - Grade 4 | 0 (0.00%) | 0 (0.00%) | 0 (0.00%) |
| - Total | 1 (100%) | 0 (0.00%) | 1 (100%) |
|  |  |  |  |
| *Diarrhoea* |  |  |  |
| - Grade 1 | 2 (100%) | 1 (100%) | 1 (100%) |
| - Grade 2 | 0 (0.00%) | 0 (0.00%) | 0 (0.00%) |
| - Grade 3 | 0 (0.00%) | 0 (0.00%) | 0 (0.00%) |
| - Grade 4 | 0 (0.00%) | 0 (0.00%) | 0 (0.00%) |
| - Total | 2 (100%) | 1 (100%) | 1 (100%) |
|  |  |  |  |
| *Vomiting* |  |  |  |
| - Grade 1 | 1 (100%) | 1 (100%) | 0 (0.00%) |
| - Grade 2 | 0 (0.00%) | 0 (0.00%) | 0 (0.00%) |
| - Grade 3 | 0 (0.00%) | 0 (0.00%) | 0 (0.00%) |
| - Grade 4 | 0 (0.00%) | 0 (0.00%) | 0 (0.00%) |
| - Total | 1 (100%) | 1 (100%) | 0 (0.00%) |
|  |  |  |  |
| *Painful defaecation* |  |  |  |
| - Grade 1 | 1 (100%) | 1 (100%) | 0 (0.00%) |
| - Grade 2 | 0 (0.00%) | 0 (0.00%) | 0 (0.00%) |
| - Grade 3 | 0 (0.00%) | 0 (0.00%) | 0 (0.00%) |
| - Grade 4 | 0 (0.00%) | 0 (0.00%) | 0 (0.00%) |
| - Total | 1 (100%) | 1 (100%) | 0 (0.00%) |
|  |  |  |  |
| *Proctalgia* |  |  |  |
| - Grade 1 | 1 (100%) | 1 (100%) | 0 (0.00%) |
| - Grade 2 | 0 (0.00%) | 0 (0.00%) | 0 (0.00%) |
| - Grade 3 | 0 (0.00%) | 0 (0.00%) | 0 (0.00%) |
| - Grade 4 | 0 (0.00%) | 0 (0.00%) | 0 (0.00%) |
| - Total | 1 (100%) | 1 (100%) | 0 (0.00%) |
|  |  |  |  |
| *Abdominal pain* |  |  |  |
| - Grade 1 | 0 (0.00%) | 0 (0.00%) | 0 (0.00%) |
| - Grade 2 | 0 (0.00%) | 0 (0.00%) | 0 (0.00%) |
| - Grade 3 | 1 (100%) | 1 (100%) | 0 (0.00%) |
| - Grade 4 | 0 (0.00%) | 0 (0.00%) | 0 (0.00%) |
| - Total | 1 (100%) | 1 (100%) | 0 (0.00%) |
|  |  |  |  |
| *Abdominal discomfort* |  |  |  |
| - Grade 1 | 1 (100%) | 0 (0.00%) | 1 (100%) |
| - Grade 2 | 0 (0.00%) | 0 (0.00%) | 0 (0.00%) |
| - Grade 3 | 0 (0.00%) | 0 (0.00%) | 0 (0.00%) |
| - Grade 4 | 0 (0.00%) | 0 (0.00%) | 0 (0.00%) |
| - Total | 1 (100%) | 0 (0.00%) | 1 (100%) |

|  |  |  |  |
| --- | --- | --- | --- |
| **General disorders and administration site conditions** |  |  |  |
| - Grade 1 | 3 (100%) | 2 (100%) | 1 (100%) |
| - Grade 2 | 0 (0.00%) | 0 (0.00%) | 0 (0.00%) |
| - Grade 3 | 0 (0.00%) | 0 (0.00%) | 0 (0.00%) |
| - Grade 4 | 0 (0.00%) | 0 (0.00%) | 0 (0.00%) |
| - Total | 3 (100%) | 2 (100%) | 1 (100%) |
|  |  |  |  |
| *Application site paraesthesia* |  |  |  |
| - Grade 1 | 1 (100%) | 1 (100%) | 0 (0.00%) |
| - Grade 2 | 0 (0.00%) | 0 (0.00%) | 0 (0.00%) |
| - Grade 3 | 0 (0.00%) | 0 (0.00%) | 0 (0.00%) |
| - Grade 4 | 0 (0.00%) | 0 (0.00%) | 0 (0.00%) |
| - Total | 1 (100%) | 1 (100%) | 0 (0.00%) |
|  |  |  |  |
| *Chills* |  |  |  |
| - Grade 1 | 1 (100%) | 1 (100%) | 0 (0.00%) |
| - Grade 2 | 0 (0.00%) | 0 (0.00%) | 0 (0.00%) |
| - Grade 3 | 0 (0.00%) | 0 (0.00%) | 0 (0.00%) |
| - Grade 4 | 0 (0.00%) | 0 (0.00%) | 0 (0.00%) |
| - Total | 1 (100%) | 1 (100%) | 0 (0.00%) |
|  |  |  |  |
| *Fatigue* |  |  |  |
| - Grade 1 | 1 (100%) | 0 (0.00%) | 1 (100%) |
| - Grade 2 | 0 (0.00%) | 0 (0.00%) | 0 (0.00%) |
| - Grade 3 | 0 (0.00%) | 0 (0.00%) | 0 (0.00%) |
| - Grade 4 | 0 (0.00%) | 0 (0.00%) | 0 (0.00%) |
| - Total | 1 (100%) | 0 (0.00%) | 1 (100%) |

|  |  |  |  |
| --- | --- | --- | --- |
| **Infections and infestations** |  |  |  |
| - Grade 1 | 2 (50%) | 1 (33.33%) | 1 (100%) |
| - Grade 2 | 2 (50%) | 2 (66.67%) | 0 (0.00%) |
| - Grade 3 | 0 (0.00%) | 0 (0.00%) | 0 (0.00%) |
| - Grade 4 | 0 (0.00%) | 0 (0.00%) | 0 (0.00%) |
| - Total | 4 (100%) | 3 (100%) | 1 (100%) |
|  |  |  |  |
| *Upper respiratory tract infection* |  |  |  |
| - Grade 1 | 1 (100%) | 1 (100%) | 0 (0.00%) |
| - Grade 2 | 0 (0.00%) | 0 (0.00%) | 0 (0.00%) |
| - Grade 3 | 0 (0.00%) | 0 (0.00%) | 0 (0.00%) |
| - Grade 4 | 0 (0.00%) | 0 (0.00%) | 0 (0.00%) |
| - Total | 1 (100%) | 1 (100%) | 0 (0.00%) |
|  |  |  |  |
| *Nasopharyngitis* |  |  |  |
| - Grade 1 | 1 (50%) | 0 (0.00%) | 1 (100%) |
| - Grade 2 | 1 (50%) | 1 (100%) | 0 (0.00%) |
| - Grade 3 | 0 (0.00%) | 0 (0.00%) | 0 (0.00%) |
| - Grade 4 | 0 (0.00%) | 0 (0.00%) | 0 (0.00%) |
| - Total | 2 (100%) | 1 (100%) | 1 (100%) |
|  |  |  |  |
| *Genitourinary chlamydia infection* |  |  |  |
| - Grade 1 | 0 (0.00%) | 0 (0.00%) | 0 (0.00%) |
| - Grade 2 | 1 (100%) | 1 (100%) | 0 (0.00%) |
| - Grade 3 | 0 (0.00%) | 0 (0.00%) | 0 (0.00%) |
| - Grade 4 | 0 (0.00%) | 0 (0.00%) | 0 (0.00%) |
| - Total | 1 (100%) | 1 (100%) | 0 (0.00%) |

|  |  |  |  |
| --- | --- | --- | --- |
| **Injury, poisoning and procedural complications** |  |  |  |
| - Grade 1 | 4 (100%) | 0 (0.00%) | 4 (100%) |
| - Grade 2 | 0 (0.00%) | 0 (0.00%) | 0 (0.00%) |
| - Grade 3 | 0 (0.00%) | 0 (0.00%) | 0 (0.00%) |
| - Grade 4 | 0 (0.00%) | 0 (0.00%) | 0 (0.00%) |
| - Total | 4 (100%) | 0 (0.00%) | 4 (100%) |
|  |  |  |  |
| *Procedural pain* |  |  |  |
| - Grade 1 | 1 (100%) | 0 (0.00%) | 1 (100%) |
| - Grade 2 | 0 (0.00%) | 0 (0.00%) | 0 (0.00%) |
| - Grade 3 | 0 (0.00%) | 0 (0.00%) | 0 (0.00%) |
| - Grade 4 | 0 (0.00%) | 0 (0.00%) | 0 (0.00%) |
| - Total | 1 (100%) | 0 (0.00%) | 1 (100%) |
|  |  |  |  |
| *Excoriation* |  |  |  |
| - Grade 1 | 1 (100%) | 0 (0.00%) | 1 (100%) |
| - Grade 2 | 0 (0.00%) | 0 (0.00%) | 0 (0.00%) |
| - Grade 3 | 0 (0.00%) | 0 (0.00%) | 0 (0.00%) |
| - Grade 4 | 0 (0.00%) | 0 (0.00%) | 0 (0.00%) |
| - Total | 1 (100%) | 0 (0.00%) | 1 (100%) |
|  |  |  |  |
| *Post procedural constipation* |  |  |  |
| - Grade 1 | 1 (100%) | 0 (0.00%) | 1 (100%) |
| - Grade 2 | 0 (0.00%) | 0 (0.00%) | 0 (0.00%) |
| - Grade 3 | 0 (0.00%) | 0 (0.00%) | 0 (0.00%) |
| - Grade 4 | 0 (0.00%) | 0 (0.00%) | 0 (0.00%) |
| - Total | 1 (100%) | 0 (0.00%) | 1 (100%) |
|  |  |  |  |
| *Post procedural haemorrhage* |  |  |  |
| - Grade 1 | 1 (100%) | 0 (0.00%) | 1 (100%) |
| - Grade 2 | 0 (0.00%) | 0 (0.00%) | 0 (0.00%) |
| - Grade 3 | 0 (0.00%) | 0 (0.00%) | 0 (0.00%) |
| - Grade 4 | 0 (0.00%) | 0 (0.00%) | 0 (0.00%) |
| - Total | 1 (100%) | 0 (0.00%) | 1 (100%) |
|  |  |  |  |
| **Investigations** |  |  |  |
| - Grade 1 | 2 (100%) | 1 (100%) | 1 (100%) |
| - Grade 2 | 0 (0.00%) | 0 (0.00%) | 0 (0.00%) |
| - Grade 3 | 0 (0.00%) | 0 (0.00%) | 0 (0.00%) |
| - Grade 4 | 0 (0.00%) | 0 (0.00%) | 0 (0.00%) |
| - Total | 2 (100%) | 1 (100%) | 1 (100%) |
|  |  |  |  |
| *Aspartate aminotransferase increase* |  |  |  |
| - Grade 1 | 2 (100%) | 1 (100%) | 1 (100%) |
| - Grade 2 | 0 (0.00%) | 0 (0.00%) | 0 (0.00%) |
| - Grade 3 | 0 (0.00%) | 0 (0.00%) | 0 (0.00%) |
| - Grade 4 | 0 (0.00%) | 0 (0.00%) | 0 (0.00%) |
| - Total | 2 (100%) | 1 (100%) | 1 (100%) |
|  |  |  |  |
| **Musculoskeletal and connective tissue disorders** |  |  |  |
| - Grade 1 | 3 (75%) | 2 (100%) | 1 (50%) |
| - Grade 2 | 1 (25%) | 0 (0.00%) | 1 (50%) |
| - Grade 3 | 0 (0.00%) | 0 (0.00%) | 0 (0.00%) |
| - Grade 4 | 0 (0.00%) | 0 (0.00%) | 0 (0.00%) |
| - Total | 4 (100%) | 2 (100%) | 2 (100%) |
|  |  |  |  |
| *Myalgia* |  |  |  |
| - Grade 1 | 1 (100%) | 1 (100%) | 0 (0.00%) |
| - Grade 2 | 0 (0.00%) | 0 (0.00%) | 0 (0.00%) |
| - Grade 3 | 0 (0.00%) | 0 (0.00%) | 0 (0.00%) |
| - Grade 4 | 0 (0.00%) | 0 (0.00%) | 0 (0.00%) |
| - Total | 1 (100%) | 1 (100%) | 0 (0.00%) |
|  |  |  |  |
| *Arthralgia* |  |  |  |
| - Grade 1 | 1 (100%) | 1 (100%) | 0 (0.00%) |
| - Grade 2 | 0 (0.00%) | 0 (0.00%) | 0 (0.00%) |
| - Grade 3 | 0 (0.00%) | 0 (0.00%) | 0 (0.00%) |
| - Grade 4 | 0 (0.00%) | 0 (0.00%) | 0 (0.00%) |
| - Total | 1 (100%) | 1 (100%) | 0 (0.00%) |
|  |  |  |  |
| *Back pain* |  |  |  |
| - Grade 1 | 1 (50%) | 0 (0.00%) | 1 (50%) |
| - Grade 2 | 1 (50%) | 0 (0.00%) | 1 (50%) |
| - Grade 3 | 0 (0.00%) | 0 (0.00%) | 0 (0.00%) |
| - Grade 4 | 0 (0.00%) | 0 (0.00%) | 0 (0.00%) |
| - Total | 2 (100%) | 0 (0.00%) | 2 (100%) |
|  |  |  |  |
| **Neoplasms** |  |  |  |
| - Grade 1 | 1 (100%) | 1 (100%) | 0 (0.00%) |
| - Grade 2 | 0 (0.00%) | 0 (0.00%) | 0 (0.00%) |
| - Grade 3 | 0 (0.00%) | 0 (0.00%) | 0 (0.00%) |
| - Grade 4 | 0 (0.00%) | 0 (0.00%) | 0 (0.00%) |
| - Total | 1 (100%) | 1 (100%) | 0 (0.00%) |
|  |  |  |  |
| *Anorectal human papilloma virus infection* |  |  |  |
| - Grade 1 | 1 (100%) | 1 (100%) | 0 (0.00%) |
| - Grade 2 | 0 (0.00%) | 0 (0.00%) | 0 (0.00%) |
| - Grade 3 | 0 (0.00%) | 0 (0.00%) | 0 (0.00%) |
| - Grade 4 | 0 (0.00%) | 0 (0.00%) | 0 (0.00%) |
| - Total | 1 (100%) | 1 (100%) | 0 (0.00%) |
|  |  |  |  |
| **Nervous system disorders** |  |  |  |
| - Grade 1 | 1 (50%) | 1 (100%) | 0 (0.00%) |
| - Grade 2 | 1 (50%) | 0 (0.00%) | 1 (100%) |
| - Grade 3 | 0 (0.00%) | 0 (0.00%) | 0 (0.00%) |
| - Grade 4 | 0 (0.00%) | 0 (0.00%) | 0 (0.00%) |
| - Total | 2 (100%) | 1 (100%) | 1 (100%) |
|  |  |  |  |
| *Headache* |  |  |  |
| - Grade 1 | 1 (100%) | 1 (100%) | 0 (0.00%) |
| - Grade 2 | 0 (0.00%) | 0 (0.00%) | 0 (0.00%) |
| - Grade 3 | 0 (0.00%) | 0 (0.00%) | 0 (0.00%) |
| - Grade 4 | 0 (0.00%) | 0 (0.00%) | 0 (0.00%) |
| - Total | 1 (100%) | 1 (100%) | 0 (0.00%) |
|  |  |  |  |
| *Dizziness* |  |  |  |
| - Grade 1 | 0 (0.00%) | 0 (0.00%) | 0 (0.00%) |
| - Grade 2 | 1 (100%) | 0 (0.00%) | 1 (100%) |
| - Grade 3 | 0 (0.00%) | 0 (0.00%) | 0 (0.00%) |
| - Grade 4 | 0 (0.00%) | 0 (0.00%) | 0 (0.00%) |
| - Total | 1 (100%) | 0 (0.00%) | 1 (100%) |
|  |  |  |  |
| **Reproductive system and breast disorders** |  |  |  |
| - Grade 1 | 1 (100%) | 0 (0.00%) | 1 (100%) |
| - Grade 2 | 0 (0.00%) | 0 (0.00%) | 0 (0.00%) |
| - Grade 3 | 0 (0.00%) | 0 (0.00%) | 0 (0.00%) |
| - Grade 4 | 0 (0.00%) | 0 (0.00%) | 0 (0.00%) |
| - Total | 1 (100%) | 0 (0.00%) | 1 (100%) |
|  |  |  |  |
| *Peyronie’s disease* |  |  |  |
| - Grade 1 | 1 (100%) | 0 (0.00%) | 1 (100%) |
| - Grade 2 | 0 (0.00%) | 0 (0.00%) | 0 (0.00%) |
| - Grade 3 | 0 (0.00%) | 0 (0.00%) | 0 (0.00%) |
| - Grade 4 | 0 (0.00%) | 0 (0.00%) | 0 (0.00%) |
| - Total | 1 (100%) | 0 (0.00%) | 1 (100%) |
|  |  |  |  |
| **Respiratory, thoracic and mediastinal disorders** |  |  |  |
| - Grade 1 | 4 (100%) | 3 (100%) | 1 (100%) |
| - Grade 2 | 0 (0.00%) | 0 (0.00%) | 0 (0.00%) |
| - Grade 3 | 0 (0.00%) | 0 (0.00%) | 0 (0.00%) |
| - Grade 4 | 0 (0.00%) | 0 (0.00%) | 0 (0.00%) |
| - Total | 4 (100%) | 3 (100%) | 1 (100%) |
|  |  |  |  |
| *Oropharyngeal pain* |  |  |  |
| - Grade 1 | 1 (100%) | 1 (100%) | 0 (0.00%) |
| - Grade 2 | 0 (0.00%) | 0 (0.00%) | 0 (0.00%) |
| - Grade 3 | 0 (0.00%) | 0 (0.00%) | 0 (0.00%) |
| - Grade 4 | 0 (0.00%) | 0 (0.00%) | 0 (0.00%) |
| - Total | 1 (100%) | 1 (100%) | 0 (0.00%) |
|  |  |  |  |
| *Nasal congestion* |  |  |  |
| - Grade 1 | 1 (100%) | 1 (100%) | 0 (0.00%) |
| - Grade 2 | 0 (0.00%) | 0 (0.00%) | 0 (0.00%) |
| - Grade 3 | 0 (0.00%) | 0 (0.00%) | 0 (0.00%) |
| - Grade 4 | 0 (0.00%) | 0 (0.00%) | 0 (0.00%) |
| - Total | 1 (100%) | 1 (100%) | 0 (0.00%) |
|  |  |  |  |
| *Rhinorrhoea* |  |  |  |
| - Grade 1 | 2 (100%) | 1 (100%) | 1 (100%) |
| - Grade 2 | 0 (0.00%) | 0 (0.00%) | 0 (0.00%) |
| - Grade 3 | 0 (0.00%) | 0 (0.00%) | 0 (0.00%) |
| - Grade 4 | 0 (0.00%) | 0 (0.00%) | 0 (0.00%) |
| - Total | 2 (100%) | 1 (100%) | 1 (100%) |
|  |  |  |  |
| **Vascular disorders** |  |  |  |
| - Grade 1 | 2 (100%) | 2 (100%) | 0 (0.00%) |
| - Grade 2 | 0 (0.00%) | 0 (0.00%) | 0 (0.00%) |
| - Grade 3 | 0 (0.00%) | 0 (0.00%) | 0 (0.00%) |
| - Grade 4 | 0 (0.00%) | 0 (0.00%) | 0 (0.00%) |
| - Total | 2 (100%) | 2 (100%) | 0 (0.00%) |

|  |  |  |  |
| --- | --- | --- | --- |
| *Hypertension* |  |  |  |
| - Grade 1 | 2 (100%) | 2 (100%) | 0 (0.00%) |
| - Grade 2 | 0 (0.00%) | 0 (0.00%) | 0 (0.00%) |
| - Grade 3 | 0 (0.00%) | 0 (0.00%) | 0 (0.00%) |
| - Grade 4 | 0 (0.00%) | 0 (0.00%) | 0 (0.00%) |
| - Total | 2 (100%) | 2 (100%) | 0 (0.00%) |
